# Tuning the Molecular Rotor Dynamics of Pyrrolo[3,4‐*b*]Pyridin‐5‐Ones: Steric and Electronic Control of Environmental Sensing

**DOI:** 10.1002/bio.70655

**Published:** 2026-10-21

**Authors:** Ivette Morales‐Salazar, Carlos E. Garduño Albino, Alejandro Valderrama‐Celestino, Federico González‐García, Alberto Rojas, Annia Galano, Yoarhy A. Amador‐Sánchez, Cesar A. Guarin, Eduardo González‐Zamora, Alejandro Islas‐Jácome

**Affiliations:** ^1^ Departamento de Química Universidad Autónoma Metropolitana‐Iztapalapa Ciudad de México Mexico; ^2^ Department of Chemistry and Biochemistry Baylor University Waco Texas USA; ^3^ Departamento de Ingeniería de Procesos e Hidráulica Universidad Autónoma Metropolitana‐Iztapalapa Ciudad de México Mexico; ^4^ Departamento de Física Universidad Autónoma Metropolitana‐Iztapalapa Ciudad de México Mexico

**Keywords:** DFT‐based calculations, multicomponent reactions, photophysical properties, pyrrolo[3,4‐*b*]pyridin‐5‐ones, sensing applications

## Abstract

A series of seven pyrrolo[3,4‐*b*]pyridin‐5‐ones were synthesized via a one‐pot process involving an Ugi‐Zhu reaction followed by a cascade sequence (*N*‐acylation/*aza* Diels–Alder cycloaddition/decarboxylation/dehydration) incorporating different substituents no aromatic and aromatic such as furan, tetrahydrofuran, and pyridine. The pyrrolo[3,4‐*b*]pyridin‐5‐ones were optically characterized through their photoluminescence spectra, quantum yields, and fluorescence lifetimes. By combining spectroscopic measurements with DFT/TDDFT calculations, photophysical behavior was elucidated, specifically addressing solvent effects on transition magnitudes and the electronic nature of absorption and emission states. Structure–property relationship analyses revealed that steric and electronic substituent effects govern the interplay between charge‐transfer (CT) and locally excited (LE) states. Finally, the most promising candidates were successfully evaluated as fluorogenic microviscosity and polarity sensors, demonstrating their potential for advanced sensing applications.

## Graphical abstract

Pyrrolo[3,4‐*b*]pyridin‐5‐ones are assembled in one pot manner through an Ugi‐Zhu reaction coupled to a cascade sequence (aza Diels‐Alder/*N*‐acylation/decarboxylation/dehydration). Pyrrolo[3,4‐*b*]pyridin‐5‐ones are fluorescence probes for local viscosity, polarity, protic environments, and solid‐state emission.

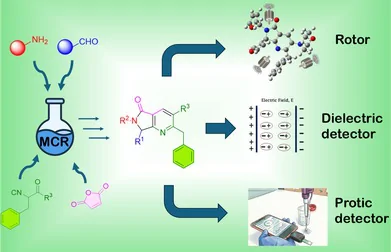

## 1 Introduction

Fluorescent organic materials have attracted considerable attention from the scientific community due to their versatile synthetic accessibility and tunable photophysical properties in solution [1], the solid state [2], or both (dual‐state emitters, DSEs) [3]. Their molecular structures can be readily engineered through a wide range of synthetic methodologies, enabling precise modulation of chromophoric systems for applications in materials science, including fluorescent probes [4], organic light‐emitting diodes (OLEDs) [5], and organic semiconductors for optoelectronic devices [6]. In this context, the development of efficient expedited synthetic strategies is highly desirable for rapid access to structurally diverse fluorophores with well‐defined controllable properties. In this context, multicomponent reactions (MCRs) have emerged as powerful synthetic strategies for the rapid construction of complex molecular architectures by combining three or more substrates in a single operational step, offering high atom economy, a wide range of structural diversity, reduced reaction times, and operational simplicity [7, 8, 9]. These features make MCRs particularly attractive for the synthesis of polyheterocyclic systems with potential biological [10] and optoelectronic applications [11]. Among these, compounds having the pyrrolo[3,4‐*b*]pyridin‐5‐one core have been studied in our research group as anticancer [12], antiviral [13], or dual‐state emission materials [14]. Structurally, this synthetic scaffold is an aza‐analogue of the natural motif isoindolin‐1‐one, and both systems contain extended π‐conjugated frameworks, commonly associated with interesting optical properties [15]. Indeed, previous studies on isoindolin‐1‐ones have demonstrated their potential as fluorescent materials. Furan can be considered fragment‐privileged, found in different compounds such as natural products and drugs (e.g., nitrofurantoin) as well as chemosensors and fluorescent probes. In the same way, pyridine derivatives play the role of a fluorescent probe, where the position of the nitrogen is important for the photophysical properties [16, 17, 18].

From a molecular design perspective, two principal strategies have been described for the development of fluorescent compounds via MCRs: the scaffold approach, in which a preformed chromophore is incorporated into one (or more) starting reagents of the multicomponent reaction, and the chromophore approach, where the emissive unit is generated during the multicomponent reaction itself [11]. In both cases, the incorporation of auxochromic substituents such as tetrahydrofuran plays a key role in fine‐tuning the electronic structure and photophysical response of the resulting molecules. In this regard, morpholine‐containing groups represent particularly valuable functional motifs due to their combined electronic and supramolecular properties. The morpholine unit contains both a tertiary amine and an ether oxygen, enabling it to participate in hydrogen‐bonding interactions [19] with the surrounding environment, primarily as a hydrogen‐bond acceptor [20]. These interactions can influence fluorescence behavior by stabilizing excited states and modulating nonradiative decay pathways. Additionally, the presence of lone electron pairs allows morpholine to act as a weak electron‐donating auxochrome, contributing to the modulation of HOMO–LUMO energy levels and the balance between locally excited (LE) and charge‐transfer (CT) states [21]. Importantly, morpholine derivatives have also been widely employed in fluorescent probes due to their ability to undergo protonation and promote selective accumulation in acidic environments such as lysosomes, further highlighting their relevance for sensing applications [22].

Herein, we analyzed a series of seven pyrrolo[3,4‐*b*]pyridin‐5‐ones (**1a–g**) synthesized via a one‐pot multicomponent Ugi‐Zhu reaction coupled to a cascade sequence [*N*‐acylation/aza Diels‐Alder cycloaddition/decarboxylation/dehydration]. The compounds were characterized comprehensively through their photophysical properties, including photoluminescence spectra, quantum yields, and fluorescence lifetimes. Furthermore, density functional theory (DFT) and time‐dependent DFT (TD‐DFT)–based calculations were performed to elucidate the electronic nature of the absorption and emission processes, as well as solvent effects. Structure–property relationship analyses revealed how steric and electronic substituents govern the interplay between charge transfer and local excited states. Finally, selected compounds were evaluated as fluorogenic microviscosity, proticity, and as polarity sensors, demonstrating their potential for advanced sensing applications.

## 2 Experimental

### 2.1 Computational Details

All electronic structure calculations were carried out using the Gaussian 09 software suite [23]. For each molecule, initial guess structures were subjected to full geometry optimizations and frequency calculations to reach their equilibrium geometries within each solvent environment, without imposing any symmetry constraints. These ground‐state calculations utilized Density Functional Theory (DFT) with the B3LYP functional in conjunction with the 6–31 + G(d) basis set. Solvation effects were accounted for using the solvation model based on density (SMD) [24] with methanol, acetonitrile, and toluene to support the experimental data. Local minima were verified by the absence of imaginary frequencies. From these optimized structures, frontier molecular orbitals (FMOs) were analyzed, and relative energies were computed including thermodynamic corrections at 298.15 K. Subsequently, singlet excited states and UV–Vis absorption spectra were simulated via time‐dependent DFT (TD‐DFT) at the same level of theory.

### 2.2 Optical Studies

#### 2.2.1 Absorption and Photoluminescence Spectra

Solutions were prepared in organic solvents at a concentration of approximately 5 × 10^−5^ M for all compounds. UV–Visible absorption and photoluminescence spectra were recorded using a Thermo‐Scientific Evolution 220 and a Perkin‐Elmer LS50B spectrophotometer, respectively, using 1‐cm path‐length quartz cells. All measurements were performed at 21°C using freshly prepared, aerated solutions.

#### 2.2.2 Quantum Yield

For the absolute quantum yield measurements, an FL 8500 Fluorescence Spectrophotometer (Perkin‐Elmer) at high resolution (0.2 nm), equipped with a 100 mm in diameter coated integrating sphere and 1‐cm path‐length quartz cell, was employed. Quantum yield was determined by the Mello method in methanol, acetonitrile, and toluene solvents at an approximate 5 × 10^−5^ M concentration for Compounds **1a–g** [25]. The measurements were performed with freshly prepared solutions, under aerated conditions, at 21°C. Throughout the text, photoluminescence quantum yield (%*Φ*_*f*_) are expressed as percentages.

#### 2.2.3 Fluorescence Lifetimes

Fluorescence lifetimes for the pyrrolo[3,4*‐b*]pyridin‐5‐ones (**1a–g**) in condensed media were determined via the Time‐Correlated Single Photon Counting (TCSPC) technique. TCSPC measurements were performed in a configuration similar to that implemented in Ref [26]. Briefly, the pyrrolo[3,4‐*b*]pyridin‐5‐ones were excited using a 355 nm picosecond pulsed laser (PicoQuant LDH‐D‐C‐355) operating at a repetition rate of 10 MHz. The resulting fluorescence was collected through a 40× objective lens (Thorlabs LMM40XF‐P01) and detected by an avalanche photodiode (APD, PD‐050‐CTE). For each measurement, 20‐μL aliquots were taken from methanol, toluene, or acetonitrile solutions, which were prepared in situ immediately before analysis. Solid‐state samples were prepared by dissolving each one of the seven pyrrolo[3,4‐*b*]pyridin‐5‐ones in a blend of 50‐μL toluene and 150‐μL polystyrene (*η* = 3.5 cP, *ρ* = 880 kg/m^3^). The prepared solutions were processed into a solid polymer matrix via drop casting. Specifically, 60 μL of each solution was deposited onto 200‐μm‐thick coverslips (Pearl) and allowed to dry for 2 h. To preserve sample integrity, the final thickness of each film (39 ± 2 μm) was measured following the TCSPC experiments.

Time‐resolved fluorescence signals were measured for each pyrrolo[3,4‐*b*]pyridin‐5‐one thin film, including blanks containing only polystyrene films. No interference from the reflected beam was found on the coverslip surfaces or at the glass‐polymer interface for these TCSPC experiments. The temperature was maintained at 21°C throughout all time‐resolved experiments. To ensure a robust signal‐to‐noise ratio, the peak intensity for each decay was collected to 10,000 counts. The decay kinetics were analyzed using a nonlinear least‐squares reconvolution method of the Levenberg–Marquardt algorithm within the SymPhoTime 64 software. All data were accurately described by multiexponential fit models, yielding *χ* ^2^ values near unity in all instances. It should be noted that the %*Φ*_*f*_, as well as the absorption and emission spectra, were also measured for the pyrrolo[3,4‐*b*]pyridin‐5‐one samples in the polystyrene polymeric matrix using the instrumentation described above.

#### 2.2.4 Solid‐State Optical Properties

Solid‐state photoluminescence spectra were recorded using an Edinburgh Instruments FS5 spectrofluorometer equipped with a continuous‐wave 150‐W ozone‐free xenon arc lamp, Czerny–Turner monochromators, and an SC‐10 solid‐state sample holder. Fluorescence lifetimes were determined from the time‐resolved photoluminescence (TRPL) decay profiles, which were globally fitted using Fluoracle software (Edinburgh Instruments) with a multiexponential decay model (see Equation S1) through reconvolution analysis employing the instrument response function (IRF ~ 450 ps) measured under identical experimental conditions. TCSPC measurements were performed using a 375‐nm picosecond pulsed laser (EPL‐375, typical pulse width of 75 ps) as the excitation source for all compounds. Absolute solid‐state photoluminescence quantum yields (%*Φ*_*f*_) were measured at room temperature using the same instrument coupled to an SC‐30 integrating sphere equipped with a PTFE sample holder. Experimental details, fluorescence decay fitting parameters, and the corresponding solid‐state emission spectra are provided in the Supporting Information.

## 3 Results and Discussion

As the synthesized polyheterocyclic pyrrolo[3,4‐*b*]pyridin‐5‐ones **1a**–**g** brightened intensely when they were irradiated under UV light at 254 and 365 nm (see Figure 1), they could be potential luminescent materials. Thus, to rationalize the optical properties of pyrrolo[3,4‐*b*]pyridin‐5‐ones, a series of spectroscopic experiments and electronic structure calculations were performed accordingly.

**FIGURE 1:**
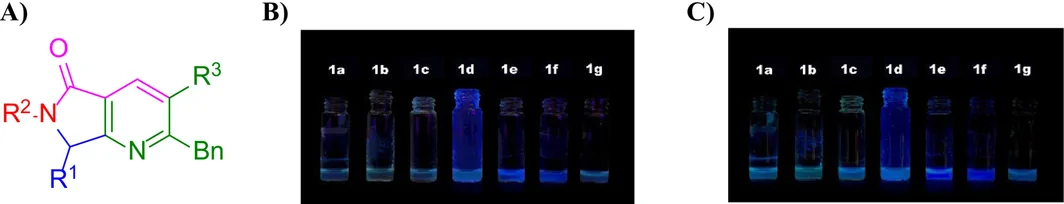
(A) Pyrrolo[3,4‐*b*]pyridin‐5‐one core; (B) naked‐eye‐visible emission of Compounds **1a–g** under UV illumination λ_exc_ = 254 nm in methanol. (C) As in (B), but with *λ*_*exc*_ = 365 nm.

Our research group previously reported the synthesis of a series of pyrrolo[3,4‐*b*]pyridin‐5‐ones with different substituents in key positions to understand the role of substitution pattern on the pyrrolo[3,4‐*b*]pyridin‐5‐one core. In 2024, two series of compounds were reported incorporating the BODIPY fragment (Table 1, Compounds **2a–g**) in the first one [27], the second one being based on a coumarin fragment (Table 1, Compounds **3a–g**) [28]. Later in 2025, a series of pyrazolyl‐pyrrolo[3,4‐*b*]pyridin‐5‐ones (Table 1, Compounds **4a–f**) was reported [14]. In the current work, the incorporation of the fragments of furan, tetrahydrofuran, thiophene, and pyridine was studied (Table 1, Compounds **1a–g**).

**TABLE 1:** Comparison between compounds previously reported and current compounds.

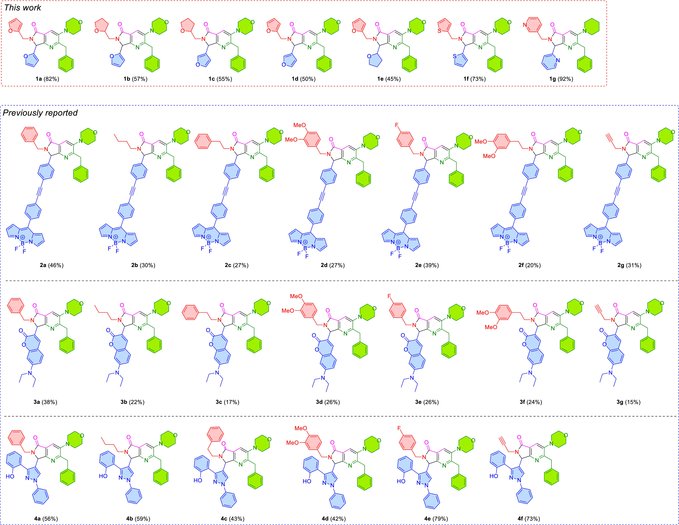					
Compound	Solvent	Absorption (nm)	Emission (nm)	Stokes shift (cm^−1^)	%*Φ*
**2a**	DMSO	506	528	823.5	0.02
**2b**	DMSO	505	530	934.1	0.26
**2c**	DMSO	505	531	969.6	17.15
**2d**	DMSO	505	531	969.6	0.42
**2e**	DMSO	510	528	668.4	0.06
**2f**	DMSO	506	528	823.5	—
**2g**	DMSO	506	530	894.9	—
**3a**	Tol Ace CHCl_3_	388 388 396	426 447 441	2299 3402 2577	— — 25.1
**3b**	Tol Ace CHCl_3_	389 388 398	429 446 442	2397 3352 2501	— — 20.0
**3c**	Tol Ace CHCl_3_	387 388 396	426 446 441	2366 3352 2577	— — 16.9
**3d**	Tol Ace CHCl_3_	385 386 396	427 445 442	2555 3435 2528	— — 20.2
**3e**	Tol Ace CHCl_3_	389 389 398	428 447 444	2342 3336 2603	— — 17.7
**3f**	Tol Ace CHCl_3_	388 388 397	426 445 443	2299 3301 2616	— — 19.0
**3g**	Tol Ace CHCl_3_	389 386 397	424 446 442	2122 3485 2564	— — 20.4
**4a**	ACN CHCl_3_ DMSO Solid	275 279 274 —	444 419 447 420	13,841.1 11,976.0 14,125.0 —	— 1.5 — 21.5
**4b**	ACN CHCl_3_ DMSO Solid	275 278 274 —	441 422 445 407	13,687.9 12,274.5 14,024.4 —	— 2.4 — 14.2
**4c**	ACN CHCl_3_ DMSO Solid	275 278 273 —	441 422 444 407	13,687.9 12,274.5 14,107.5 —	— 2.4 — 13.2
**4d**	ACN CHCl_3_ DMSO Solid	278 278 278 —	442 423 448 532	13,346.8 12,330.6 13,649.8 —	— 1.5 — 7.1
**4e**	ACN CHCl_3_ DMSO Solid	272 278 278 —	441 424 447 417	14,089.0 12,386.3 13,599.9 —	— 1.4 — 17.9
**4f**	ACN CHCl_3_ DMSO Solid	276 278 275 —	450 435 451 572	14,009.7 12,982.7 14,190.7 —	— 1.6 — 11.1

Abbreviations: Ace: acetone; ACN, acetonitrile; CHCl_3_, chloroform; DMSO, dimethyl sulfoxide; Tol, toluene.

The structural difference between the series is varied, which confers changes in the flexibility of the molecule that emits different wavelengths. For example, series that contain meso‐phenyl BODIPY, which has a moderate rotation fragment, emit around 528–531 nm. In this study, the changes in rotation in viscous media were observed. The series of the coumarin fragment (**3a–g**) contains a bulky fragment decreasing the emission (424–447 nm). Also, it incorporated a diethylamino rotation‐free fragment that promotes twisted intramolecular charge transfer (TICT). On the other hand, Compounds **4a–f** (pyrazolyl‐pyrrolo[3,4‐*b*]pyridin‐5‐ones) emit around 407–450 in solution and have better emission in the solid state (572 nm) due to the rigid system. In this study, the excited‐state intramolecular proton transfer (ESIPT) mechanism was observed.

Regarding excited‐state relaxation pathways, in the case of meso‐phenyl BODIPY‐pyrrolo[3,4‐*b*]pyridin‐5‐ones, low viscosity promotes nonradiative processes. Instead, in viscous media, it was restricted to rotation of the triple bond, increasing the fluorescence; as a result, it was observed to have low quantum yields in viscous media. The coumarin series indicates that rotation of the diethylamino fragment inhibits the fluorescence due to the TICT process, and the acceptor strength was reduced due to the bonding of the coumarin at the 3‐position to the pyrrolo[3,4‐*b*]pyridin‐5‐one core. In this case, chloroform (CHCl_3_) reduces the TICT process, increasing %*Φ*_*f*_ (17%–25%). Concerning the series of pyrazolyl‐pyrrolo[3,4‐*b*]pyridin‐5‐ones, the decay is related to the ESIPT process, reflected in the large Stokes shifts; proton transfer is unlikely in solution, as reflected in the low quantum yields and nonradiative pathways. In contrast, in the solid state, it promoted aggregation‐induced emission (AIE), increasing the %*Φ*_*f*_.

In the BODIPY series, it was found that one compound could act as a viscosity sensor. The 7‐(diethylamino)coumarin–pyrrolo[3,4‐*b*]pyridin‐5‐ones could be applied as biological markers due to their capability of increasing the fluorescence intensity. Finally, the pyrazolyl‐pyrrolo[3,4‐*b*]pyridin‐5‐ones could work as dual‐state emission luminogens (DSEgens).

This background information motivated us to continue investigating how substituents attached to the heterocyclic system affect its photophysical properties.

### 3.1 Spectral Characterization

The absorption and emission spectra of the synthesized compounds were measured in three different solvents: methanol, toluene, and acetonitrile. These solvents represent distinct chemical environments for the electronic states of the synthesized molecules and, therefore, serve to highlight differences in the measured optical properties. The spectral results for the seven Pyrrolo[3,4‐*b*]pyridin‐5‐ones are shown in Figures 2, S1, and S2 for methanol, toluene, and acetonitrile, respectively.

**FIGURE 2:**
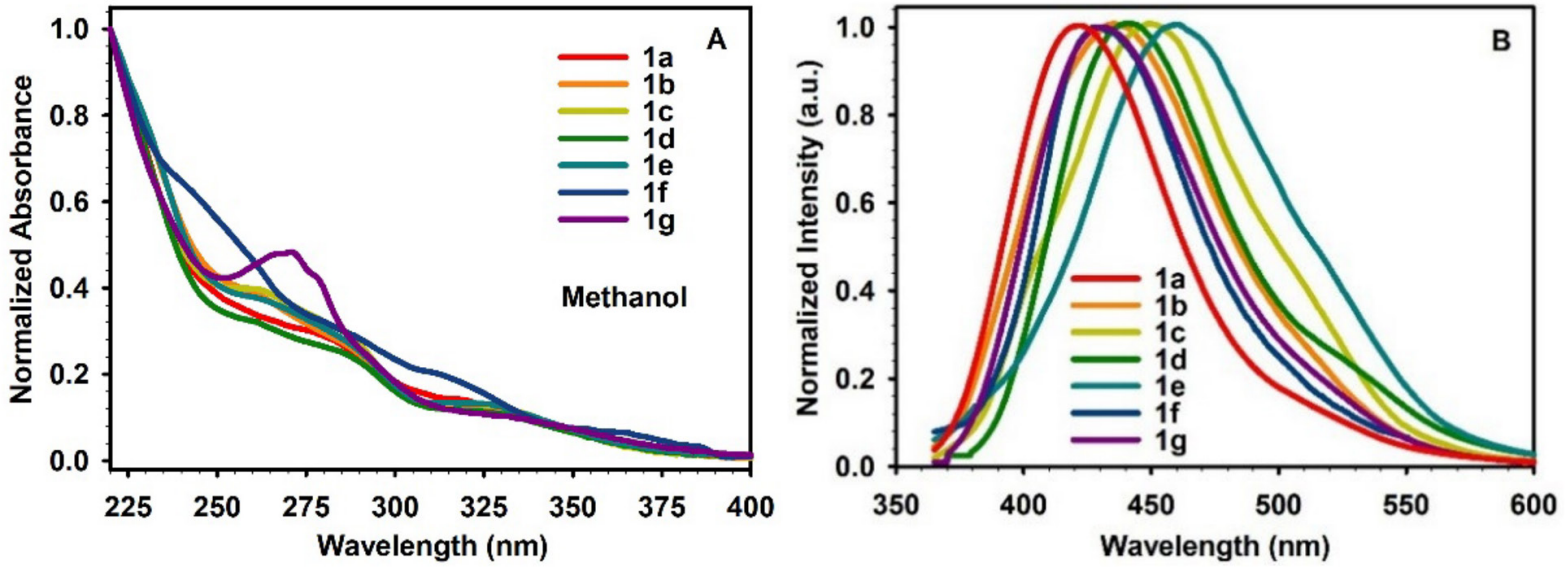
Steady‐state spectra for **1a–g** compounds in methanol. (A) Normalized absorption spectra at 220 nm. (B) Normalized fluorescence emission spectra at an excitation wavelength of 355 nm.

Based on the spectra, the absorption bands are located between 230 and 400 nm, displaying several signals that correspond to the electronic states of each pyrrolo[3,4‐*b*]pyridin‐5‐one. Upon excitation, all molecules emit photons with emission bands spanning the 370‐ to 600‐nm range. The emission maxima vary for each molecule, reflecting differences in solvent stabilization and Stokes shifts comparable to molecular vibrational energies. The individual Stokes shifts of the seven molecules ranged from 1810 to 4420 cm^−1^ in methanol, 2400 to 4670 cm^−1^ in acetonitrile, and 2560 to 4910 cm^−1^ in toluene. These variations corresponded to average values of 3384, 3501, and 3740 cm^−1^, respectively. These values represent a lower limit for each molecule; the actual shifts are likely larger, implying the presence of a charge‐transfer state responsible for this spectral separation. Because the absorption maximum of the emitting state significantly overlaps with adjacent electronic transitions, the onset of each spectrum was used to define the S_1_ absorption energy. Individual Stokes shift values, calculated from both the spectral onset and a tentative absorption maximum for this transition, are provided in Section 3.3 (see Table 3) and the Supporting Information (see Figure S3 and Table S1). Having molecules with large Stokes shifts is useful for microscopy or chemical environment sensing applications, as the emission signal remains free from probe reabsorption effects [29, 30, 31].

Because the absorption spectra in the three solvents are normalized in the ultraviolet (UV) electronic transition region, a direct comparison between the band intensities is enabled. The spectral profiles show that the signal intensity in toluene is higher than those recorded in methanol and acetonitrile. Based on experimental concentrations, optical path length, and absorbance (see Figure S3 and Table S2), the molar absorptivity coefficients (*ε*) were determined for each system. In methanol, acetonitrile, and toluene, the *ε* ranged from 1620 to 2400, 1560 to 5840, and 5700 to 14,240 M^−1^ cm^−1^, respectively.

This notable decrease in intensity within protic and polar media provides evidence of a hypochromic effect, which is attributable to the perturbative interaction of the solvent with the electronic density of the excited state; this phenomenon is significantly enhanced, increasing by more than a factor of five when transitioning from toluene to methanol. Furthermore, the absorption bands in methanol and acetonitrile exhibit a bathochromic shift (redshift) compared to toluene. Comparing the DFT calculation with the absorption band estimation using the spectral shoulder (see Figure S3), it can be seen in Table S1 that the calculations somewhat reproduce the position of this transition for the S_1_ state. This behavior (hypochromic and bathochromic) reinforces the hypothesis that the excited state possesses a pronounced charge‐transfer character, which is energetically stabilized by the increase in medium polarity [21, 32, 33].

### 3.2 Theoretical Calculations

As the first step, the theoretical calculations of the structures of compounds were optimized using methanol, toluene, and acetonitrile as solvents. In Table 2, frontier orbitals, contributions, oscillator strength, and wavelength of the absorption transitions in methanol are represented. As shown in Table 2, the S_1_ transition for all molecules is primarily characterized by the HOMO ➔ LUMO transition. The HOMO is predominantly distributed over the pyridine moiety of the pyrrolo[3,4‐*b*]pyridin‐5‐one core and the amine fragment (R^3^), which originates from the isocyanide component. In contrast, the LUMO is localized across the pyridine π‐system of the core and the adjacent methylene fragments. These orbital distributions indicate a subtle charge‐transfer contribution, shifting electron density from the HOMO, delocalized via conjugation with the R^3^ substituents, toward the core‐localized LUMO. Computational results confirm that the S_1_ state oscillator strengths (ranging from 0.05 to 0.08) for all derivatives correspond to symmetry‐allowed transitions in the UV region. This behavior could explain the hypothetical potential of all compounds to show optical properties, and hence, they could be used for further studies to test them as candidates for the fabrication of optical devices such as OLEDs [34]. In the remaining two solvents, the pyrrolo[3,4‐*b*]pyridin‐5‐ones exhibit similar behavior. Molecular orbital (MO) analysis clearly indicates that the S_0_–S_1_ transition is predominantly governed by the HOMO to LUMO transition, whereas the S_2_–S_4_ states involve significant MO mixing. Detailed computational results—including orbital energies, contour diagrams (isovalue = 0.02), oscillator strengths, and specific orbital contributions to the S_0_ to S_n_ transitions—are provided in Tables S3–S9. Furthermore, the theoretical UV–Vis spectra for all compounds, calculated at the B3LYP/6–31 + G(d) level of theory using geometries optimized in methanol, toluene, and acetonitrile, are shown in Figures S4–S6. In these figures, two distinct spectral bands were identified for all compounds: a primary, higher intensity band located between 270 and 310 nm and a secondary band between 340 and 450 nm. By correlating these computational results with steady‐state experimental data, it can be concluded that the transitions near 350 nm for all studied molecules are associated with the fluorescent S_1_ state, whereas the higher energy signals in the UV region correspond to the manifold of the S_2_–S_4_ electronic states.

**TABLE 2:** Data of the transitions for Compounds **1a–g** in methanol.

Molecule	Parameters	HOMO	LUMO
**1a**	S_0_ ➔ S_1_ *f* = 0.0757 *λ* = 341.08 nm H ➔ L 100%	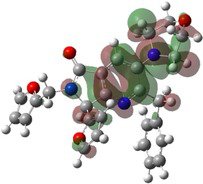	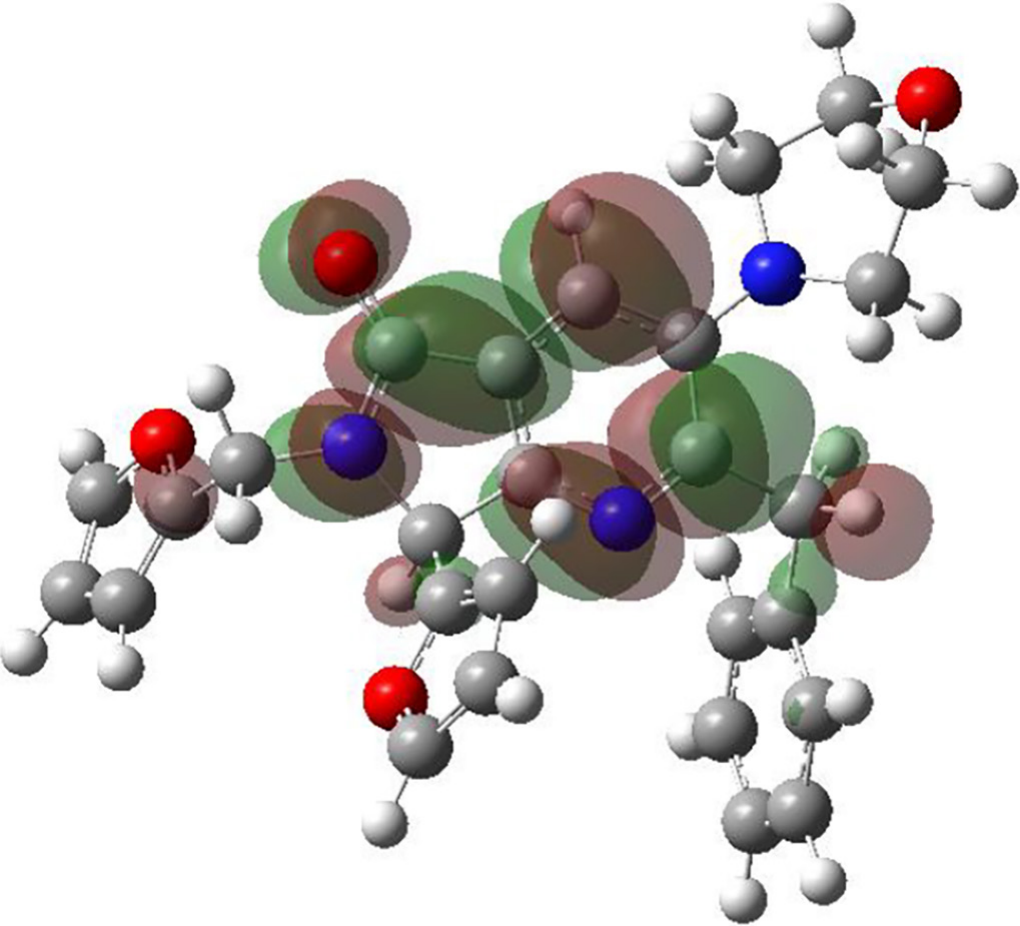
**1b**	S_0_ ➔ S_1_ *f* = 0.0788 *λ* = 336.84 nm H ➔ L 100%	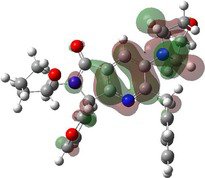	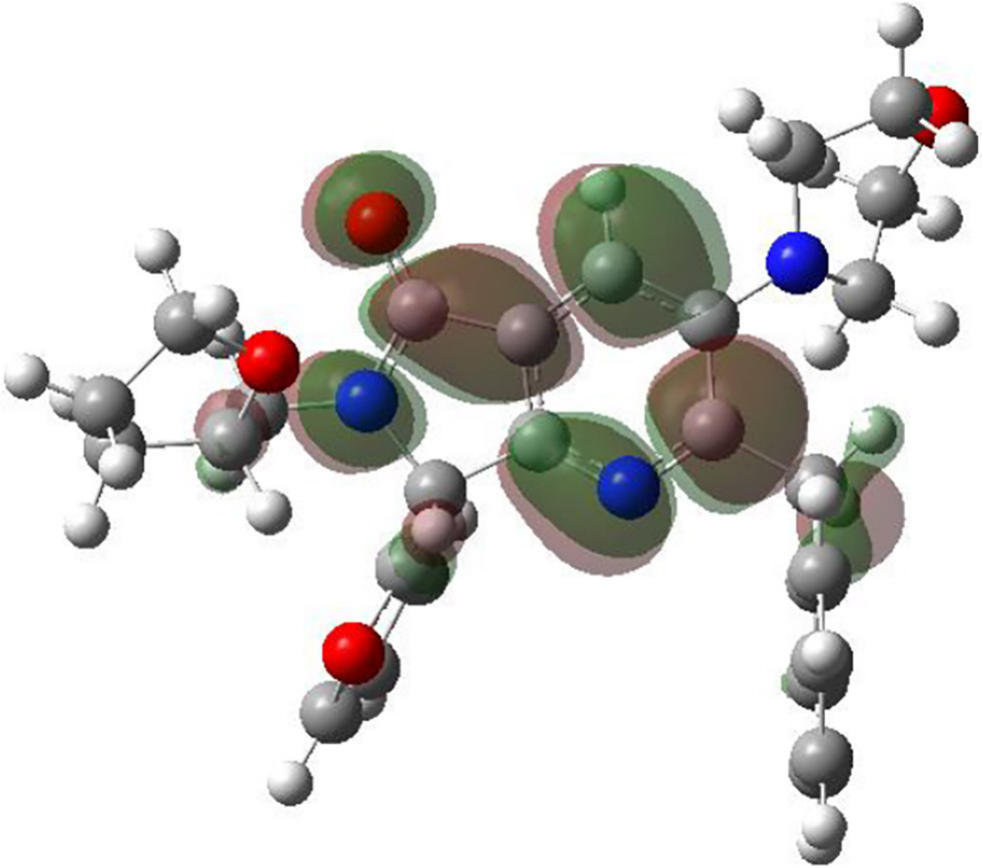
**1c**	S_0_ ➔ S_1_ *f* = 0.0781 *λ* = 336.16 nm H ➔ L 100%	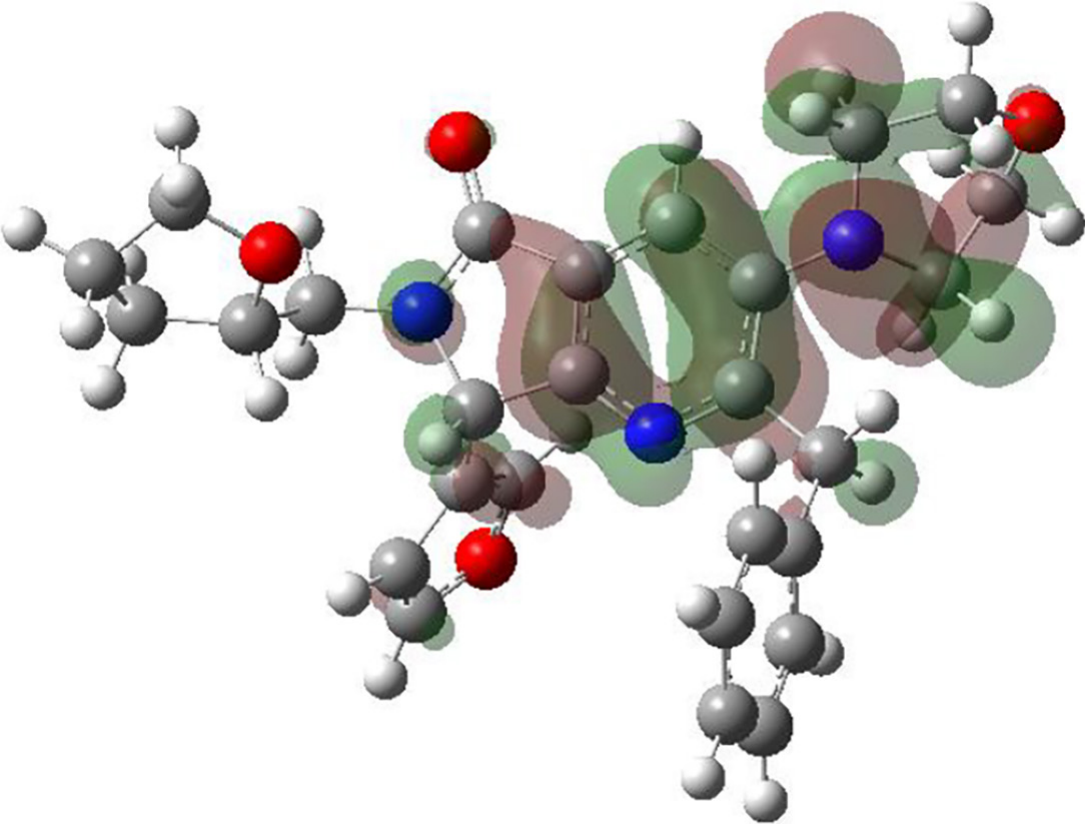	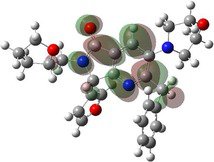
**1d**	S_0_ ➔ S_1_ *f* = 0.0531 *λ* = 355.27 nm H ➔ L 100%	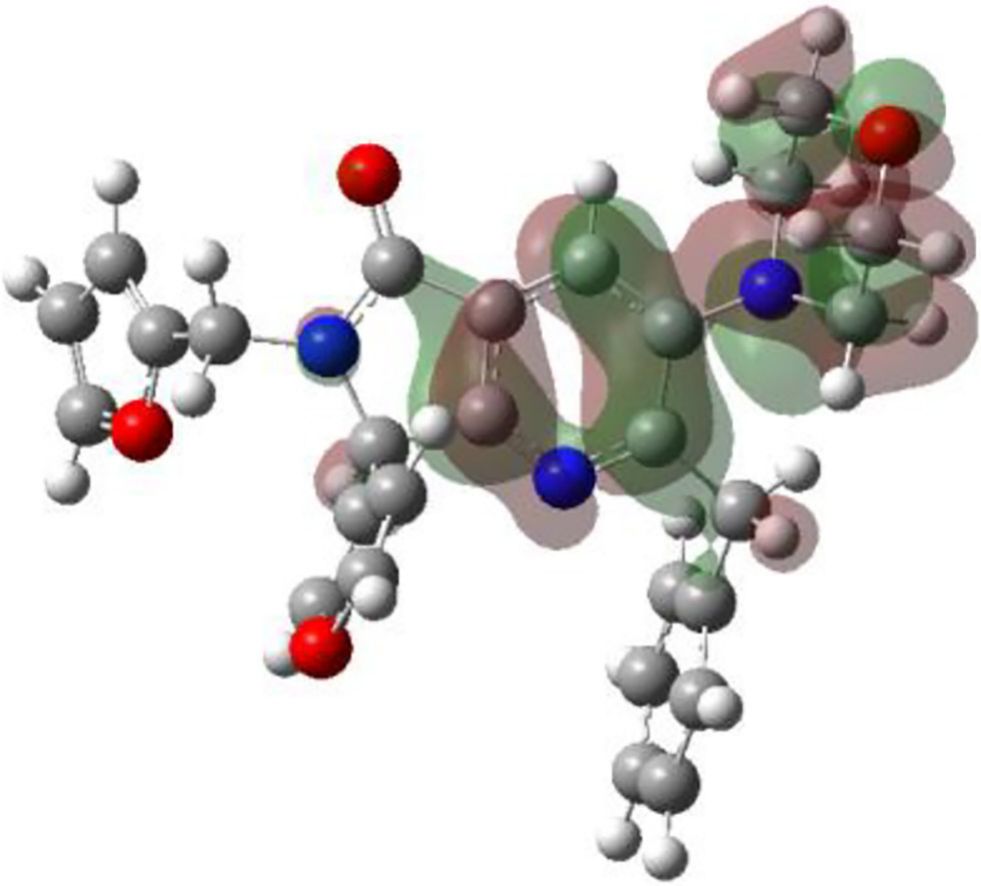	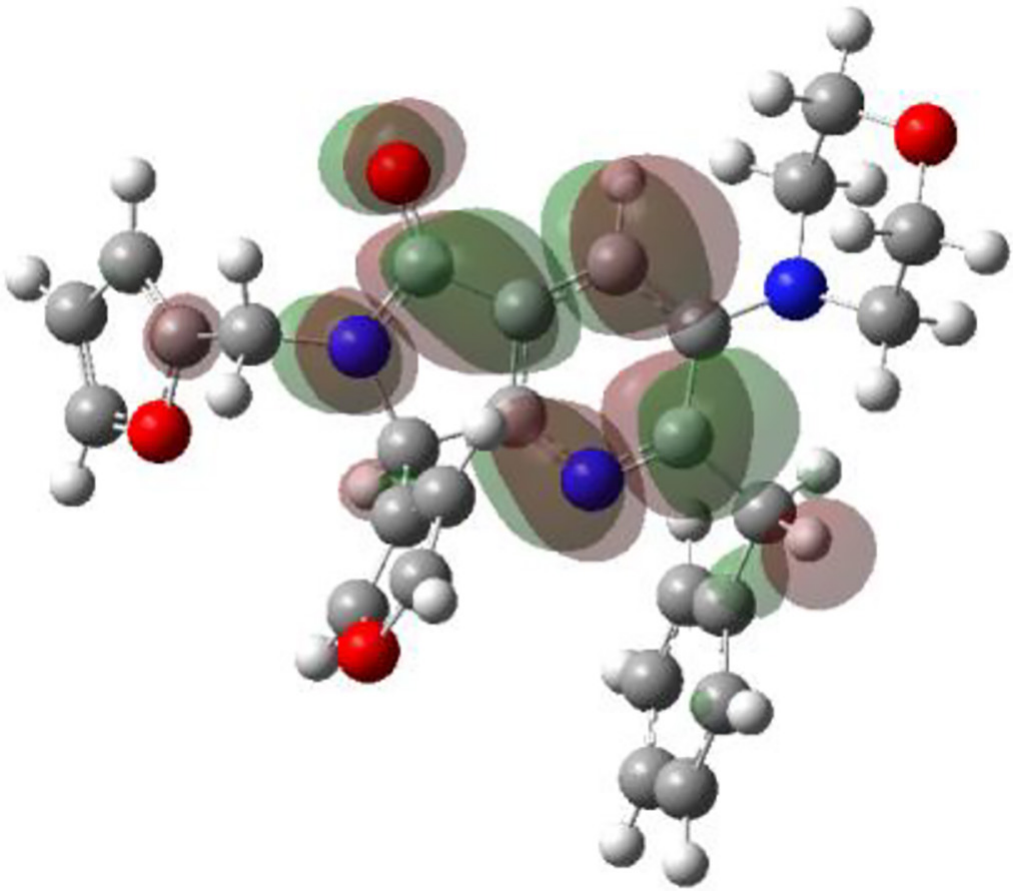
**1e**	S_0_ ➔ S_1_ *f* = 0.0516 *λ* = 353.07 nm H ➔ L 100%	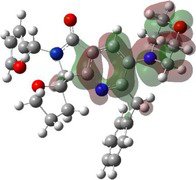	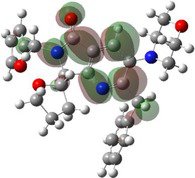
**1f**	S_0_ ➔ S_1_ *f* = 0.0506 *λ* = 357.66 nm H ➔ L 100%	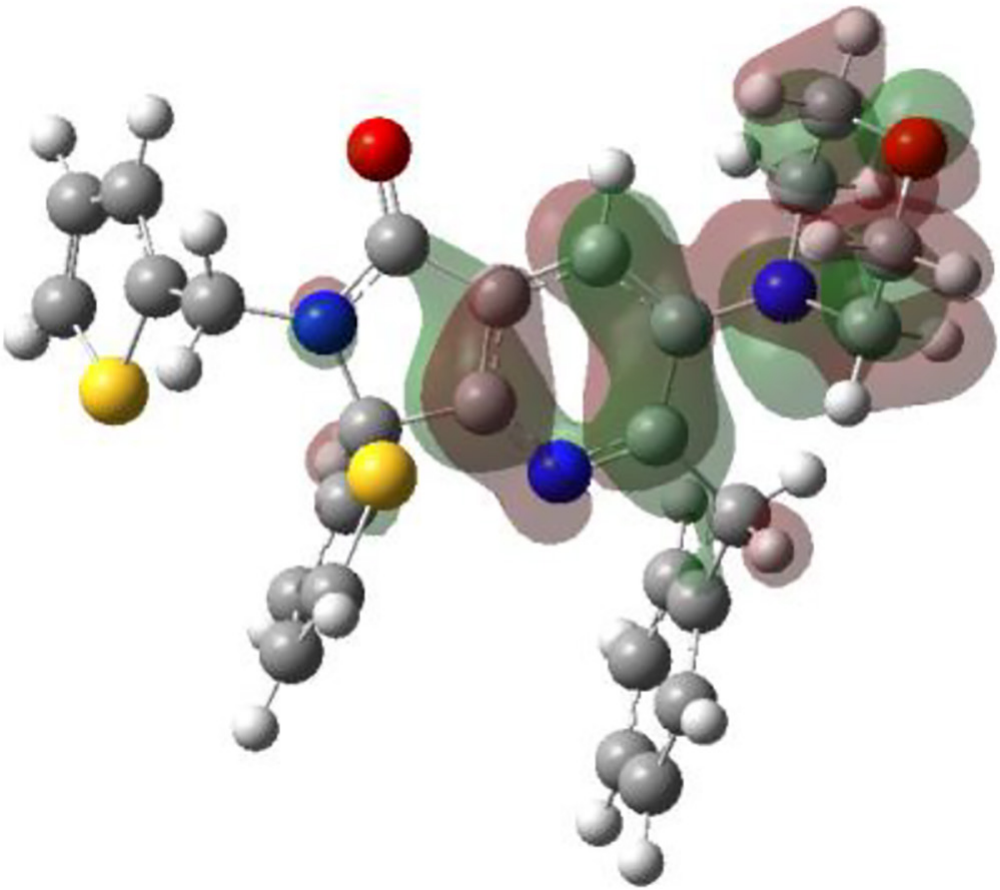	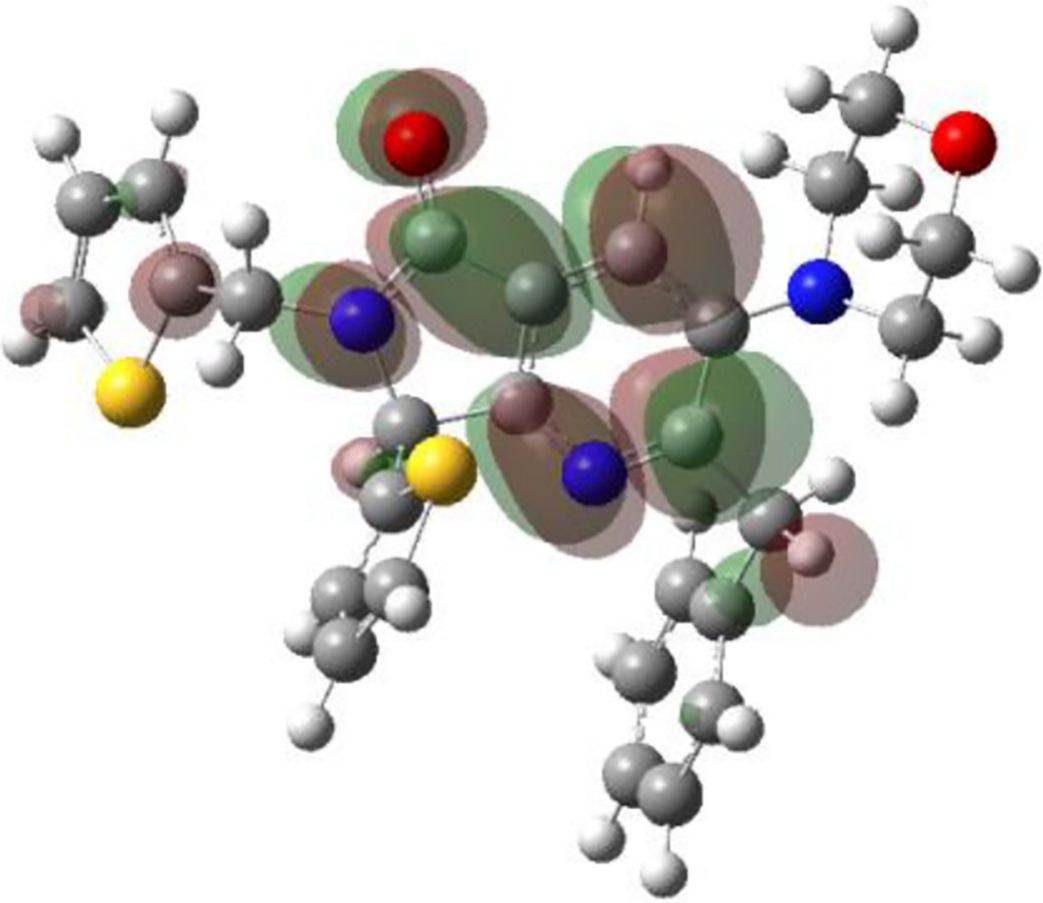
**1g**	S_0_ ➔ S_1_ *f* = 0.0594 *λ* = 336.82 nm H ➔ L 100%	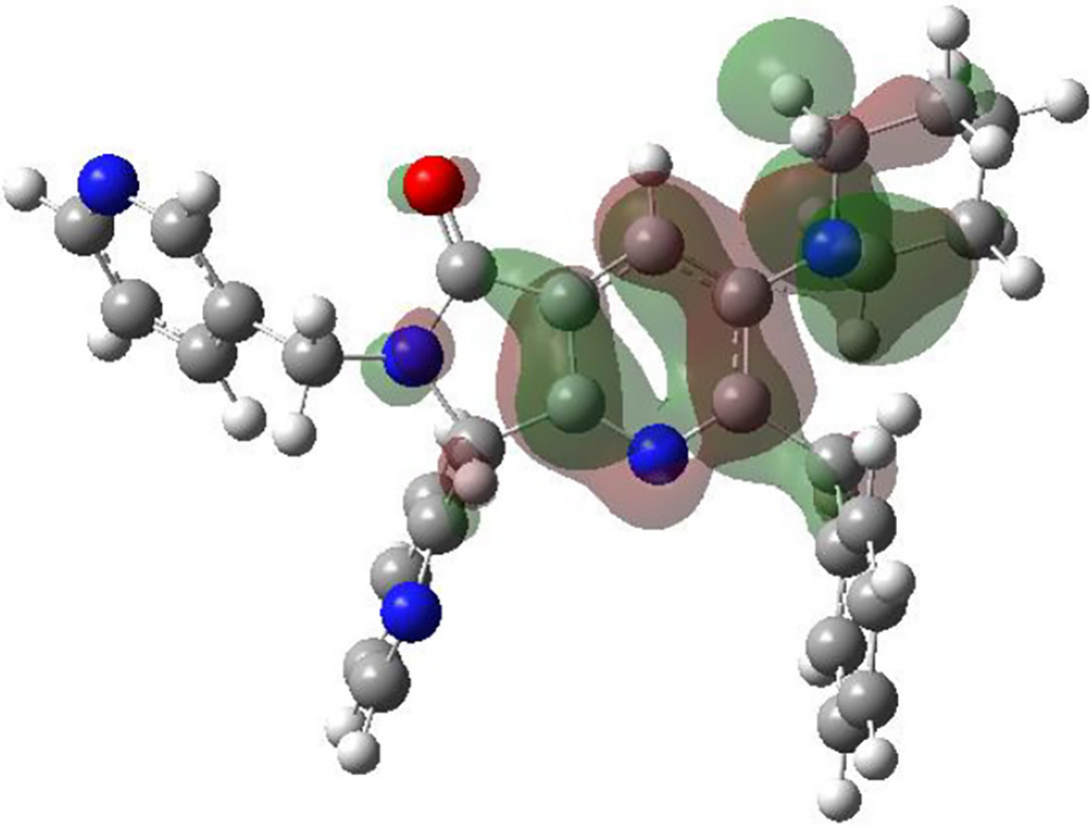	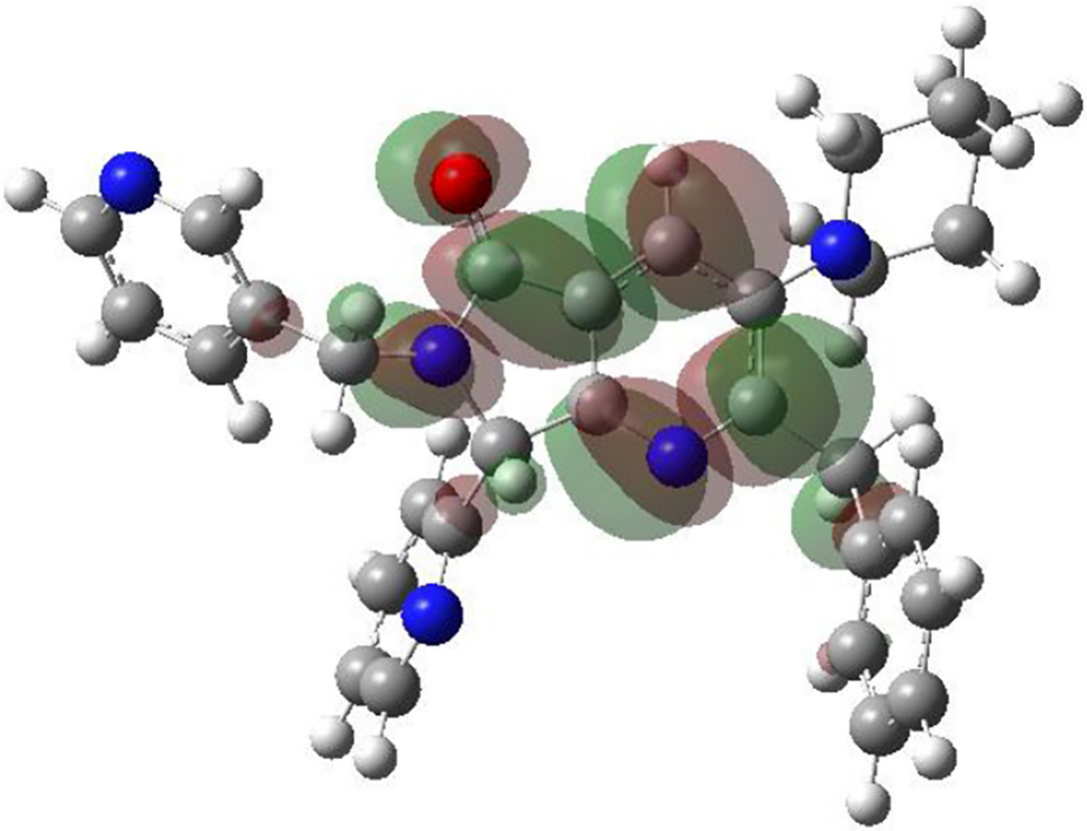

Tables S3–S9 show that the oscillator strength for S_1_ state decreases when transitioning from toluene to methanol and acetonitrile. Thus, the calculation in toluene consistently yields the highest magnitude, with values ranging from 0.0788 to 0.0506, whereas in methanol and acetonitrile, they range from 0.0716 to 0.0458 and from 0.0712 to 0.0460, respectively. This result is consistent with the spectral findings previously discussed. Regarding the S_n_ states, one state exhibits an oscillator strength ranging from 0.15 to 0.20, whereas others display magnitudes of barely 0.0015. An inspection of the MOs indicates that the R^1^ and R^2^ substituents of the pyrrolo[3,4‐*b*]pyridin‐5‐ones participate in these transitions. The MO contributions to these transitions toward higher lying states include at least five occupied orbitals lower in energy than the HOMO and three unoccupied orbitals higher in energy than the LUMO. The modification of substituents governs the transition to these electronic states, emerging as a key design variable for tuning the optical response of these systems.

### 3.3 Determination of Photoluminescence Quantum Yield and Fluorescence Lifetimes

In the emission spectra in methanol (Figure 2B), Compounds **1a–g** exhibit maxima ranging from 415 to 460 nm within the UV‐blue region. When switching to toluene as a solvent, a blue shift in the emission maxima is observed (390–435 nm). In contrast, stabilization in acetonitrile is similar to that in methanol; this behavior is expected for excited states with polar character [35, 36, 37, 38] and is generally reproduced by computational calculations (Tables 2, S1, and S3–S9). Furthermore, the emission spectra in toluene show more vibrational structure, suggesting fewer degrees of freedom available to dissipate the excitation energy from the fluorescence S_1_ state [39, 40, 41]. Based on the relative positions of the absorption and emission bands, fluorescence quantum yields in percentage (%*Φ*_*f*_) and fluorescence lifetimes were measured for all seven molecules across the three solvents. An excitation wavelength of 355 nm was chosen for the fluorescence experiments, and the signal was detected by collecting the emission across the entire spectral band. The results are summarized in Table 3. Using these spectral data and the average lifetimes (*τ*_*ave*_), the radiative (*k*_*r*_) and nonradiative rate constants (*k*_*nr*_) were calculated by the relationships *k*_*r*_ = (1 / *τ*_*ave*_) (%*Φ*_*f*_ / 100), and *k*_*nr*_ = (1 / *τ*_*ave*_) (1 – %*Φ*_*f*_ / 100), respectively [40, 41]. *k*_*r*_ and *k*_*nr*_ rates provide information on how the decay processes are influenced by the substituents at positions R^1^, R^2^, and R^3^.

**TABLE 3:** Photophysical properties of the pyrrolo[3,4‐*b*]pyridin‐5‐ones. The solvent abbreviations are MeOH for methanol, ACN for acetonitrile, Tol for toluene, and PS for the polystyrene matrix.

Comp.	Solv.	%*Φ*_*f*_	${\bar{\nu }}_{\text{onset}}$ (cm^−1^)	${\bar{\nu }}_{\text{fluo}}$ (cm^−1^)	Stokes shift ${\bar{\nu }}_{\text{onset}}$− ${\bar{\nu }}_{\text{fluo}}$ (cm^−1^)	*τ*_*ave*_ (ns)	*k*_*nr*_/10^8^ (s^−1^)	*k*_*r*_/10^8^ (s^−1^)
**1a**	MeOH ACN Tol PS	12.07 33.48 24.83 29.54	25,510 27,750 28,170 26,320	23,700 22,880 23,260 21,830	1810 4670 4910 4490	3.4 10.9 5.6 9.5	2.61 0.61 1.34 0.74	0.16 0.45 0.33 0.31
**1b**	MeOH ACN Tol PS	13.10 10.74 10.81 15.57	27,100 27,030 27,400 26,110	22,940 24,630 22,880 20,370	4160 2400 4520 5740	5.1 9.4 4.7 7.2	1.72 0.95 1.89 1.17	0.17 0.14 0.14 0.22
**1c**	MeOH ACN Tol PS	17.02 28.10 11.21 16.72	26,460 27,550 28,090 25,770	22,270 24,330 24,940 22,030	4190 3220 3150 3470	8.1 9.5 5.5 8.9	1.03 0.76 1.61 0.94	0.23 0.37 0.15 0.19
**1d**	MeOH ACN Tol PS	15.48 14.32 14.36 13.43	25,840 26,110 27,620 24,450	22,620 23,580 25,060 21,320	3220 2530 2560 3130	7.8 9.3 5.7 7.2	1.08 0.92 1.51 1.20	0.21 0.19 0.19 0.19
**1e**	MeOH ACN Tol PS	16.09 45.53 15.27 12.87	26,110 27,100 28,250 26,950	21,690 22,940 24,880 21,880	4420 4160 3370 5070	8.4 11.6 5.1 7.9	1.00 0.47 1.67 1.10	0.21 0.61 0.20 0.16
**1f**	MeOH ACN Tol PS	9.22 21.88 6.93 8.23	25,770 26,180 27,030 24,390	23,360 22,680 23,870 22,370	2410 3500 3160 2020	6.8 12.2 5.2 8.5	1.33 0.64 1.80 1.08	0.12 0.29 0.09 0.10
**1g**	MeOH ACN Tol PS	13.08 31.53 17.63 22.26	26,740 26,600 27,930 26,530	23,260 22,570 23,420 22,880	3480 4030 4510 3650	4.9 9.5 5.8 8.8	1.77 0.72 1.43 0.88	0.17 0.42 0.24 0.25

An analysis of the excited‐state lifetimes of molecules **1a–g**, which have furan, tetrahydrofuran, thiophen, and pyridine as substituents at R^1^ and R^2^, indicates that, despite the structural similarity between these seven molecules, their relaxation dynamics are markedly different (see Table 3). The first five molecules (**1a–e**), which contain a morpholine group at *R* ^3^ and both tetrahydrofuran and furan rings at R^2^ and R^1^, exhibit average lifetimes ranging from 3.4 to 8.4 ns in methanol; these values increase to a range of 9.3–11.6 ns in an acetonitrile environment. In contrast, in toluene, the lifetimes remain consistently around 5.4 ns for all molecules, falling between 5.1 and 5.7 ns. This difference is most evident when comparing the radiative (*k*_*r*_) and nonradiative (*k*_*nr*_) decay constants, as well as the average individual lifetimes shown in Table 3. The measured decay curves, the pre‐exponential factors, and the lifetime magnitudes are provided in the Supporting Information (pp. S50–S52).

To investigate the effect of substituent modification on the photophysical properties of this set of molecules, derivatives **1f** and **1g**, which contain substituents other than morpholine, tetrahydrofuran, and furan, were also analyzed. Thiophene and pyridine were selected based on their distinct effects on basicity, steric bulk (substituent volume), and the varying electronegativity of the heteroatoms relative to oxygen. The fluorescence lifetimes and quantum yields of these molecules were also measured in the same three solvents described previously.

Figure 3 illustrates the impact of structural substitutions on the pyrrolo[3,4‐*b*]pyridine‐5‐one core and their subsequent effect on excited‐state lifetimes (*τ*_*ave*_) and fluorescence quantum yields (*Φ*_*f*_) (see Equation S2). These substituents modulate the photophysical response through three primary mechanisms: electronic donation (inductive/mesomeric effects), hydrogen‐bonding capacity (mediated by oxygen‐containing moieties), and the steric bulk of the substituent [42, 43, 44, 45]. This volume determines the rotational friction of the molecule, which varies significantly depending on the viscosity and chemical nature of the surrounding medium (acetonitrile, methanol, or toluene). Consequently, these substituents impart structural flexibility, allowing the investigated molecules to function as molecular rotors (rotameters) [42, 43, 44, 45]. Although the specific nonradiative decay channel responsible for the deactivation of the S_1_ excited state (populated via 355‐nm irradiation) was not explicitly identified in this study, the results suggest a deactivation mechanism governed by intramolecular rotation and a potential transition from the Franck‐Condon state (LE) to a relaxed charge transfer state (CT) [46, 47, 48, 49].

**FIGURE 3:**
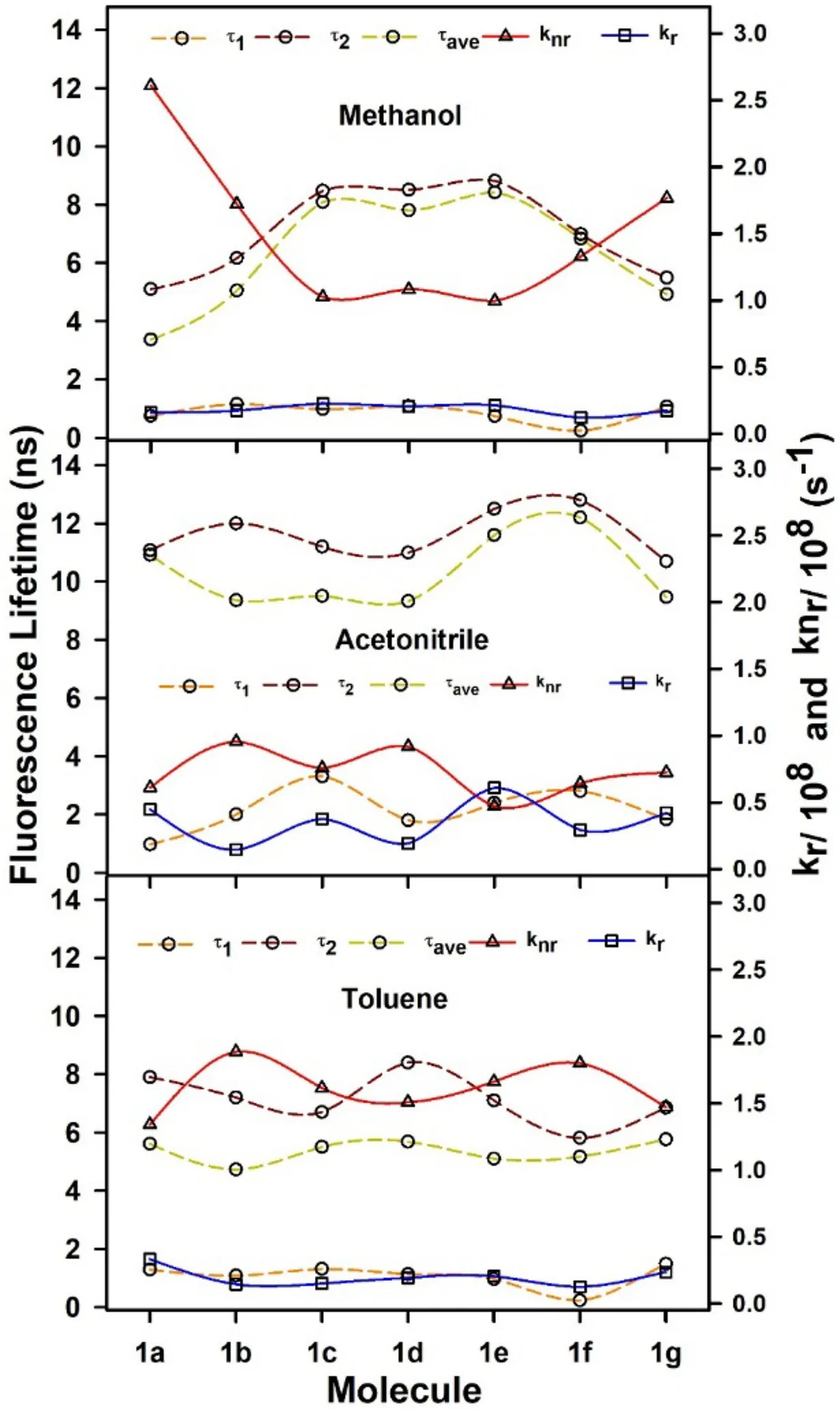
Rate constants and lifetimes for the pyrrolo[3,4‐*b*]pyridin‐5‐ones. The magnitudes of the radiative (blue line and squares) and nonradiative (red line and triangles) rate constants are shown with solid lines. The lifetimes for each molecule are shown with dotted lines (circles). The fits were performed using biexponential functions. Toluene (bottom), acetonitrile (middle), and methanol (top).

When inspecting the individual time components, *τ*_1_ and *τ*_2_, for the seven molecules shown in Figure 3 and Tables S9–S11, it is observed that their magnitudes change drastically due to the effects of excited‐state interactions with the three solvents. Conveniently, the plots in Figure 3 share the same scale for the vertical axes, thereby allowing a direct comparison. The times *τ*_1_ and *τ*_2_ can be associated with the LE (locally excited) state and the relaxed charge‐transfer (CT) state, respectively; thus, the short *τ*_1_ constant indicates the rapid loss of population from the LE state following the vertical transition. This relaxation process occurs on timescales close to 1 ns, with average magnitudes of 1.2 ± 0.2, 2.1 ± 0.3, and 1.0 ± 0.2 ns in toluene, acetonitrile, and methanol, respectively. Interestingly, molecule **1f** exhibits the shortest τ_1_, lasting just 0.3 ns in toluene and methanol. This implies that additional deactivation channels have emerged due to the effect of the sulfur heteroatom, possibly via an ISC (intersystem crossing). This lifetime slows down when molecule **1f** is dissolved in acetonitrile. By comparing the modulation of **1a–1g** lifetimes, which doubles when transitioning from toluene to acetonitrile, it can be proposed that this change arises from the existence of an energy barrier for the LE → CT crossing. This barrier is higher in acetonitrile because it depends on the solvent reorganization around the CT relaxed excited state, whereas it is lower in toluene because, being nonpolar, it does not require energy expenditure for reorganization. Consequently, in toluene the LE state leaves the FC (Franck‐Condon) region at a faster rate [38, 47]. In methanol, both time constants (*τ*_1_ and *τ*_2_) exhibit a nonsystematic effect—becoming either faster or slower relative to toluene—which is likely driven by hydrogen‐bonding interactions that alter the deactivation dynamics along a torsional coordinate. This effect impacts the transition from the LE to the CT state, as well as photon emission and nonradiative decay pathways [41, 42].

Analysis of $\tau_{2}$ reveals that the charge‐transfer (CT) state generated from the Franck–Condon (FC) region exhibits an average lifetime of 7.1 ± 0.2 ns (averaged across toluene and methanol), corresponding to a radiative rate constant ($k_{r}$) of 1.4 × 10^8^ s^−1^. It is evident that for each molecule, this *τ*_2_ value is also modulated as a function of solvent polarity and the magnitude of the dipole moment of the relaxed CT excited state. The specific lifetimes of each molecule will be highly useful for utilizing these compounds in molecular environment sensing applications, where monitoring the effects of solvent polarity, viscosity, or protic character on the intensity‐weighted average lifetimes is required (see intensity‐weighted average lifetime in Section S4.2).

This research explores the utility of pyrrolo[3,4‐*b*]pyridin‐5‐ones as molecular rotors for probing local viscosity and dielectric environments. By utilizing TCSPC fluorescence data, the photophysical behavior of these seven derivatives was characterized. Specifically, the ratio of average lifetimes (*τ*_*ave*_) across diverse solvents serves as a fundamental metric for analyzing environmental sensitivity and the competition between radiative and nonradiative processes. Table 4 shows the lifetime ratios for the seven molecules. The methanol/acetonitrile ratio monitors the ability of the pyrrolo[3,4‐*b*]pyridin‐5‐ones to form hydrogen bonds with the alcohol. Because both solvents have similar dielectric constants (dielectric constant [*ε*_*r*_] methanol: 33; acetonitrile: 37), the polarity effect is expected to be negligible. The data provided highlights a clear and consistent trend across all seven molecular rotors: Fluorescence lifetimes (*τ*_*ave*_) are significantly longer in acetonitrile compared to methanol. This behavior is characteristic of molecular rotors, where the excited‐state dynamics are highly influenced by the solvent's ability to facilitate or hinder internal rotations and charge transfer. The lifetimes in acetonitrile range from 9.3 to 12.2 ns. Acetonitrile is a polar aprotic solvent. Although it is polar, it lacks the ability to form strong hydrogen bonds with the solute. This results in less stabilization of the nonradiative quenched states, allowing the molecule to remain in the excited state longer. Conversely, in methanol, lifetimes drop dramatically, ranging from 3.4 to 8.4 ns. Methanol is a polar protic solvent. It can form hydrogen bonds, which often facilitate faster nonradiative decay pathways, such as internal conversion (IC) or stabilized CT (Not verified in this study). These seven molecules were categorized based on how drastically their dynamics change between the two polar solvents.

**TABLE 4:** Ratio between the fluorescence lifetimes of seven pyrrolo[3,4‐*b*]pyridin‐5‐ones **1a–g** depending on the chemical environment.

Comp.	Hydrogen bonding	Polarity‐viscocity	Polarity‐hydrogen bonding	Mechanical
	Ratio (ACN/MeOH)	Ratio (ACN/Tol)	Ratio (Tol/MeOH)	Ratio (polymer/tol)
**1a**	3.24	1.95	1.66	1.70
**1b**	1.85	1.98	0.94	1.53
**1c**	1.18	1.73	0.68	1.62
**1d**	1.19	1.64	0.73	1.26
**1e**	1.38	2.28	0.60	1.55
**1f**	1.79	2.36	0.76	1.63
**1g**	1.92	1.64	1.17	1.52

Table 4 shows that **1a**, **1g**, and **1b** are the molecules with the greatest decrease in lifetimes (ratios > 1.85). This indicates that their excited‐state decay is highly coupled to hydrogen‐bonding interactions. The presence of the hydroxyl group in methanol significantly accelerates their nonradiative relaxation [50, 51, 52]. These would be the best candidates for sensing proticity [53] or water contamination in organic solvents. Compounds **1c** and **1d** show ratios near 1.2 times; these molecules are relatively blind to the difference between acetonitrile and methanol. Their lifetimes remain high (around 8–9 ns) regardless of the solvent's ability to hydrogen bond. This suggests their structural geometry might be more rigid or their electronic state is less influenced by the solvent's protic nature.

To characterize the excited‐state dynamics of these seven molecular rotors, we evaluated the impact of solvent polarity shifts‐moving from a nonpolar environment (toluene, *ε*_*r*_ = 2.4) to a highly polar aprotic environment (acetonitrile, *ε*_*r*_ = 37) [54] This dielectric transition was analyzed alongside variations in local viscosity, ranging from 3.6 to 5.9 cP [54] to decouple electronic stabilization from mechanical restriction. By maintaining these specific conditions, we can isolate how the stabilization of the charge transfer (CT) state and the energy barrier for intramolecular rotation dictate the observed fluorescence lifetimes (*τ*). Unlike the previous comparison with methanol, where hydrogen bonding was the dominant factor, this analysis focuses mostly on solvent polarity and the stabilization of the excited state. Although the viscosity is not identical for both solvents, the changes in shelf life are expected to be primarily due to the electronic interaction between the molecule and the dielectric environment of the solvent.

In Table 4, Molecules **1f** and **1e** show the highest sensitivity to polarity. Their lifetimes are more than double when moving from a nonpolar (Tol) to a polar environment (ACN). This suggests these rotors have a large change in dipole moment upon excitation. This reduction can also be reinforced by the greater viscosity of toluene compared to acetonitrile. These are excellent candidates for probes designed to detect local dielectric shifts, such as those occurring during protein folding or lipid membrane phase transitions, with the advantage of the morpholine group, which has an affinity for biological tissue. Interestingly, the **1d** and **1g** molecules can be used as the best control, as they remain more consistent across different polarities.

In many molecular rotors, high polarity stabilizes an intramolecular charge transfer (ICT) state, which usually acts as a quenching pathway [21, 40, 41, 42, 55, 56]. However, for pyrrolo[3,4‐*b*]pyridin‐5‐ones, the lifetimes increase with polarity. This suggests that for these specific molecules, the high dielectric constant of acetonitrile stabilizes the bright (emissive) state more effectively than the dark charge transfer state, or it increases the energy barrier required to reach the quenching geometry (through molecular rotations of the substituents) [57]. This barrier is also related to the rotational ability of R^1^, R^2^, and R^3^, which is more impeded in toluene than in acetonitrile because the viscosity is twice as large. This can be determined using the up‐conversion technique, because the TCSP‐IRF used in this study was > 200 ps (see Figure S7).

The fourth column of Table 4 compares the *τ*_*ave*_ values measured in toluene with those measured in methanol. The comparison between methanol and toluene is particularly interesting because it contrasts a polar protic solvent with a nonpolar aprotic solvent. Although their viscosities in your study are identical (5.9 cP), the chemical interactions they have with your molecular rotors are fundamentally different. For these molecules, the lifetime is significantly shorter in methanol than in toluene. For Molecules **1a** and **1g**, extinction by methanol is observed. Because methanol is protic, it can form hydrogen bonds with the functional groups of the rotor. This often opens up vibrational nonradiative pathways, as can be clearly seen in the plots above and below in Figure 3, allowing the excited state to dissipate energy as heat rather than light. Even though methanol is more polar, the specific quenching effect of the hydroxyl (‐OH) group outweighs any dielectric stabilization. Conversely, Molecules **1e**, **1c**, **1d**, and **1f** show an inverted trend. For these specific structures, the high polarity of methanol (*ε*_*r*_ = 33) may stabilize the emissive state so effectively that it overcomes the hydrogen‐bonding quenching effect. These molecules might be structurally designed such that the rotor parts are shielded from hydrogen‐bond‐induced quenching, allowing the simple polarity effect to dominate.

To measure the effect of the restriction on the rotation of substitutes, experiments were conducted in a polystyrene matrix. The data were analyzed in relation to toluene. Table 4 shows the ratio between these two solid–liquid environments. Data on lifetime, pre‐exponential factors, fluorescence yield, rate and Stokes Shift are shown in the Supporting Information (Tables S11, S12, and S14). Comparing the pyrrolo[3,4‐*b*]pyridin‐5‐ones lifetimes in polystyrene versus toluene allows us to isolate the effect of viscosity and molecular confinement, as both environments are nonpolar hydrocarbons with similar chemical interactions. In all cases, the incorporation of the molecules into the polystyrene matrix increases the lifetime of the fluorescence (*τ*_*ave*_).

The most critical change is the suppression of nonradiative rotation. In liquid toluene, the rotors can easily twist into a dark state (CT), which dissipates energy without emitting light, resulting in shorter lifetimes. In the solid Polystyrene matrix, the free volume is much smaller. The mechanical rotation is physically hindered by the polymer chains, forcing the molecule to stay in its radiative state longer. With a density of 880 kg/m^3^ and an initial viscosity of 3.5 cP, the polystyrene solution creates a crowded environment. As the solvent evaporates, the viscosity increases by several orders of magnitude, reaching 10^3^–10^5^ times its initial value [58, 59]. This confinement can prevent the molecule from reaching the specific planar or twisted geometry required for internal conversion (quenching). As detailed in Table S12, the long lifetime components are close to 12 ns within the polymer matrix, whereas the shorter components reach up to 1.5 ns, remaining similar to those observed in toluene. Molecules **1a** and **1f** nearly double their fluorescence lifetimes in the solid matrix, suggesting that they act as highly active molecular rotors with a low rotational barrier in solution. The data in Table 3 clearly show that the *k*_*nr*_ for all pyrrolo[3,4‐*b*]pyridin‐5‐ones in a polystyrene matrix are roughly 60% lower than those in toluene and comparable in magnitude to those in ACN. Overall, the *k*_*r*_ values across these solvents and the polystyrene matrix remain virtually unchanged, indicating that this represents the relaxation rate constant of the emissive state in the absence of competing deactivation pathways. The polymeric matrix effectively inhibits this motion, possibly due to interactions involving the higher electron density of the furan and thiophene substituents in these molecules.

Molecules **1b**, **1c**, **1e**, and **1g** show less change. Molecule **1d** shows the smallest change (only 26% increase). This indicates that its rotation was likely already somewhat restricted or that its decay mechanism is less dependent on large‐scale conformational changes compared to the others. In general, the shift from toluene to polystyrene acts as a mechanical stabilizer. By increasing the viscosity from a liquid to a solid‐like state, we have effectively turned off the primary quenching mechanism for these rotors.

### 3.4 Solid‐State Photophysical Properties

To further evaluate the photophysical behavior of the pyrrolo[3,4‐*b*]pyridin‐5‐ones **1a–g** under restricted molecular motion, their solid‐state optical properties were investigated. The normalized solid‐state photoluminescence emission spectra together with the corresponding time‐correlated single‐photon counting (TCSPC) decay profiles are shown in Figure 4, whereas the solid‐state photoluminescence quantum yields (%*Φ*_*f*_) and average fluorescence lifetimes are summarized in Table 5. Fluorescence spectra were recorded using an excitation wavelength of 400 nm for Compounds **1a**, **1b**, **1c**, and **1g**, whereas Compounds **1d**, **1e**, and **1f** were excited at 350 nm according to their absorption characteristics. Complete lifetime fitting parameters and %*Φ*_*f*_ are provided in the Supporting Information (Table S13 and Figures S8–S14).

**FIGURE 4:**
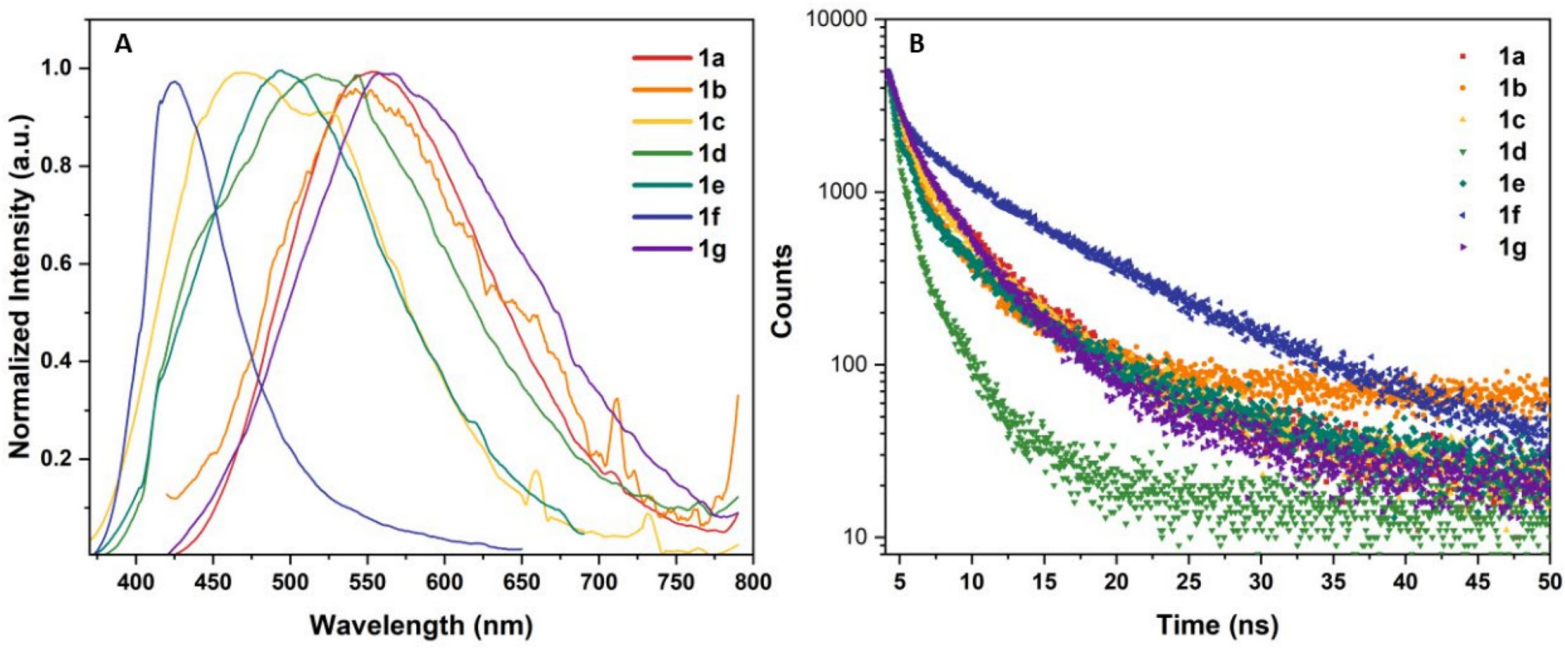
(A) Solid‐state photoluminescence emission spectra recorded at *λ*_*ex*_ = 400 nm for Compounds **1a**, **1b**, **1c**, and **1g**, and at *λ*_*ex*_ = 350 nm for **1d**, **1e**, and **1f**. (B) Fluorescence decay profiles in the solid state were obtained by TCSPC using a picosecond pulsed laser with *λ*_*ex*_ = 375 nm for Compounds **1a–g**.

**TABLE 5:** Solid‐state photophysical properties of Compounds **1a–g**.

Compound	*λ*_*det*_ (nm)^a^	%*Φ*_*f*_	*τ*_*ave*_ (ns)	*k*_*nr*_/10^8^ (s^−1^)	*k*_*r*_/10^8^ (s^−1^)
**1a**	550	4.70	6.0	1.59	0.078
**1b**	520	3.42	5.5	1.76	0.062
**1c**	520	2.12	6.0	1.63	0.035
**1d**	520	1.41	2.5	3.94	0.056
**1e**	500	6.75	6.3	1.48	0.107
**1f**	425	10.22	9.0	1.00	0.114
**1g**	550	1.16	5.5	2.02	0.021

^a^ The *λ*_*det*_ values for all compounds correspond to the emission maxima at which the decay profiles were recorded.

As shown in Figure 4A, all compounds remain emissive in the condensed phase, although their emission maxima are strongly influenced by the substitution pattern. As summarized in Table 5, the maximum solid‐state emission spans from 425 to 550 nm, demonstrating that relatively subtle structural modifications effectively tune the emission over a broad spectral window. These findings offer an interesting contrast to the fluorescence spectra recorded in both the liquid solvents and the polymer matrix (see Figures 2B, S1B, S2B, and S15). The emission maxima span 390–435 nm in Tol, 410–450 nm in ACN, 415–460 nm in MeOH, and 435–495 nm in PS. In solid measurement, Compounds **1a** and **1g** exhibit the most red‐shifted emission centered at 550 nm, whereas Compounds **1b–d** emit at 520 nm, and Compound **1e** displays a slightly red‐shifted emission at 500 nm. Notably, Compound **1f** exhibits an emission centered at 425 nm, indicating that the thiophene substituent does not modify the excited‐state electronic structure relative to the other analogues. Regarding the emission bands measured in the solid state, this broad spectral tunability highlights the strong influence of the substitution pattern on the photophysical properties of the pyrrolo[3,4‐*b*]pyridin‐5‐one scaffold. The spectral changes (redshift and broadening) and the emergence of an additional lifetime component (see below) indicate the presence of new deactivation channels in the solid state (see Equation S3).

Fluorescence decay profiles and the corresponding kinetic parameters are summarized in Figure 4B and Table S13. All compounds display nanosecond‐scale excited‐state lifetimes, with average fluorescence lifetimes (*τ*_*ave*_) spanning 2.5–9.0 ns. Satisfactory fitting of the solid‐state decays required a triexponential function, pointing to additional deactivation channels that emerge in the solid phase, presumably driven by molecular aggregation, hindered rotations, and intermolecular contact. Notably, Compound **1f** exhibits the longest lifetime both in ACN solution and in the solid state, demonstrating that the thiophene moiety stabilizes the emissive state and mitigates nonradiative decay. Conversely, Compound **1d** displays the shortest average lifetime, suggesting that faster excited‐state deactivation processes remain operative even after molecular rigidification (see Equation S4).

The %*Φ*_*f*_ values summarized in Table 5 reveal a pronounced substituent‐dependent emission efficiency. Compound **1f** exhibits the highest solid‐state %*Φ*_*f*_ (10.22%), followed by **1e** (6.75%) and **1a** (4.70%), whereas Compound **1c** displays a low %Φ_f_ (2.12%), corresponding to a quenched solid‐state emission. Interestingly, despite exhibiting an average fluorescence lifetime comparable to those of Compounds **1a**, **1b**, and **1g**, Compound **1c** remains a poor emitter. This observation indicates that fluorescence efficiency in this family is not exclusively determined by excited‐state lifetime but also by the electronic characteristics of the substituents, which strongly influence the competition between radiative and nonradiative decay pathways. Overall, the %*Φ*_*f*_ values span an order of magnitude, highlighting the remarkable sensitivity of the photophysical response to subtle structural modifications within the pyrrolo[3,4‐*b*]pyridin‐5‐one core.

Compared with our previously reported pyrazole‐derived pyrrolo[3,4‐*b*]pyridin‐5‐ones, which exhibited higher solid‐state %*Φ*_*f*_ due to dual‐state emission (DSE) and ESIPT processes [14], the present compounds display a distinct solid‐state photophysical behavior. Rather than relying on ESIPT‐assisted emission, their fluorescence is mainly governed by the interplay between restricted intramolecular motions and substituent‐dependent electronic effects. These observations demonstrate that subtle structural modifications within the pyrrolo[3,4‐*b*]pyridin‐5‐one scaffold can lead to different emission mechanisms and tunable solid‐state optical properties. Using the fluorescence quantum yields %Φ_f_ and fluorescence lifetimes (*τ*_*ave*_), the radiative (*k*_*r*_), and nonradiative (*k*_*nr*_) rate constants were calculated to compare them with the photophysics observed in solution and in a polymeric matrix. Notably, in the solid state, *k*_*nr*_ increases significantly compared to that measured in polystyrene (see Tables 3 and 5). This behavior is likely driven by molecular aggregation effects (such as H‐aggregate formation or aggregation‐caused quenching), supported by the drop in %*Φ*_*f*_ and the substantial red‐shift of the emission band [60, 61, 62]. Likewise, *k*_*r*_ decreases by an order of magnitude relative to the monomeric species in solution and the polymer matrix. This suppression of the radiative pathway is a characteristic signature of intermolecular coupling in the solid state, such as H‐aggregation (characterized by symmetry‐forbidden transitions) or excimer‐like species [60, 61, 62, 63]. Because the primary objective of this study was to evaluate these molecules as sensors for liquid environments, solid‐state properties were not pursued further, and these findings represent only the preliminary steps in the solid‐state photophysical characterization of these pyrrolo[3,4‐*b*]pyridin‐5‐ones.

These solid‐state results complement the solution‐state photophysical studies and provide additional support for the proposed structure–property relationships. Although restriction of intramolecular motion is expected to suppress nonradiative decay in molecular rotors [64], the present results demonstrate that molecular rigidification alone is insufficient to ensure highly efficient solid‐state emission. Instead, both conformational restriction and electronic modulation introduced by the substituents cooperate in defining the excited‐state dynamics. The preservation of measurable fluorescence emission, together with nanosecond excited‐state lifetimes and solid‐state %*Φ*_*f*_, demonstrates that these pyrrolo[3,4‐*b*]pyridin‐5‐ones maintain their emissive behavior in the condensed phase, further supporting their potential as environmentally responsive fluorophores and promising candidates for future solid‐state luminescent applications.

## 4 Conclusions

This study investigates a series of pyrrolo[3,4‐*b*]pyridin‐5‐ones bearing systematically varied substituents to evaluate their potential as fluorescence probes for local viscosity, polarity, protic environments, and solid‐state emission. By exploiting the rotational freedom provided by the heterocyclic substituents at the R^1^, R^2^, and R^3^ positions, these pyrrolo[3,4‐*b*]pyridin‐5‐ones were identified as molecular rotors whose excited‐state dynamics are highly sensitive to their surrounding environment. Our results demonstrate that the excited‐state behavior is not governed solely by solvent polarity; a purely dielectric response would produce nearly identical fluorescence lifetimes in acetonitrile and methanol. Instead, the pronounced lifetime reduction observed in methanol confirms that these molecules function as efficient dual‐response fluorophores, where the comparative response between acetonitrile and toluene effectively probes environmental polarity, whereas the distinct sensitivity toward methanol provides a reliable indicator of solvent proticity and hydrogen‐bonding capability. In addition to their solution‐state behavior, the solid‐state photophysical characterization demonstrates that these compounds preserve their emissive properties after aggregation. All compounds exhibit nanosecond fluorescence lifetimes and measurable photoluminescence quantum yields in the condensed phase, although their emission efficiencies strongly depend on the substitution pattern. The wide variation in solution and solid‐state quantum yield highlights the critical role of electronic substituent effects in modulating the balance between radiative and nonradiative deactivation pathways, demonstrating that molecular rigidification alone is insufficient to guarantee efficient fluorescence. Overall, the combined experimental and theoretical results establish clear structure–photophysics relationships for this family of pyrrolo[3,4‐*b*]pyridin‐5‐ones. Beyond validating their molecular rotor behavior, this work demonstrates that subtle structural modifications enable fine control over both solution‐ and solid‐state photophysical properties, providing valuable design principles for the development of environmentally responsive fluorophores and next‐generation luminescent materials.

## Author Contributions

**Ivette Morales‐Salazar:** investigation, methodology, formal analysis. **Carlos E. Garduño Albino:** investigation, formal analysis. **Alejandro Valderrama‐Celestino:** investigation, formal analysis. **Federico González‐García:** formal analysis, data curation. **Alberto Rojas:** investigation, formal analysis, validation. **Annia Galano:** formal analysis, visualization, software. **Yoarhy A. Amador‐Sánchez:** investigation, writing – original draft, formal analysis. **Cesar A. Guarin:** conceptualization, writing – original draft, methodology. **Eduardo González‐Zamora:** funding acquisition, writing – review and editing, supervision. **Alejandro Islas‐Jácome:** funding acquisition, writing – review and editing, project administration, resources.

## Funding

This work was supported by SECIHTI (A1‐S‐32582, CBF‐2025‐I‐3043, and CBF‐2023‐2024‐3073).

## Conflicts of Interest

The authors declare no conflicts of interest.

## Supporting information


**Figure S1:** Steady‐state spectra for compounds **1a–g** in toluene. (A) Normalized absorption spectra at 295 nm. (B) Normalized fluorescence emission spectra. Excitation wavelength 355 nm.
**Figure S2:** Steady state spectra for compounds **1a–g** in acetonitrile. (A) Normalized absorption spectra at 250 nm. (B) Normalized fluorescence emission spectra. Excitation wavelength 355 nm.
**Figure S3:** Close‐up of the absorption zone corresponding to the S1 emissive state of pyrrolo[3,4‐*b*]pyridin‐5‐ones in (A) methanol, (B) acetonitrile, and (C) toluene. The arrows indicate the possible position of the maximum absorption of the electronic state (always to the right of the shoulder due to the shift caused by the overlap with the band of the other electronic states). For a better estimation, a spectral deconvolution can be performed. With this value, it can be verified in Table S1, that the Stokes shift is greater than 3500 cm‐1. For further discussion, see the main text.
**Table S1:** Possible absorption maximum for transitions from the excited state S1 in the solvents used in this study. The maximum of the S1 state band in nm was estimated from the shoulders of each absorption spectrum in each solvent, according to Figure S3. Meanwhile, the DFT calculation indicates a position in nm for the absorption band. Both datasets are shown in units of cm‐1. Using these data, the energy delta ($\overset{-}{v}$ **DFT –** $\overset{-}{v}$ **shoulder**) was calculated to compare theoretical and experimental data. Negative values indicate an underestimation of the absorption maximum.
**Table S2:** Estimation of the molar absorptivity coefficient (**ε**) for the molecules of this study. The spectral bands of the pyrrolo[3,4‐*b*]pyridin‐5‐ones measured in the three solvents were used (MeOH: Methanol, ACN: acetonitrile, Tol: toluene). These signals correspond to the S1 electronic state. Absorption values were taken as indicated by the arrows in Figure S3 (in the shoulders), using an optical path length of 1 cm and a concentration of 5 × 10–5 M for all pyrrolo[3,4‐*b*]pyridin‐5‐ones. All calculated **ε** values correspond to allowed transitions.
**Table S3:** Molecular orbitals, major contributions, electronic transitions, and oscillator strengths, for compound **1a** in toluene, methanol, and acetonitrile.
**Table S4:** Molecular orbitals, major contributions, electronic transitions, and oscillator strengths, for compound **1b** in toluene, methanol, and acetonitrile.
**Table S5:** Molecular orbitals, major contributions, electronic transitions, and oscillator strengths, for compound **1c** in toluene, methanol, and acetonitrile.
**Table S6:** Molecular orbitals, major contributions, electronic transitions, and oscillator strengths, for compound **1d** in toluene, methanol, and acetonitrile.
**Table S7:** Molecular orbitals, major contributions, electronic transitions, and oscillator strengths, for compound **1e** in toluene, methanol, and acetonitrile.
**Table S8:** Molecular orbitals, major contributions, electronic transitions, and oscillator strengths, for compound **1f** in toluene, methanol, and acetonitrile.
**Table S9:** Molecular orbitals, major contributions, electronic transitions, and oscillator strengths, for compound **1g** in toluene, methanol, and acetonitrile.
**Figure S4:** Normalized calculated UV–vis spectra of compounds **1a–g** in toluene.
**Figure S5:** Normalized calculated UV–vis spectra of compounds **1a–g** in acetonitrile.
**Figure S6:** Normalized calculated UV–vis spectra of compounds **1a–g** in methanol.
**Figure S7:** Decays for compounds **1a–g** measured by TCSPC. Solvents and polymeric matrices used for each measurement are specified in the respective figures. The instrument response function (IRF) is explicitly shown.
**Table S9:** Fit parameters from the TCSPC of the S1 of Pyrrolo[3,4‐*b*]pyridin‐5‐ones in MeOH.
**Table S10:** Fit parameters from the TCSPC of S1 of pyrrolo[3,4‐*b*]pyridin‐5‐ones in MeCN.
**Table S11:** Fit parameters from the TCSPC of the S1 of pyrrolo[3,4‐*b*]pyridin‐5‐ones in toluene.
**Table S12:** Fit parameters from the TCSPC of the S1 of pyrrolo[3,4‐*b*]pyridin‐5‐ones in polystyrene matrix.
**Table S13:** Fluorescence lifetime in solid‐state experiments.
**Figure S8:** Absolute quantum yield of compound **1a** in the solid state, at λex = 400 nm.
**Figure S9:** Absolute quantum yield of compound **1b** in the solid state, at λex = 400 nm.
**Figure S10:** Absolute quantum yield of compound **1c** in the solid state, at λex = 400 nm.
**Figure S11:** Absolute quantum yield of compound **1d** in the solid state, at λex = 350 nm.
**Figure S12:** Absolute quantum yield of compound **1e** in the solid state, at λex = 350 nm.
**Figure S13:** Absolute quantum yield of compound **1f** in the solid state, at λex = 350 nm.
**Figure S14:** Absolute quantum yield of compound **1g** in the solid state, at λex = 400 nm.
**Figure S15:** Steady state spectra for 1a–g compounds in polystyrene matrix. (A) Normalized absorption spectra at 300 nm. (B) Normalized fluorescence emission spectra. Excitation wavelength 355 nm.
**Table S14:** Photophysical properties of the pyrrolo[3,4‐*b*]pyridin‐5‐ones compounds in polystyrene matrix.

## Data Availability

The data that support the findings of this study are available from the corresponding author upon reasonable request.
