# Supplementary material for: Tuning the Molecular Rotor Dynamics of Pyrrolo[3,4‐b]Pyridin‐5‐Ones: Steric and Electronic Control of Environmental Sensing
Source: Luminescence. 2026 Oct 21;41(10):e70655. doi: 10.1002/bio.70655 (PMC13686252; doi:10.1002/bio.70655)
Supplement: Supplementary file 1 [file BIO-41-e70655-s001.docx]

Tuning the Molecular Rotor Dynamics of Pyrrolo[3,4-*b*]pyridin-5-ones: Steric and Electronic Control of Environmental Sensing

Ivette Morales-Salazar^a^, Carlos E. Garduño Albino^a^, Alejandro Valderrama-Celestino^b^, Federico González-García^c^, Alberto Rojas^a^, Annia Galano^a^, Yoarhy A. Amador-Sánchez^a,^*,

Cesar A. Guarin^d,^*, Eduardo González-Zamora^a,^*, Alejandro Islas-Jácome^a,^*

*^a^ Departamento de Química, Universidad Autónoma Metropolitana-Iztapalapa, Av. Ferrocarril San Rafael Atlixco 186, Col. Leyes de Reforma 1A Sección, Iztapalapa, C.P. 09310, Ciudad de México, Mexico.*

*^b^ Department of Chemistry & Biochemistry, Baylor University, Waco, Texas 76798, United States.*

*^c^ Departamento de Ingeniería de Procesos e Hidráulica. Universidad Autónoma Metropolitana-Iztapalapa, Av. Ferrocarril San Rafael Atlixco 186, Col. Leyes de Reforma 1A Sección, Iztapalapa, C.P. 09310, Ciudad de México, Mexico.*

*^d^ Departamento de Física, Universidad Autónoma Metropolitana-Iztapalapa, Av. Ferrocarril San Rafael Atlixco 186, Col. Leyes de Reforma 1A Sección, Iztapalapa, C.P. 09310, Ciudad de México, Mexico.*

| *C O N T E N T S* | *P A G E* |
| --- | --- |
| 1. Steady-state spectra for pyrrolo[3,4-*b*]pyridin-5-ones in various solvents | **S2** |
| 2. Computational results    2.1. Contour surface orbitals, orbital energies, transitions, contributions, wavelength of vertical excitation and oscillator strength for seven pyrrolo[3,4-*b*]pyridin-5-ones in toluene, methanol and acetonitrile    2.2. The UV-vis theoretical spectra for pyrrolo[3,4-*b*]pyridin-5-ones | **S6**  **S6 S20** |
| 3. Molecular coordinates for pyrrolo[3,4-*b*]pyridin-5-ones    3.1. Toluene    3.2. Acetonitrile    3.3. Methanol | **S21 S21 S30**  **S40** |
| 4. Time-resolved fluorescence section    4.1. Decay curves for pyrrolo[3,4-*b*]pyridin-5-ones in different chemical environments    4.2. Lifetimes parameters for pyrrolo[3,4-*b*]pyridin-5-ones | **S50**  **S50 S51** |
| 5. Solid-State measurements section    5.1. TRPL spectroscopy and PL in Solid-State    5.2. Quantum Yields in Solid-State    5.3. Steady- state data for pyrrolo[3,4-*b*]pyridin-5-ones in polystyrene matrix 6. Supplementary References | **S53**  **S53**  **S54**  **S56**  **S57** |

1. Steady-state spectra for pyrrolo[3,4-*b*]pyridin-5-ones in various solvents

**Figure S1**. Steady-state spectra for compounds **1a–g** in toluene. A) Normalized absorption spectra at 295 nm.

B) Normalized fluorescence emission spectra. Excitation wavelength 355 nm.

**Figure S2.** Steady state spectra for compounds **1a–g** in acetonitrile. A) Normalized absorption spectra at 250 nm. B) Normalized fluorescence emission spectra. Excitation wavelength 355 nm.

**Figure S3.** Close-up of the absorption zone corresponding to the S1 emissive state of pyrrolo[3,4-*b*]pyridin-5-ones in (A) methanol, (B) acetonitrile, and (C) toluene. The arrows indicate the possible position of the maximum absorption of the electronic state (always to the right of the shoulder due to the shift caused by the overlap with the band of the other electronic states). For a better estimation, a spectral deconvolution can be performed. With this value, it can be verified in Table S1, that the Stokes shift is greater than 3500 cm⁻¹. For further discussion, see the main text.

**Table S1.** Possible absorption maximum for transitions from the excited state S1 in the solvents used in this study. The maximum of the S1 state band in nm was estimated from the shoulders of each absorption spectrum in each solvent, according to Figure S3. Meanwhile, the DFT calculation indicates a position in nm for the absorption band. Both datasets are shown in units of cm^-1^. Using these data, the energy delta (𝝂̅**DFT –** 𝝂̅**shoulder**) was calculated to compare theoretical and experimental data. Negative values indicate an underestimation of the absorption maximum.

| **Comp.** | **Solvent** | 𝝂̅**DFT**  **(cm^-1^)** | 𝝂̅**shoulder**  **(cm^-1^)** | 𝝂̅**fluo**  **(cm^-1^)** | **Stokes shift**  𝝂̅ **shoulder -** 𝝂̅**fluo**  **(cm^-1^)** | 𝝂̅**DFT -** 𝝂̅**shoulder**  **(cm^-1^)** |
| --- | --- | --- | --- | --- | --- | --- |
| **1a** | MeOH | 29320 | 29590 | 23700 | 5890 | -270 |
|  | ACN | 28250 | 30030 | 22880 | 7150 | -1780 |
|  | Tol | 28570 | 30770 | 23260 | 7510 | -2200 |
| **1b** | MeOH | 29690 | 29330 | 22940 | 6390 | 360 |
|  | ACN | 28450 | 30120 | 24630 | 5490 | -1670 |
|  | Tol | 28840 | 30580 | 22880 | 7700 | -1740 |
| **1c** | MeOH | 29750 | 29590 | 22270 | 7320 | 160 |
|  | ACN | 28500 | 29760 | 24330 | 5430 | -1260 |
|  | Tol | 28930 | 31060 | 24940 | 6120 | -2130 |
| **1d** | MeOH | 28150 | 29500 | 22620 | 6880 | -1350 |
|  | ACN | 27280 | 29940 | 23580 | 6360 | -2660 |
|  | Tol | 27430 | 30770 | 25060 | 5710 | -3340 |
| **1e** | MeOH | 28320 | 29670 | 21690 | 7980 | -1350 |
|  | ACN | 27283 | 30300 | 22940 | 7360 | -3017 |
|  | Tol | 27510 | 31060 | 24880 | 6180 | -3550 |
| **1f** | MeOH | 27960 | 28410 | 23360 | 5050 | -450 |
|  | ACN | 27010 | 29240 | 22680 | 6560 | -2230 |
|  | Tol | 27220 | 30670 | 23870 | 6800 | -3450 |
| **1g** | MeOH | 29690 | 29940 | 23260 | 6680 | -250 |
|  | ACN | 28670 | 29590 | 22570 | 7020 | -920 |
|  | Tol | 28990 | 30120 | 23420 | 6700 | -1130 |

**Table S2.** Estimation of the molar absorptivity coefficient (**ε**) for the molecules of this study. The spectral bands of the pyrrolo[3,4-*b*]pyridin-5-ones measured in the three solvents were used (MeOH: Methanol, ACN: acetonitrile, Tol: toluene). These signals correspond to the S1 electronic state. Absorption values were taken as indicated by the arrows in Figure S3 (in the shoulders), using an optical path length of 1 cm and a concentration of 5 × 10^-5^ M for all pyrrolo[3,4-*b*]pyridin-5-ones. All calculated **ε** values correspond to allowed transitions.

| **Comp.** | **ε** **(M^-1^ cm^-1^)** | | |
| --- | --- | --- | --- |
|  | **MeOH** | **ACN** | **Tol** |
| **1a** | 1900 | 4300 | 9200 |
| **1b** | 2240 | 1560 | 5700 |
| **1c** | 2340 | 2660 | 14240 |
| **1d** | 1800 | 4880 | 12240 |
| **1e** | 2400 | 5840 | 12800 |
| **1f** | 1620 | 4420 | 12200 |
| **1g** | 1980 | 4700 | 9000 |

2. Computational Results
   2.1. Tables show the computational properties and molecular geometries of the pyrrolo[3,4-*b*]pyridin-5-ones of this study. The accompanying tables detail the contour surface orbitals, orbital energies, transitions, contributions, vertical excitation wavelengths, and oscillator strengths for the seven pyrrolo[3,4-*b*]pyridin-5-ones compounds in toluene, methanol, and acetonitrile.

**Table S3.** Molecular orbitals, major contributions, electronic transitions, and oscillator strengths, for compound **1a** in toluene, methanol, and acetonitrile.

| Toluene | Methanol | Acetonitrile |
| --- | --- | --- |
| HOMO | | |
|  |  |  |
| -0.21220 | -0.20960 | -0.20761 |
| LUMO | | |
|  |  |  |
| -0.05316 | -0.05660 | -0.05271 |
| S0→S1 f = 0.0757  λ = 341.08 nm  H→L 100% | S0→S1 f = 0.0682  λ = 354.01 nm  H→L 100% | S0→S1 f = 0.0678  λ = 350.07 nm  H→L 100% |
| H-1 | H-1 | H-1 |
|  |  |  |
| -0.22922 | -0.23152 | -0.22942 |
| H-2 | H-2 | H-2 |
|  |  |  |
| -0.23368 | -0.23765 | -0.23420 |
| S0→ S2 f = 0.0091  λ = 298.63 nm H-1 →L 82.2%  H-2→L 8.8% | S0→ S2 f = 0.0064  λ = 297.41 nm H-1 →L 93.3%  H-2→L 6.7% | S0→ S2 f = 0.0034  λ = 295.54 nm H-1 →L 84.6%  H-2→L 10.87% |

| L+1 | L+1 | L+1 |
| --- | --- | --- |
|  |  |  |
| -0.02855 | -0.02934 | -0.02795 |
| S0→ S3 f = 0.0197  λ = 289.21 nm H-2→L 85.3%  H→L+1 3.3% | S0→ S3 f = 0.0400  λ = 285.99 nm H-2→L 79.3%  H→L+1 10.0% | S0→ S3 f = 0.1737  λ = 288.13 nm  H→L+1 59.0%  H-2→L 26.8% |
| S0→ S4 f = 0.1767  λ = 283.47 nm  H→L+1 85.3%  H-2→L 6.2% | S0→ S4 f = 0.1835  λ = 288.42 nm  H→L+1 84.3%  H-2→L 13.2% | S0→ S4 f = 0.0697  λ = 286.32 nm  H-2 →L 61.5%  H→L+1 36.3% |

**Table S4.** Molecular orbitals, major contributions, electronic transitions, and oscillator strengths, for compound **1b** in toluene, methanol, and acetonitrile.

| Toluene | Methanol | Acetonitrile |
| --- | --- | --- |
| HOMO | | |
|  |  |  |
| -0.21060 | -0.20903 | -0.20702 |
| LUMO | | |
|  |  |  |
| -0.04955 | -0.05481 | -0.05063 |
| S0→S1 f = 0.0788  λ = 336.84 nm  H→L 100% | S0→S1 f = 0.0693  λ = 351.46 nm  H→L 100% | S0→S1 f = 0.0707  λ = 346.79 nm  H→L 100% |
| H-1 | H-1 | H-1 |
|  |  |  |

| -0.23130 | -0.23567 | -0.23195 |
| --- | --- | --- |
| H-2 | H-2 | H-2 |
|  |  |  |
| -0.23968 | -0.24576 | -0.24050 |
| S0→ S2 f = 0.0005  λ = 288.86 nm  H-1→L 96.9%  H→L+1 3.1% | S0→ S2 f = 0.0058  λ = 290.20 nm H-1→L 89.3%  H→L+1 10.7% | S0→ S2 f = 0.0155  λ = 289.89 nm  H-1→L 80.7%  H→L+1 19.3% |
| L+1 | L+1 | L+1 |
|  |  |  |
| -0.02611 | -0.02862 | -0.02688 |
| H-3 | H-3 | H-3 |
|  |  |  |
| -0.24255 | -0.24885 | -0.24650 |
| H-4 | H-4 | H-4 |
|  |  |  |
| -0.25836 | -0.25436 | -0.2 |
| H-5 | H-5 | H-5 |
|  |  |  |
| -0.25344 | -0.26105 | -0.25405 |
| H-6 | H-6 | H-6 |

|  |  |  |
| --- | --- | --- |
| -0.25836 | -0.26551 | -0.25840 |
| S0→ S3 f = 0.0521  λ = 284.55 nm  H-5→L 46.6%  H→L+1 26.3%  H-4→L 14.7%  H-2→L 5.8% | S0→ S3 f = 0.1973 λ = 285.82 nm H→L+1 85.2%  H-1→L 11.7%  H-2→L 3.0% | S0→ S3 f = 0.1990 λ = 286.27 nm H→L+1 78.3%  H-1→L 19.2%  H-2→L 2.5% |
| S0→ S4 f = 0.1804 λ = 280.57 nm H→L+1 68.1%  H-5→L 16.1%  H-4→L 7.5%  H-1→L 6.2%  H-3→L 2.1% | S0→ S4 f = 0.0262  λ = 274.60 nm  H-2→L 47.2%  H-6→L 43.7%  H-4→L 2.8%  H→L+1 2.4% | S0→ S4 f = 0.0214  λ = 276.74 nm  H-6→L 60.5%  H-3→L 12.3%  H-5→L 8.8%  H-2→L 6.3%  H→L+1 2.3% |

**Table S5.** Molecular orbitals, major contributions, electronic transitions, and oscillator strengths, for compound **1c** in toluene, methanol, and acetonitrile.

| Toluene | Methanol | Acetonitrile |
| --- | --- | --- |
| HOMO | | |
|  |  |  |
| -0.21080 | -0.20851 | -0.20682 |
| LUMO | | |
|  |  |  |
| -0.04936 | -0.05402 | -0.04996 |
| S0→S1 f = 0.0781  λ = 336.16 nm  H→L 100% | S0→S1 f = 0.0716  λ = 350.85 nm  H→L 100% | S0→S1 f = 0.0712  λ = 345.72 nm  H→L 100% |
| H-1 | H-1 | H-1 |
|  |  |  |

| -0.23361 | -0.23635 | -0.23368 |
| --- | --- | --- |
| H-2 | H-2 | H-2 |
|  |  |  |
| -0.23911 | -0.24552 | -0.23894 |
| S0→ S2 f = 0.0068  λ = 288.71 nm H-1→L 56.1%  H-5→L 23.1%  H-4→L 13.3% | S0→ S2 f = 0.0307  λ = 286.71 nm H-1→L 68.0%  H→L+1 32.0% | S0→ S2 f = 0.0030  λ = 286.26 nm H-1→L 84.9%  H-6→L 9.1%  H→L+1 3.8% |
| L+1 | L+1 | L+1 |
|  |  |  |
| -0.02492 | -0.01370 | -0.02519 |
| H-4 | H-4 | H-4 |
|  |  |  |
| -0.24951 | -0.25438 | -0.25297 |
| H-5 | H-5 | H-5 |
|  |  |  |
| -0.25394 | -0.26080 | -0.25402 |
| H-6 | H-6 | H-6 |
|  |  |  |
| -0.25779 | -0.26423 | -0.25763 |
| S0→ S3 f = 0.0954  λ = 280.72 nm  H→L+1 71.0%  H-1→L 16.0%  H-2→L 8.2% | S0→ S3 f = 0.1499  λ = 283.79 nm  H→L+1 62.5%  H-1→L 34.1%  H-2→L 3.4% | S0→ S3 f = 0.1611  λ = 284.71 nm  H→L+1 92.5%  H-2→L 4.6%  H-6→L 2.9% |

| S0→ S4 f = 0.0905  λ = 277.06 nm H-6→L 51.7%  H-2→L 38.8%  H→L+1 3.2% | S0→ S4 f = 0.0248  λ = 274.98 nm H-6→L 53.3%  H-2→L 40.0%  H→L+1 3.3% | S0→ S4 f = 0.0586  λ = 275.70 nm H-6→L 42.9%  H-2→L 16.4%  H-1→L 17.4% |
| --- | --- | --- |

**Table S6.** Molecular orbitals, major contributions, electronic transitions, and oscillator strengths, for compound **1d** in toluene, methanol, and acetonitrile.

| Toluene | Methanol | Acetonitrile |
| --- | --- | --- |
| HOMO | | |
|  |  |  |
| -0.20777 | -0.20524 | -0.20233 |
| LUMO | | |
|  |  |  |
| -0.05318 | -0.05653 | -0.05222 |
| S0→S1 f = 0.0531  λ = 355.27 nm  H→L 100% | S0→S1 f = 0.0491  λ = 366.52 nm  H→L 100% | S0→S1 f = 0.0475  λ = 364.53 nm  H→L 100% |
| H-1 | H-1 | H-1 |
|  |  |  |
| -0.22958 | -0.23207 | -0.22942 |
| H-2 | H-2 | H-2 |
|  |  |  |
| -0.23814 | -0.24005 | -0.23828 |
| L+1 | L+1 | L+1 |

|  |  |  |
| --- | --- | --- |
| -0.02992 | -0.02962 | -0.02792 |
| S0→ S2 f = 0.0994  λ = 294.52 nm H-1→L 81.6%  H →L+1 17.3%  H-6→L 9.3%  H-5→L 6.6% | S0→ S2 f = 0.1789  λ = 295.72 nm  H→L+1 96.2%  H-1→L 3.8% | S0→ S2 f = 0.2103  λ = 298.02 nm  H→L+1 100% |
| H-4 | H-4 | H-4 |
|  |  |  |
| -0.25024 | -0.25453 | -0.25409 |
| H-5 | H-5 | H-5 |
|  |  |  |
| -0.25353 | -0.26110 | -0.25507 |
| H-6 | H-6 | H-6 |
|  |  |  |
| -0.25891 | -0.26675 | -0.26115 |
| S0→ S3 f = 0.1295  λ = 293.55 nm  H→L+1 80.4%  H-1→L 19.6% | S0→ S3 f = 0.0599  λ = 293.97 nm H-1→L 93.5%  H-6→L 3.3%  H→L+1 3.2% | S0→ S3 f = 0.0258  λ = 290.15 nm H-1→L 90.1%  H-6→L 7.1%  H-5→L 2.7% |

| S0→ S4 f = 0.0296  λ = 282.28 nm H-2→L 43.9%  H-6→L 16.0%  H-5→L 13.5%  H-1→L 12.4%  H-4→L 5.5% | S0→ S4 f = 0.0013  λ = 281.77 nm H-2→L 100% | S0→ S4 f = 0.0055  λ = 279.14 nm H-2→L 79.5%  H-6→L 9.3%  H-5→L 5.8%  H-1→L 2.7% |
| --- | --- | --- |

**Table S7.** Molecular orbitals, major contributions, electronic transitions, and oscillator strengths, for compound **1e** in toluene, methanol, and acetonitrile.

| Toluene | Methanol | Acetonitrile |
| --- | --- | --- |
| HOMO | | |
|  |  |  |
| -0.20747 | -0.20515 | -0.20236 |
| LUMO | | |
|  |  |  |
| -0.05237 | -0.05600 | -0.05182 |
| S0→S1 f = 0.0516  λ = 353.07 nm  H→L 100% | S0→S1 f = 0.0465  λ = 366.61 nm  H→L 100% | S0→S1 f = 0.0460  λ = 363.50 nm  H→L 100% |
| H-1 | H-1 | H-1 |
|  |  |  |
| -0.22569 | -0.23140 | -0.22951 |
| H-2 | H-2 | H-2 |
|  |  |  |
| -0.24284 | -0.24594 | -0.24493 |

| S0→ S2 f = 0.0316  λ = 299.75 nm  H→L+1 100.0% | S0→ S2 f = 0.2035  λ = 295.87 nm  H→L+1 100.0% | S0→ S2 f = 0.1942  λ = 297.11 nm  H-1→L 100.0% |
| --- | --- | --- |
| L+1 | L+1 | L+1 |
|  |  |  |
| -0.02843 | -0.02916 | -0.02710 |
| S0→ S3 f = 0.1412 λ = 292.73 nm H→L+1 91.2%  H-2→L 4.6%  H-1→L 4.0 % | S0→ S3 f = 0.0153  λ = 295.72 nm H-1→L 94.9%  H→L+1 2.8%  H-5→L 2.3% | S0→ S3 f = 0.0133  λ = 291.83 nm H-1→L 83.4%  H-5→L 8.0%  H-3→L 4.9%  H-2→L 3.8% |
| S0→ S4 f= 0.0949  λ = 285.52 nm H-2→L 30.1%  H-5→L 27.2%  H-3→L 19.3%  H-1→L 14.4%  H→L+1 9.0% | S0→ S4 f = 0.0453  λ =278.63 nm  H-2→L 40.3%  H-5→L 27.4%  H-4→L 22.8%  H-1→L 4.7% | S0→ S4 f = 0.0580  λ = 279.69 nm H-2→L 31.2%  H-5→L 27.7%  H-3→L 19.6%  H-1→L 18.9%  H-4→L 2.5% |
| H-3 | H-3 | H-3 |
|  |  |  |
| -0.24810 | -0.25463 | -0.25441 |
| H-4 | H-4 | H-4 |
|  |  |  |
| -0.25195 | -0.25784 | -0.25441 |
| H-5 | H-5 | H-5 |
|  |  |  |
| -0.25683 | -0.26590 | -0.26004 |

**Table S8.** Molecular orbitals, major contributions, electronic transitions, and oscillator strengths, for compound **1f** in toluene, methanol, and acetonitrile.

| Tolueno | Metanol | Acetonitrilo |
| --- | --- | --- |
| HOMO | | |
|  |  |  |
| -0.20716 | -0.20547 | -0.20273 |
| LUMO | | |
|  |  |  |
| -0.05429 | -0.05784 | -0.05378 |
| S0→S1 f = 0.0506  λ = 357.66 nm  H→L 100% | S0→S1 f = 0.0458  λ = 370.28 nm  H→L 100% | S0→S1 f = 0.0445  λ= 367.31 nm  H→L 100% |
| H-1 | H-1 | H-1 |
|  |  |  |
| -0.23395 | -0.23627 | -0.23520 |
| H-2 | H-2 | H-2 |
|  |  |  |
| -0.24133 | -0.24419 | -0.24292 |
| L+1 | L+1 | L+1 |
|  |  |  |

| -0.03284 | -0.03414 | -0.03303 |
| --- | --- | --- |
| L+2 | L+2 | L+2 |
|  |  |  |
| -0.02645 | -0.02878 | -0.02723 |
| S0→ S2 f = 0.2030 λ = 298.21 nm H→L+1 91.4%  H→L+2 8.6% | S0→ S2 f = 0.1883 λ = 301.63 nm H→L+1 90.3%  H→L+2 9.7% | S0→ S2 f = 0.1896 λ = 304.13 nm H→L+1 88.4%  H→L+2 8.8% |
| H-3 | H-3 | H-3 |
|  |  |  |
| -0.24376 | -0.24651 | -0.24641 |
| H-4 | H-4 | H-4 |
|  |  |  |
| -0.25034 | -0.25470 | -0.5416 |
| H-5 | H-5 | H-5 |
|  |  |  |
| -0.25502 | -0.25826 | -0.25697 |
| H-6 | H-6 | H-6 |
|  |  |  |
| -0.25688 | -0.25931 | -0.25795 |
| H-7 | H-7 | H-7 |

| -0.25828 | -0.26531 | -0.26067 |
| --- | --- | --- |
| S0→ S3 f = 0.0238  λ = 291.29 nm H-1→L 67.1%  H-6→L 12.6%  H-7→L 5.9%  H-4→L 3.8%  H-3→L 3.7% | S0→ S3 f = 0.0406  λ = 291.39 nm H-1→L 94.8%  H-7→L 2.3% | S0→ S3 f = 0.0319 λ = 291.30 nm H→L+2 89.0%  H→L+1 11.0% |
| S0→ S4 f = 0.0119  λ = 283.25 nm  H-2→L 27.7%  H-1→L 19.0%  H→L+2 16.8%  H-6→L 9.4%  H-3→L 7.9%  H→L+2 6.1%  H-7→L 5.1%  H-4→L 2.8% | S0→ S4 f = 0.0244 λ = 288.72 nm H→L+2 89.5%  H→L+1 10.5% | S0→ S4 f = 0.0314  λ = 286.49 nm H-1→L 81.9%  H-7→L 7.7%  H-5→L 2.8%  H-3→L 2.3% |

**Table S9.** Molecular orbitals, major contributions, electronic transitions, and oscillator strengths, for compound **1g** in toluene, methanol, and acetonitrile.

| Toluene | Methanol | Acetonitrile |
| --- | --- | --- |
| HOMO | | |
|  |  |  |
| -0.21854 | -0.21484 | -0.21362 |
| LUMO | | |
|  |  |  |
| -0.05553 | -0.05796 | -0.05479 |
| S0→S1 f = 0.0594  λ = 336.82 nm  H→L 100% | S0→S1 f = 0.0578  λ = 348.74 nm  H→L 100% | S0→S1 f = 0.0582  λ = 344.96 nm  H→L 100% |
| H-1 | H-1 | H-1 |

| -0.2427 | -0.24585 | -0.24527 |
| --- | --- | --- |
| H-2 | H-2 | H-2 |
|  |  |  |
| -0.24746 | -0.25443 | -0.24990 |
| H-3 | H-3 | H-3 |
|  |  |  |
| -0.25039 | -0.25509 | -0.25412 |
| H-4 | H-4 | H-4 |
|  |  |  |
| -0.25523 | -0.25932 | -0.25756 |
| S0→ S2 f = 0.0152  λ = 289.75 nm H-2→L 29.5%  H-1→L 22.1%  H-5→L 16.2%  H-4→L 10.1%  H→L+3 9.4%  H→L+2 7.0% | S0→ S2 f = 0.1451  λ = 293.11 nm  H→L+1 47.7%  H→L+2 39.6%  H→L+3 12.7% | S0→ S2 f = 0.1391  λ = 293.14 nm  H→L+1 84.8%  H→L+3 8.5%  H→L+4 6.6% |
| L+1 | L+1 | L+1 |
|  |  |  |

| -0.04225 | -0.04017 | -0.03893 |
| --- | --- | --- |
| L+2 | L+2 | L+2 |
|  |  |  |
| -0.03436 | -0.03974 | -0.03723 |
| L+3 | L+3 | L+3 |
|  |  |  |
| -0.03216 | -0.02973 | -0.02934 |
| S0→ S3 f=0.0435  λ = 286.37 nm  H→L+1 97.3%  H→L+2 2.7 % | S0→ S3 f=0.0020  λ = 284.46 nm  H→L+2 58.9%  H→L+1 37.4%  H→L+4 3.7% | S0→ S3 f=0.0018  λ = 283.82 nm  H-1→L 23.4%  H-2→L 20.0%  H-4→L 15.5%  H→L+1 9.9%  H→L+4 9.8%  H→L+3 5.0% |
| S0→ S4 f = 0.1319 | S0→ S4 f = 0.0029 | S0→ S4 f = 0.0140 |
| λ = 283.55 nm | λ = 284.12 nm | λ = 281.42 nm |
| H→L+3 42.4% | H→L+3 30.8% | H→L+2 78.6% |
| H→L+2 31.4% | H-1→L 30.0% | H→L+4 12.1% |
| H-2→L 6.6% | H→L+2 12.0% | H-2→L 3.6% |
| H→L+4 6.1% | H-4→L 11.1% | H-4→L 3.4% |
| H→L+1 5.1% | H-3→L 7.6% | H→L+1 2.3% |
| L+4 | L+4 | L+4 |
|  |  |  |
| -0.02876 | -0.02772 | -0.02827 |

2.2. The UV-vis theoretical spectra for pyrrolo[3,4-*b*]pyridin-5-ones

**Figure S4.** Normalized calculated UV-vis spectra of compounds **1a–g** in toluene.

**Figure S5.** Normalized calculated UV-vis spectra of compounds **1a–g** in acetonitrile.

**Figure S6.** Normalized calculated UV-vis spectra of compounds **1a–g** in methanol.

3. Molecular coordinates for pyrrolo[3,4-*b*]pyridin-5-ones
   3.1. Toluene

# 1a

|  | X | Y | Z |
| --- | --- | --- | --- |
| C | -1.27941700 | -1.94739500 | -0.18015300 |
| H | -1.55658400 | -2.97748800 | -0.37543500 |
| C | -2.24047100 | -0.92435000 | -0.08419400 |
| N | -0.48042600 | 0.71238500 | 0.31403100 |
| C | 0.05432600 | -1.60427700 | 0.00312800 |
| C | 1.29159600 | -2.42568300 | -0.01809600 |
| C | 0.39172400 | -0.28725900 | 0.27293600 |
| C | 1.89597800 | -0.16085700 | 0.46562200 |
| H | 2.32240500 | 0.48734200 | -0.31192600 |
| N | 2.32530300 | -1.55542500 | 0.26565300 |
| O | 1.40090000 | -3.63064400 | -0.23634500 |
| C | 3.72625500 | -1.96005900 | 0.25720600 |
| H | 4.19260400 | -1.69588700 | 1.21309200 |
| H | 3.72450300 | -3.05115800 | 0.16955300 |
| C | 4.52975900 | -1.33791900 | -0.84020000 |
| C | 5.58110600 | -0.46575000 | -0.84684700 |
| H | 6.04559200 | -0.02354400 | 0.02501700 |
| C | 5.93439600 | -0.25594600 | -2.22310200 |
| H | 6.72273900 | 0.37534900 | -2.61117700 |
| C | 5.06777200 | -1.01225200 | -2.95253200 |
| H | 4.93175600 | -1.18415100 | -4.01051000 |
| O | 4.20815800 | -1.67879200 | -2.12811800 |
| C | 2.31001800 | 0.38756800 | 1.80221600 |
| C | 2.26892500 | -0.09550600 | 3.07963800 |
| H | 1.90438700 | -1.06850200 | 3.38241400 |
| C | 2.79988100 | 0.93732200 | 3.92269400 |
| H | 2.92224300 | 0.90873200 | 4.99740500 |
| C | 3.12383700 | 1.97179800 | 3.09676600 |
| H | 3.54677900 | 2.95328500 | 3.25439300 |
| O | 2.83641100 | 1.65024900 | 1.80237500 |
| C | -1.77300300 | 0.41883300 | 0.10770000 |
| C | -2.73406800 | 1.59838800 | 0.02645100 |
| H | -3.57363800 | 1.30184100 | -0.61145800 |
| H | -3.15946100 | 1.79722200 | 1.01949100 |
| C | -2.11929100 | 2.87540100 | -0.51281900 |
| C | -1.98183400 | 3.06557200 | -1.89599300 |
| C | -1.69356500 | 3.89607900 | 0.34714500 |
| C | -1.42915100 | 4.24241400 | -2.40731100 |
| H | -2.31190100 | 2.28492600 | -2.57905800 |
| C | -1.14155100 | 5.07662800 | -0.15882200 |
| H | -1.78831300 | 3.76338600 | 1.42277300 |

| C | -1.00629200 | 5.25358200 | -1.53872000 |
| --- | --- | --- | --- |
| H | -1.33291600 | 4.37114000 | -3.48298400 |
| H | -0.81583100 | 5.85657500 | 0.52551700 |
| H | -0.57829800 | 6.17147700 | -1.93443600 |
| N | -3.61376700 | -1.18396400 | -0.23220300 |
| C | -4.01627800 | -2.43174600 | -0.89095600 |
| H | -3.69583500 | -3.33024300 | -0.33773600 |
| H | -3.55637000 | -2.47595200 | -1.88506200 |
| C | -5.53192200 | -2.46050800 | -1.03732700 |
| H | -5.86872600 | -1.59545900 | -1.62726900 |
| H | -5.83938800 | -3.37213300 | -1.56001300 |
| C | -4.54801500 | -0.74730200 | 0.83357800 |
| H | -3.98331500 | -0.37621300 | 1.69482000 |
| H | -5.19067400 | 0.06920100 | 0.47840900 |
| C | -5.42573700 | -1.90705200 | 1.29669100 |
| H | -4.80846100 | -2.69219100 | 1.75882100 |
| H | -6.13830900 | -1.55377400 | 2.04911400 |
| O | -6.20329700 | -2.45995000 | 0.23154700 |
| **1b** | X | Y | Z |
| C | 1.12270000 | -1.96245300 | 0.02603400 |
| C | 2.22468800 | -1.08809600 | 0.00950800 |
| H | 1.24331200 | -3.03663900 | -0.05961800 |
| N | 0.73123700 | 0.82066300 | 0.24768700 |
| C | -0.14292800 | -1.41114200 | 0.17838100 |
| C | -1.48648300 | -2.04134300 | 0.25453700 |
| C | -0.27862800 | -0.03866100 | 0.31070100 |
| C | -1.74416800 | 0.32537300 | 0.48803700 |
| N | -2.37890200 | -1.00740000 | 0.43666400 |
| O | -1.76254400 | -3.23868400 | 0.18686800 |
| C | -3.81541000 | -1.20568800 | 0.51126100 |
| H | -3.97690600 | -2.28623100 | 0.55659600 |
| H | -4.19812200 | -0.76288900 | 1.44211500 |
| C | -4.59315500 | -0.61210400 | -0.67083400 |
| H | -4.40263200 | 0.46776100 | -0.72983000 |
| C | -6.11024500 | -0.89164200 | -0.57539300 |
| H | -6.29909100 | -1.75114900 | 0.08147100 |
| H | -6.66152600 | -0.03672100 | -0.17152800 |
| C | -6.49662300 | -1.23363300 | -2.02428500 |
| H | -7.37763500 | -1.88066200 | -2.09235000 |
| H | -6.69413000 | -0.32045200 | -2.59903100 |
| C | -5.22181100 | -1.90826700 | -2.52892400 |
| H | -5.08286700 | -1.85442100 | -3.61330700 |
| H | -5.18051900 | -2.96597000 | -2.22459000 |
| O | -4.14711300 | -1.17790600 | -1.91859500 |
| C | -2.05547800 | 1.08551400 | 1.74576100 |

| C | -1.83263300 | 0.86207300 | 3.07510200 |
| --- | --- | --- | --- |
| H | -1.33301100 | 0.00268100 | 3.50287400 |
| C | -2.38613600 | 1.98341200 | 3.77751000 |
| H | -2.39119500 | 2.15075100 | 4.84654200 |
| C | -2.90437100 | 2.80955200 | 2.82524100 |
| H | -3.41179000 | 3.76277400 | 2.85594100 |
| O | -2.71539600 | 2.27616600 | 1.58449900 |
| C | 1.96330400 | 0.32108800 | 0.06844500 |
| C | 3.08509600 | 1.33492900 | -0.12289700 |
| H | 3.86960500 | 0.85985100 | -0.72119900 |
| C | 4.54545500 | -1.16154800 | 0.90201400 |
| C | 3.73923200 | -2.92470800 | -0.62571800 |
| C | 5.27484700 | -2.38033600 | 1.46040400 |
| H | 4.04995500 | -0.64443300 | 1.73035500 |
| H | 5.28190300 | -0.47182000 | 0.46920000 |
| C | 5.22871200 | -3.19169100 | -0.79913400 |
| H | 3.31199000 | -3.69631400 | 0.03665800 |
| H | 3.24334000 | -3.01706800 | -1.59887200 |
| H | 4.57363500 | -3.03315400 | 2.00206200 |
| H | 6.04505700 | -2.05666100 | 2.16786100 |
| H | 5.38157600 | -4.19850200 | -1.20237300 |
| H | 5.65906300 | -2.46840600 | -1.50702100 |
| N | 3.54213300 | -1.56400700 | -0.11429500 |
| O | 5.94924600 | -3.12124900 | 0.44148100 |
| H | 3.53931900 | 1.56823000 | 0.84991900 |
| C | 2.65630800 | 2.62610900 | -0.79284000 |
| C | 2.58362900 | 2.70705700 | -2.19131300 |
| C | 2.33706500 | 3.76423200 | -0.04045100 |
| C | 2.19926200 | 3.89283400 | -2.82280800 |
| H | 2.83126300 | 1.83353800 | -2.79187300 |
| C | 1.95395200 | 4.95372000 | -0.66673200 |
| H | 2.38250000 | 3.71756800 | 1.04551900 |
| C | 1.88272700 | 5.02206400 | -2.06116400 |
| H | 2.15091700 | 3.93542400 | -3.90852700 |
| H | 1.71019400 | 5.82613100 | -0.06478700 |
| H | 1.58673200 | 5.94706900 | -2.55015200 |
| H | -2.07922100 | 0.93844300 | -0.35912000 |
| **1c** | X | Y | Z |
| C | 1.04778900 | -1.97766700 | 0.08098000 |
| C | 2.15868700 | -1.11747100 | 0.01648600 |
| H | 1.15649600 | -3.05543000 | 0.03009500 |
| N | 0.69136700 | 0.81581900 | 0.21021100 |
| C | -0.20956500 | -1.40665900 | 0.23235200 |
| C | -1.56011600 | -2.01410500 | 0.34190900 |
| C | -0.32775400 | -0.02855200 | 0.31606300 |

| C | -1.78604800 | 0.35873700 | 0.50420400 |
| --- | --- | --- | --- |
| N | -2.43654600 | -0.96203500 | 0.51816300 |
| O | -1.85725600 | -3.20745000 | 0.29911600 |
| C | -3.87902200 | -1.14222900 | 0.50654600 |
| H | -4.05360800 | -2.22084500 | 0.54757700 |
| H | -4.31745000 | -0.68866600 | 1.40589800 |
| C | -4.57580000 | -0.54789400 | -0.72493200 |
| H | -4.39169400 | 0.53394300 | -0.76459200 |
| C | -6.09351300 | -0.84026300 | -0.73752000 |
| H | -6.32177200 | -1.69918900 | -0.09279500 |
| H | -6.67986400 | 0.01084000 | -0.37757700 |
| C | -6.37266300 | -1.19026600 | -2.20916900 |
| H | -7.23876900 | -1.84841200 | -2.33635200 |
| H | -6.54116100 | -0.28109800 | -2.79936200 |
| C | -5.05728800 | -1.84959500 | -2.62176800 |
| H | -4.84387500 | -1.79658400 | -3.69395600 |
| H | -5.02281600 | -2.90567400 | -2.31133700 |
| O | -4.03762300 | -1.10235900 | -1.94075500 |
| C | -2.04495100 | 1.17596500 | 1.74724200 |
| C | -2.39655700 | 0.72915700 | 2.98828700 |
| H | -2.59665700 | -0.25006400 | 3.39743900 |
| C | -2.21273200 | 2.90935800 | 3.16011100 |
| H | -2.25382500 | 3.83233700 | 3.72012800 |
| C | 1.91522800 | 0.29540600 | 0.03400000 |
| C | 3.04947800 | 1.28650900 | -0.19836500 |
| H | 3.80586400 | 0.79077900 | -0.81663800 |
| C | 4.48863700 | -1.20233400 | 0.88165800 |
| C | 3.64321100 | -2.98435800 | -0.60316100 |
| C | 5.22212800 | -2.41757000 | 1.44258500 |
| H | 4.00678400 | -0.67530800 | 1.71210700 |
| H | 5.22089600 | -0.51952000 | 0.43097300 |
| C | 5.12664300 | -3.26744500 | -0.80172700 |
| H | 3.22276800 | -3.73956700 | 0.08208700 |
| H | 3.12661400 | -3.09097300 | -1.56400100 |
| H | 4.52820800 | -3.05910800 | 2.00662800 |
| H | 6.00719400 | -2.08855500 | 2.13093600 |
| H | 5.26359500 | -4.28369500 | -1.18688300 |
| H | 5.54743300 | -2.56222200 | -1.53318700 |
| N | 3.46931500 | -1.61196500 | -0.11572000 |
| O | 5.87377600 | -3.17603700 | 0.42183500 |
| H | 3.53858400 | 1.51490100 | 0.75852700 |
| C | 2.62814700 | 2.58370200 | -0.86079400 |
| C | 2.51710600 | 2.66283100 | -2.25687400 |
| C | 2.35434700 | 3.73058100 | -0.10344100 |
| C | 2.13933000 | 3.85470700 | -2.88094800 |
| H | 2.72966000 | 1.78295600 | -2.86141600 |
| C | 1.97813800 | 4.92631800 | -0.72222800 |

| H | 2.43182300 | 3.68593400 | 0.98082400 |
| --- | --- | --- | --- |
| C | 1.86779200 | 4.99232300 | -2.11441300 |
| H | 2.06168100 | 3.89562700 | -3.96500900 |
| H | 1.77238800 | 5.80615100 | -0.11670600 |
| H | 1.57853300 | 5.92236900 | -2.59788300 |
| C | -1.92570800 | 2.60666200 | 1.86467400 |
| H | -1.64692000 | 3.30071600 | 1.08277400 |
| O | -2.50474400 | 1.77430200 | 3.86020400 |
| H | -2.12194200 | 0.93289500 | -0.37062900 |
| **1d** | X | Y | Z |
| N | 2.31399400 | -1.31779500 | 0.30294100 |
| C | 1.81369600 | 0.05666700 | 0.52164000 |
| C | 0.32853700 | -0.13510500 | 0.26476300 |
| C | 0.06514700 | -1.45453800 | -0.07400500 |
| C | 1.33964200 | -2.21705400 | -0.06988300 |
| N | -0.59410800 | 0.81771400 | 0.31135500 |
| C | -1.86140500 | 0.47601900 | 0.03113600 |
| C | -2.25538100 | -0.87559600 | -0.24794400 |
| C | -1.24198900 | -1.84825900 | -0.32908300 |
| O | 1.51714400 | -3.40597600 | -0.33610800 |
| C | 3.73104700 | -1.66833000 | 0.34304100 |
| C | 4.48123600 | -1.34367900 | -0.91049100 |
| C | 2.15383200 | 0.59383400 | 1.88554900 |
| N | -3.60852800 | -1.17546000 | -0.50133200 |
| C | -2.86824100 | 1.61841700 | -0.01352100 |
| C | -2.31289100 | 2.91676300 | -0.56833100 |
| C | -1.91139300 | 3.95592300 | 0.28132600 |
| C | -1.40846300 | 5.15295600 | -0.23696300 |
| C | -1.29898900 | 5.32814400 | -1.61941500 |
| C | -1.69910200 | 4.29902800 | -2.47780700 |
| C | -2.20310700 | 3.10563900 | -1.95427500 |
| C | -3.96859000 | -2.32608600 | -1.33796400 |
| C | -4.31815600 | -3.57428500 | -0.52164900 |
| O | -5.33301000 | -3.28203200 | 0.44140100 |
| C | -4.92247200 | -2.23543400 | 1.32093700 |
| C | -4.61494700 | -0.94939900 | 0.55038800 |
| C | 4.91687900 | -2.09413500 | -1.96471200 |
| C | 5.57038500 | -1.19308700 | -2.87182700 |
| C | 5.48391500 | 0.04250600 | -2.30561000 |
| O | 4.82680200 | -0.02872000 | -1.11003900 |
| C | 2.83456600 | 1.74494400 | 2.15235000 |
| O | 2.95523500 | 1.93191800 | 3.49738700 |
| C | 2.34190900 | 0.87083700 | 4.10207800 |
| C | 1.83429400 | 0.02060800 | 3.16982600 |
| H | 2.23566900 | 0.72958500 | -0.23667900 |

| H | -1.46345100 | -2.88325200 | -0.56633800 |
| --- | --- | --- | --- |
| H | 4.18563100 | -1.15951500 | 1.20054700 |
| H | 3.78999600 | -2.74575900 | 0.51467500 |
| H | -3.72303700 | 1.29700300 | -0.61760900 |
| H | -3.25446600 | 1.80263600 | 0.99865500 |
| H | -1.98532100 | 3.82400000 | 1.35860800 |
| H | -1.10115700 | 5.94687200 | 0.43982400 |
| H | -0.90935200 | 6.25885600 | -2.02472600 |
| H | -1.62359800 | 4.42682000 | -3.55523300 |
| H | -2.51595000 | 2.31111100 | -2.62935000 |
| H | -3.16172000 | -2.53606400 | -2.04597300 |
| H | -4.85474400 | -2.04053000 | -1.92340400 |
| H | -3.43197400 | -3.97730700 | -0.00627600 |
| H | -4.72614700 | -4.35567300 | -1.17067700 |
| H | -5.75269800 | -2.07428600 | 2.01642000 |
| H | -4.04016000 | -2.55898600 | 1.89819100 |
| H | -5.53815100 | -0.58395900 | 0.07902300 |
| H | -4.26539300 | -0.18685000 | 1.24900500 |
| H | 4.77995400 | -3.16112800 | -2.08161800 |
| H | 6.04048600 | -1.43648400 | -3.81553100 |
| H | 5.82207700 | 1.02581400 | -2.59917500 |
| H | 3.27090200 | 2.50821800 | 1.52399600 |
| H | 2.35496800 | 0.87249300 | 5.18237000 |
| H | 1.29902200 | -0.90014400 | 3.36207800 |
| **1e** | X | Y | Z |
| N | 2.21175000 | -1.45114400 | 0.47618100 |
| C | 1.76242400 | -0.05643300 | 0.65916700 |
| C | 0.27701200 | -0.18755700 | 0.37006000 |
| C | -0.04497200 | -1.51426900 | 0.11825000 |
| C | 1.19690300 | -2.32462200 | 0.15142100 |
| N | -0.60915500 | 0.80329100 | 0.33263300 |
| C | -1.88544500 | 0.49526000 | 0.05131000 |
| C | -2.33591900 | -0.85441100 | -0.13055800 |
| C | -1.36393600 | -1.87085000 | -0.12688800 |
| O | 1.32318900 | -3.52816100 | -0.07755000 |
| C | 3.61764300 | -1.85600600 | 0.36442200 |
| C | 4.20010600 | -1.66187300 | -1.00096500 |
| C | 2.03985200 | 0.48756800 | 2.07201800 |
| N | -3.69924400 | -1.11287700 | -0.37728400 |
| C | -2.83526400 | 1.67359500 | -0.11655000 |
| C | -2.20669300 | 2.88802600 | -0.77455800 |
| C | -1.95043200 | 4.05561900 | -0.04485700 |
| C | -1.38376600 | 5.17756900 | -0.65867800 |
| C | -1.06262200 | 5.14549100 | -2.01798400 |
| C | -1.31639200 | 3.98551300 | -2.75811400 |

| C | -1.88479800 | 2.86939700 | -2.14063700 |
| --- | --- | --- | --- |
| C | -4.10258300 | -2.31565000 | -1.11659600 |
| C | -4.50172700 | -3.47862000 | -0.20277200 |
| O | -5.50430700 | -3.06940300 | 0.73029700 |
| C | -5.05099100 | -1.97184200 | 1.52200100 |
| C | -4.69864900 | -0.76567800 | 0.64886000 |
| C | 4.46923100 | -2.51078800 | -2.03681500 |
| C | 5.03120100 | -1.71403800 | -3.09077600 |
| C | 5.06174900 | -0.43544100 | -2.62217600 |
| O | 4.56222100 | -0.38364600 | -1.35245900 |
| O | 3.45430000 | 0.49304700 | 2.30622700 |
| C | 3.67160800 | 1.36079600 | 3.42427600 |
| C | 2.70384400 | 2.53889700 | 3.22126300 |
| C | 1.59123500 | 1.94837400 | 2.31322800 |
| H | 2.25810000 | 0.59771300 | -0.07033100 |
| H | -1.62396700 | -2.90987500 | -0.29795300 |
| H | 4.18922300 | -1.30037000 | 1.10924400 |
| H | 3.66177800 | -2.91959300 | 0.60992800 |
| H | 1.56517900 | -0.18761900 | 2.80357700 |
| H | -3.69286800 | 1.33702700 | -0.70931100 |
| H | -3.23394700 | 1.96889200 | 0.86404700 |
| H | -2.19221800 | 4.08700800 | 1.01569600 |
| H | -1.19259000 | 6.07429500 | -0.07363600 |
| H | -0.62159000 | 6.01576500 | -2.49798300 |
| H | -1.07331500 | 3.95101100 | -3.81765800 |
| H | -2.07900100 | 1.97184800 | -2.72499300 |
| H | -3.30373600 | -2.61547500 | -1.80096100 |
| H | -4.97597600 | -2.04498300 | -1.72729600 |
| H | -3.63303800 | -3.87433200 | 0.34673800 |
| H | -4.94037200 | -4.29241900 | -0.78870900 |
| H | -5.87160400 | -1.72309700 | 2.20300000 |
| H | -4.17911100 | -2.28054800 | 2.12266100 |
| H | -5.60813900 | -0.41027900 | 0.14448500 |
| H | -4.32490000 | 0.04141300 | 1.28193600 |
| H | 4.28065900 | -3.57620300 | -2.04744100 |
| H | 5.36642700 | -2.05120300 | -4.06283500 |
| H | 5.39279800 | 0.50600000 | -3.03636800 |
| H | 4.72900200 | 1.63916000 | 3.42051700 |
| H | 3.45450300 | 0.81780700 | 4.35818400 |
| H | 2.31432200 | 2.91008400 | 4.17499400 |
| H | 3.20892400 | 3.37158800 | 2.72129000 |
| H | 0.60170600 | 1.98646800 | 2.77580600 |
| H | 1.52536500 | 2.49216000 | 1.36642800 |
| **1f** | X | Y | Z |
| N | 2.06366100 | -1.33196400 | 0.29641800 |

| C | 1.57042200 | 0.04652000 | 0.48471100 |
| --- | --- | --- | --- |
| C | 0.08519300 | -0.13629000 | 0.21215500 |
| C | -0.18208100 | -1.45667500 | -0.11739700 |
| C | 1.08763500 | -2.22612800 | -0.09198900 |
| N | -0.82844800 | 0.82513700 | 0.23375000 |
| C | -2.09428300 | 0.48985000 | -0.05958600 |
| C | -2.49551900 | -0.86279000 | -0.32493900 |
| C | -1.48855800 | -1.84372500 | -0.38509000 |
| O | 1.26316900 | -3.41478800 | -0.35649300 |
| C | 3.47832900 | -1.69196700 | 0.34854800 |
| C | 4.25330300 | -1.36008700 | -0.90429100 |
| C | 1.91792900 | 0.63931900 | 1.82549300 |
| N | -3.84791800 | -1.15518600 | -0.58723000 |
| C | -3.09008000 | 1.63997600 | -0.13921700 |
| C | -2.51525800 | 2.92161300 | -0.71296000 |
| C | -2.10586800 | 3.97050600 | 0.12088300 |
| C | -1.58519000 | 5.15188100 | -0.41553300 |
| C | -1.46506100 | 5.30123300 | -1.80018900 |
| C | -1.87242400 | 4.26199300 | -2.64279100 |
| C | -2.39473300 | 3.08457700 | -2.10129700 |
| C | -4.20992300 | -2.31748200 | -1.40676500 |
| C | -4.57627000 | -3.54833300 | -0.57171700 |
| O | -5.59741500 | -3.23110100 | 0.37667200 |
| C | -5.18565800 | -2.17287600 | 1.24159500 |
| C | -4.86177900 | -0.90277300 | 0.45167500 |
| C | 4.40579100 | -2.13470800 | -2.02825900 |
| C | 5.19650300 | -1.51565500 | -3.04498200 |
| C | 5.64309900 | -0.27173600 | -2.68507800 |
| S | 5.10362800 | 0.15925200 | -1.09319800 |
| S | 1.54111600 | -0.16860700 | 3.33322600 |
| C | 2.19465100 | 1.14890700 | 4.25699900 |
| C | 2.67090700 | 2.14753400 | 3.45150100 |
| C | 2.51331800 | 1.85267600 | 2.06119900 |
| H | 2.00199300 | 0.70808900 | -0.27969400 |
| H | -1.71476400 | -2.87946500 | -0.61412000 |
| H | 3.92360400 | -1.20721200 | 1.22354200 |
| H | 3.51666400 | -2.77240300 | 0.51055500 |
| H | -3.93984900 | 1.31427900 | -0.74801300 |
| H | -3.48738400 | 1.84804900 | 0.86394400 |
| H | -2.18760200 | 3.85852800 | 1.19985100 |
| H | -1.27245900 | 5.95401100 | 0.24902100 |
| H | -1.06121300 | 6.21961900 | -2.21948900 |
| H | -1.78833300 | 4.36935800 | -3.72180900 |
| H | -2.71311700 | 2.28191700 | -2.76410200 |
| H | -3.39872700 | -2.54612700 | -2.10398500 |
| H | -5.08867700 | -2.03505200 | -2.00459700 |
| H | -3.69783900 | -3.94866300 | -0.04121700 |

| H | -4.98442900 | -4.33794700 | -1.21058300 |
| --- | --- | --- | --- |
| H | -6.02025700 | -1.99285300 | 1.92708300 |
| H | -4.31062100 | -2.49286500 | 1.83156500 |
| H | -5.77779700 | -0.53832600 | -0.03417600 |
| H | -4.51192500 | -0.13123900 | 1.14012200 |
| H | 3.96223900 | -3.12120600 | -2.12117700 |
| H | 5.42667800 | -1.97992700 | -3.99870200 |
| H | 6.26125300 | 0.41254800 | -3.25324700 |
| H | 2.19610700 | 1.09953100 | 5.33912500 |
| H | 3.12333300 | 3.05823600 | 3.83047700 |
| H | 2.82375500 | 2.51784200 | 1.26111100 |
| **1g** | X | Y | Z |
| C | -0.08585500 | 0.19391000 | -0.37877400 |
| C | -0.18071100 | -1.17435900 | -0.58778500 |
| C | 0.93605800 | -1.97722900 | -0.38478000 |
| C | 2.12655700 | -1.35592700 | 0.02591300 |
| C | 2.12882200 | 0.06925600 | 0.17036000 |
| C | -1.41317200 | 0.86233600 | -0.67240100 |
| C | -1.56079100 | -1.49754600 | -1.02873700 |
| H | 0.87622900 | -3.05049500 | -0.53216500 |
| N | 1.02915300 | 0.81737500 | -0.01126800 |
| N | -2.26733500 | -0.31190000 | -0.97405400 |
| O | -2.01069700 | -2.58029400 | -1.39763900 |
| C | -1.98127300 | 1.74763000 | 0.42967900 |
| C | -1.79824800 | 1.46513400 | 1.78944300 |
| C | -2.38010600 | 2.30737600 | 2.73578900 |
| H | -1.20580000 | 0.60979600 | 2.09959500 |
| C | -3.25679900 | 3.60231200 | 0.92207700 |
| C | -3.12844000 | 3.40121700 | 2.29693400 |
| H | -2.24863900 | 2.11425000 | 3.79748200 |
| H | -3.82859700 | 4.44351500 | 0.53516300 |
| H | -3.59769800 | 4.08486600 | 2.99852800 |
| C | -3.58614100 | -0.17953700 | -1.58674000 |
| H | -3.78534300 | 0.89351700 | -1.67751000 |
| H | -3.55313500 | -0.59309900 | -2.60259700 |
| C | -4.71915800 | -0.84022000 | -0.82001700 |
| C | -4.80442300 | -0.83068400 | 0.57524200 |
| C | -5.76629200 | -1.45273600 | -1.52089700 |
| C | -5.91010800 | -1.41301700 | 1.19474200 |
| H | -4.01351000 | -0.38118300 | 1.16929000 |
| H | -5.73459700 | -1.50128000 | -2.60887600 |
| C | -6.90234300 | -1.98530900 | 0.39633700 |
| H | -6.00329300 | -1.42486400 | 2.27737400 |
| H | -7.78007500 | -2.44648600 | 0.84521300 |
| C | 3.40452100 | 0.82133200 | 0.52096400 |

| H | 3.41759500 | 1.01233400 | 1.60311700 |
| --- | --- | --- | --- |
| H | 4.25400400 | 0.16488600 | 0.30908800 |
| C | 3.58012700 | 2.13739400 | -0.21428800 |
| C | 3.29370100 | 3.36031500 | 0.40653500 |
| C | 4.05227500 | 2.15287500 | -1.53557900 |
| C | 3.47183800 | 4.56935800 | -0.27270000 |
| H | 2.92443800 | 3.36737100 | 1.42984400 |
| C | 4.22954300 | 3.35801900 | -2.21996500 |
| H | 4.28547400 | 1.21299800 | -2.03287400 |
| C | 3.93959200 | 4.57236600 | -1.58954700 |
| H | 3.24374800 | 5.50757200 | 0.22783600 |
| H | 4.59715000 | 3.34871300 | -3.24348800 |
| H | 4.07968800 | 5.51136600 | -2.11961600 |
| C | 3.72059000 | -2.19978800 | 1.67635200 |
| C | 3.44874300 | -3.37525300 | -0.44220500 |
| C | 5.17500200 | -2.66104300 | 1.80143700 |
| H | 3.05561900 | -2.91693500 | 2.19523400 |
| H | 3.58619500 | -1.23131500 | 2.16390700 |
| C | 4.90195100 | -3.86131400 | -0.39757700 |
| H | 2.79649200 | -4.14776800 | 0.00857200 |
| H | 3.12773700 | -3.23692400 | -1.47961500 |
| C | 5.40450700 | -3.97726200 | 1.04745800 |
| H | 5.42237300 | -2.77437700 | 2.86499900 |
| H | 5.83340600 | -1.88045600 | 1.39590100 |
| H | 4.96781800 | -4.83031100 | -0.90921100 |
| H | 5.53054900 | -3.15407100 | -0.95575700 |
| H | 6.46755300 | -4.24908900 | 1.06412400 |
| H | 4.86211000 | -4.78793800 | 1.55702900 |
| N | 3.31706700 | -2.08971600 | 0.25865400 |
| N | -2.69740700 | 2.80095000 | 0.00417100 |
| N | -6.83836800 | -2.00951100 | -0.94212900 |
| H | -1.33346900 | 1.48973600 | -1.57069600 |

3.2. Acetonitrile

| **1a** |  | | |
| --- | --- | --- | --- |
|  | X | Y | Z |
| C | -1.23652600 | -1.97866200 | -0.05519200 |
| H | -1.49515300 | -3.02584400 | -0.16637300 |
| C | -2.22062000 | -0.97200600 | -0.05533400 |
| N | -0.49788000 | 0.72831400 | 0.23239300 |
| C | 0.08811200 | -1.59339900 | 0.11492200 |
| C | 1.34247300 | -2.38435900 | 0.17663500 |
| C | 0.39756000 | -0.25273700 | 0.27585300 |
| C | 1.89485600 | -0.07795300 | 0.46823400 |
| H | 2.32161100 | 0.49527800 | -0.36578600 |
| N | 2.35369300 | -1.47934400 | 0.39916300 |

| O | 1.47736100 | -3.60559200 | 0.05609400 |
| --- | --- | --- | --- |
| C | 3.76335000 | -1.85069700 | 0.44789400 |
| H | 4.22246900 | -1.42939300 | 1.34853300 |
| H | 3.79093000 | -2.94143300 | 0.53336900 |
| C | 4.55240500 | -1.39114600 | -0.73696900 |
| C | 5.62324500 | -0.55532500 | -0.87852400 |
| H | 6.11589200 | -0.01359400 | -0.08109600 |
| C | 5.95649400 | -0.54375700 | -2.27705700 |
| H | 6.75264700 | 0.00903900 | -2.75888900 |
| C | 5.06036800 | -1.36947400 | -2.88634600 |
| H | 4.89965300 | -1.67650500 | -3.91012300 |
| O | 4.19886100 | -1.89664700 | -1.96332800 |
| C | 2.28132100 | 0.60997500 | 1.74616700 |
| C | 2.09527200 | 0.33031800 | 3.07043200 |
| H | 1.59059400 | -0.53782600 | 3.47488600 |
| C | 2.69708300 | 1.40717600 | 3.80449000 |
| H | 2.74272900 | 1.52273000 | 4.87974300 |
| C | 3.20302500 | 2.26673700 | 2.87494600 |
| H | 3.73589000 | 3.20509200 | 2.93255700 |
| O | 2.96142900 | 1.79425300 | 1.61551600 |
| C | -1.78440700 | 0.39366000 | 0.04110100 |
| C | -2.77532100 | 1.53765800 | -0.12600800 |
| H | -3.56198200 | 1.19717800 | -0.80870900 |
| H | -3.27012600 | 1.73638000 | 0.83339200 |
| C | -2.17709600 | 2.82799700 | -0.64977200 |
| C | -1.89428100 | 2.97839400 | -2.01706800 |
| C | -1.91414600 | 3.90517400 | 0.20887800 |
| C | -1.35520500 | 4.16962300 | -2.51195200 |
| H | -2.10047500 | 2.15586000 | -2.69956300 |
| C | -1.37591300 | 5.10071500 | -0.28120100 |
| H | -2.13396700 | 3.80841600 | 1.27030400 |
| C | -1.09263500 | 5.23647300 | -1.64411800 |
| H | -1.14434600 | 4.26649400 | -3.57460400 |
| H | -1.18030600 | 5.92440700 | 0.40160900 |
| H | -0.67654600 | 6.16491500 | -2.02795300 |
| N | -3.58084500 | -1.27474400 | -0.20922800 |
| C | -3.93851100 | -2.59214300 | -0.75209400 |
| H | -3.63164900 | -3.41959500 | -0.09269100 |
| H | -3.43527500 | -2.73225700 | -1.71535000 |
| C | -5.44388600 | -2.65861900 | -0.96615600 |
| H | -5.76033000 | -1.86534800 | -1.65856100 |
| H | -5.71321100 | -3.62571400 | -1.40309800 |
| C | -4.55246900 | -0.76504300 | 0.79315700 |
| H | -4.01952100 | -0.33110900 | 1.64459400 |
| H | -5.18515600 | 0.01722600 | 0.35571500 |
| C | -5.43652300 | -1.89394400 | 1.31284700 |
| H | -4.83099700 | -2.63922200 | 1.84834800 |

| H | -6.17322100 | -1.48871200 | 2.01343100 |
| --- | --- | --- | --- |
| O | -6.17928500 | -2.52999600 | 0.26464800 |
| **1b** | X | Y | Z |
| C | 1.09631400 | -1.98593500 | 0.03431600 |
| C | 2.21249200 | -1.12909400 | -0.00562700 |
| H | 1.20598000 | -3.06319100 | -0.02261800 |
| N | 0.74548700 | 0.80810300 | 0.18410900 |
| C | -0.16109500 | -1.41138000 | 0.17585100 |
| C | -1.51353900 | -2.01616000 | 0.27914400 |
| C | -0.27861200 | -0.03481200 | 0.27101700 |
| C | -1.73622000 | 0.35367100 | 0.44895000 |
| N | -2.39095100 | -0.97146900 | 0.43247600 |
| O | -1.80541100 | -3.21664900 | 0.24629100 |
| C | -3.83242700 | -1.13763500 | 0.54020000 |
| H | -4.02131900 | -2.20789500 | 0.65710900 |
| H | -4.18548000 | -0.62726000 | 1.44661900 |
| C | -4.62057100 | -0.59860300 | -0.65860600 |
| H | -4.37875800 | 0.46144200 | -0.80845800 |
| C | -6.14209200 | -0.78942400 | -0.49217600 |
| H | -6.34769900 | -1.61535400 | 0.20141000 |
| H | -6.62508100 | 0.11029100 | -0.09881500 |
| C | -6.60556200 | -1.15912100 | -1.91009600 |
| H | -7.53188300 | -1.74304000 | -1.92068300 |
| H | -6.75667500 | -0.25765200 | -2.51675400 |
| C | -5.40547600 | -1.94917700 | -2.42719400 |
| H | -5.30076400 | -1.94494400 | -3.51690100 |
| H | -5.43723000 | -2.99394700 | -2.08161200 |
| O | -4.25088500 | -1.28736400 | -1.87530100 |
| C | -2.02093800 | 1.14389600 | 1.69411900 |
| C | -1.80830300 | 0.93117400 | 3.02693400 |
| H | -1.34722900 | 0.05669100 | 3.46813900 |
| C | -2.31371800 | 2.08670300 | 3.71231900 |
| H | -2.31414300 | 2.26843400 | 4.77938600 |
| C | -2.79447200 | 2.92236200 | 2.74818700 |
| H | -3.25961800 | 3.89759500 | 2.76460900 |
| O | -2.62624600 | 2.36229100 | 1.51317100 |
| C | 1.97241900 | 0.28661900 | 0.01880600 |
| C | 3.11395900 | 1.27256700 | -0.19176500 |
| H | 3.86376700 | 0.78465600 | -0.82431600 |
| C | 4.54245300 | -1.20902500 | 0.86558400 |
| C | 3.68735800 | -3.01093300 | -0.59184100 |
| C | 5.25847000 | -2.42128800 | 1.45152100 |
| H | 4.06785300 | -0.66346000 | 1.68680400 |
| H | 5.28131900 | -0.54386800 | 0.40138900 |
| C | 5.16877300 | -3.30273000 | -0.78356300 |

| H | 3.26245300 | -3.74886900 | 0.10724800 |
| --- | --- | --- | --- |
| H | 3.17369100 | -3.13238700 | -1.55208200 |
| H | 4.55341600 | -3.04782900 | 2.01679100 |
| H | 6.04024900 | -2.08559800 | 2.13998000 |
| H | 5.29882500 | -4.32274800 | -1.15989900 |
| H | 5.59731800 | -2.60553300 | -1.51771400 |
| N | 3.51786900 | -1.62785800 | -0.12665700 |
| O | 5.91210300 | -3.20566700 | 0.44571100 |
| H | 3.60694600 | 1.47155900 | 0.76866900 |
| C | 2.70919600 | 2.59063200 | -0.82177400 |
| C | 2.48551600 | 2.67900800 | -2.20548000 |
| C | 2.57398300 | 3.75201000 | -0.04778500 |
| C | 2.12802200 | 3.89337200 | -2.79878300 |
| H | 2.59504600 | 1.78920800 | -2.82284600 |
| C | 2.21709000 | 4.97079700 | -0.63649000 |
| H | 2.75116100 | 3.70256300 | 1.02479900 |
| C | 1.99123000 | 5.04529400 | -2.01480100 |
| H | 1.96104900 | 3.94135300 | -3.87252600 |
| H | 2.11809900 | 5.86038000 | -0.01852900 |
| H | 1.71660900 | 5.99144900 | -2.47510200 |
| H | -2.07672500 | 0.94890800 | -0.40837900 |
| **1c** | X | Y | Z |
| C | 1.03512500 | -1.99224800 | 0.07078500 |
| C | 2.16107800 | -1.15087400 | -0.00010000 |
| H | 1.13086600 | -3.07192900 | 0.03548900 |
| N | 0.72033700 | 0.80857300 | 0.16076600 |
| C | -0.21390800 | -1.39868300 | 0.21075500 |
| C | -1.57273100 | -1.98308400 | 0.33228400 |
| C | -0.31468700 | -0.01839200 | 0.27465400 |
| C | -1.76689700 | 0.39086000 | 0.45564200 |
| N | -2.43532900 | -0.92396700 | 0.47941800 |
| O | -1.88125500 | -3.18015000 | 0.31519700 |
| C | -3.88264400 | -1.07700600 | 0.52732500 |
| H | -4.08339600 | -2.14541400 | 0.64110300 |
| H | -4.27250500 | -0.55944100 | 1.41331000 |
| C | -4.61426100 | -0.53952900 | -0.70765800 |
| H | -4.37081300 | 0.52190900 | -0.84453000 |
| C | -6.14147300 | -0.73604900 | -0.61565800 |
| H | -6.37804200 | -1.56296200 | 0.06675700 |
| H | -6.64657500 | 0.16171900 | -0.24641200 |
| C | -6.53384100 | -1.10669800 | -2.05466600 |
| H | -7.45652900 | -1.69375700 | -2.11043100 |
| H | -6.65818500 | -0.20562400 | -2.66791200 |
| C | -5.30701900 | -1.89196000 | -2.51278100 |
| H | -5.14953000 | -1.88769300 | -3.59611100 |

| H | -5.35084300 | -2.93672000 | -2.16847900 |
| --- | --- | --- | --- |
| O | -4.18350900 | -1.22463500 | -1.90645500 |
| C | -2.01895900 | 1.22482000 | 1.68951800 |
| C | -2.22619600 | 0.79343700 | 2.96834100 |
| H | -2.29901100 | -0.18357500 | 3.42401600 |
| C | -2.26459300 | 2.99157500 | 3.05281500 |
| H | -2.36537800 | 3.92797700 | 3.58270900 |
| C | 1.93915500 | 0.26747400 | -0.00246100 |
| C | 3.09298400 | 1.23230700 | -0.24190200 |
| H | 3.82216300 | 0.72583400 | -0.88430100 |
| C | 4.49741300 | -1.25070900 | 0.85107000 |
| C | 3.60763900 | -3.05758000 | -0.57902400 |
| C | 5.20944100 | -2.46463300 | 1.43862600 |
| H | 4.03534900 | -0.69552900 | 1.67323600 |
| H | 5.23711900 | -0.59477200 | 0.37510500 |
| C | 5.08337200 | -3.36703400 | -0.78665200 |
| H | 3.18414400 | -3.78356500 | 0.13339600 |
| H | 3.07991300 | -3.18387000 | -1.53097300 |
| H | 4.50588900 | -3.07962700 | 2.01824400 |
| H | 6.00277100 | -2.13035800 | 2.11443200 |
| H | 5.19853700 | -4.39225800 | -1.15369500 |
| H | 5.50947900 | -2.68191500 | -1.53349600 |
| N | 3.45971100 | -1.66767600 | -0.12797600 |
| O | 5.84334200 | -3.26416000 | 0.43203000 |
| H | 3.60775700 | 1.42888300 | 0.70745800 |
| C | 2.70098400 | 2.55345100 | -0.87312100 |
| C | 2.45180200 | 2.63772100 | -2.25272000 |
| C | 2.60429800 | 3.72233200 | -0.10457300 |
| C | 2.10675100 | 3.85519400 | -2.84702700 |
| H | 2.53160300 | 1.74217300 | -2.86631000 |
| C | 2.26063000 | 4.94440100 | -0.69436500 |
| H | 2.80182500 | 3.67632900 | 0.96461100 |
| C | 2.00891300 | 5.01472600 | -2.06841200 |
| H | 1.91948900 | 3.89969000 | -3.91757200 |
| H | 2.19255400 | 5.83985700 | -0.08068000 |
| H | 1.74457700 | 5.96335300 | -2.52967800 |
| C | -2.04575300 | 2.66412400 | 1.74943900 |
| H | -1.91856900 | 3.35171200 | 0.92293200 |
| O | -2.37953500 | 1.85920600 | 3.81088600 |
| H | -2.09977500 | 0.96132400 | -0.42174700 |
| **1d** | X | Y | Z |
| N | 2.33769100 | -1.25784200 | 0.33912400 |
| C | 1.80775400 | 0.11451100 | 0.50613600 |
| C | 0.32987300 | -0.11666700 | 0.24665900 |
| C | 0.09496300 | -1.45092400 | -0.04969200 |

| C | 1.38506600 | -2.18295700 | -0.00964700 |
| --- | --- | --- | --- |
| N | -0.61551600 | 0.81704000 | 0.25808900 |
| C | -1.87580000 | 0.43918100 | -0.01731600 |
| C | -2.23780200 | -0.93006900 | -0.25847700 |
| C | -1.20138100 | -1.88184100 | -0.30238700 |
| O | 1.58902900 | -3.38027800 | -0.23797200 |
| C | 3.76075400 | -1.57905800 | 0.42459600 |
| C | 4.52769700 | -1.32447700 | -0.83493900 |
| C | 2.12578900 | 0.70992300 | 1.85082200 |
| N | -3.57785900 | -1.26753000 | -0.51961700 |
| C | -2.91109700 | 1.55235400 | -0.09818100 |
| C | -2.38055800 | 2.86802500 | -0.63472700 |
| C | -2.14099800 | 3.95413600 | 0.21930100 |
| C | -1.66321700 | 5.17097700 | -0.28113900 |
| C | -1.41866200 | 5.31932800 | -1.65013500 |
| C | -1.65917300 | 4.24361600 | -2.51361600 |
| C | -2.13761000 | 3.03101400 | -2.00845600 |
| C | -3.90007200 | -2.46655300 | -1.30525600 |
| C | -4.23055900 | -3.68353600 | -0.43773500 |
| O | -5.27068300 | -3.37432700 | 0.49782400 |
| C | -4.89549600 | -2.27360100 | 1.33177300 |
| C | -4.60894900 | -1.01874200 | 0.50584000 |
| C | 5.04999300 | -2.14052200 | -1.79790100 |
| C | 5.68716100 | -1.28756400 | -2.76348900 |
| C | 5.50657400 | -0.01182500 | -2.32053700 |
| O | 4.80509900 | -0.01431700 | -1.14585900 |
| C | 2.80671400 | 1.87065600 | 2.07440400 |
| O | 2.90531000 | 2.11733100 | 3.41383200 |
| C | 2.27643100 | 1.08456200 | 4.05736600 |
| C | 1.78115200 | 0.19592800 | 3.15437800 |
| H | 2.22416700 | 0.76617600 | -0.27326100 |
| H | -1.40160100 | -2.92756900 | -0.50886900 |
| H | 4.18846300 | -1.00395200 | 1.25290000 |
| H | 3.84366900 | -2.64004700 | 0.67145500 |
| H | -3.73441200 | 1.20454100 | -0.73123900 |
| H | -3.33915500 | 1.72399200 | 0.89852700 |
| H | -2.33008200 | 3.84684700 | 1.28560800 |
| H | -1.48385800 | 6.00081300 | 0.39868300 |
| H | -1.04916700 | 6.26406400 | -2.04178500 |
| H | -1.47836800 | 4.35025400 | -3.58086000 |
| H | -2.32636000 | 2.20124700 | -2.68721700 |
| H | -3.08017400 | -2.69458500 | -1.99171500 |
| H | -4.78460000 | -2.22732900 | -1.91282400 |
| H | -3.34381300 | -4.03632900 | 0.11040600 |
| H | -4.60616100 | -4.50199300 | -1.05971700 |
| H | -5.73772300 | -2.09528300 | 2.00799600 |
| H | -4.01278400 | -2.54886800 | 1.93137900 |

| H | -5.53119500 | -0.69949300 | 0.00134500 |
| --- | --- | --- | --- |
| H | -4.29345100 | -0.21920600 | 1.17742300 |
| H | 4.98813200 | -3.22111700 | -1.81644400 |
| H | 6.20957400 | -1.58788500 | -3.66271600 |
| H | 5.79792500 | 0.95981800 | -2.69368000 |
| H | 3.25928200 | 2.60100000 | 1.41816500 |
| H | 2.27170200 | 1.13448700 | 5.13696200 |
| H | 1.23845000 | -0.71376500 | 3.37782800 |
| **1e** | X | Y | Z |
| N | 2.24037600 | -1.40422900 | 0.47823600 |
| C | 1.76622700 | -0.01496100 | 0.65928600 |
| C | 0.28549200 | -0.17268600 | 0.36234800 |
| C | -0.01421900 | -1.50534100 | 0.11554000 |
| C | 1.24113500 | -2.29046000 | 0.15457300 |
| N | -0.61952500 | 0.80180000 | 0.31104300 |
| C | -1.89139600 | 0.46954700 | 0.03138900 |
| C | -2.31815500 | -0.89047300 | -0.14086700 |
| C | -1.32622900 | -1.88833700 | -0.13259600 |
| O | 1.39061500 | -3.49617200 | -0.07388600 |
| C | 3.65473100 | -1.78213000 | 0.37529100 |
| C | 4.23130800 | -1.61714500 | -0.99648300 |
| C | 2.02858900 | 0.53260600 | 2.07376900 |
| N | -3.67333000 | -1.17433700 | -0.38981000 |
| C | -2.86442300 | 1.62805200 | -0.13731900 |
| C | -2.26153300 | 2.85863500 | -0.78843200 |
| C | -2.02385400 | 4.02610100 | -0.04953700 |
| C | -1.47925900 | 5.16383000 | -0.65728400 |
| C | -1.16321500 | 5.14866900 | -2.01924400 |
| C | -1.40049600 | 3.98975600 | -2.76879000 |
| C | -1.94610900 | 2.85746200 | -2.15748700 |
| C | -4.05395100 | -2.40356400 | -1.10019700 |
| C | -4.42824400 | -3.55255800 | -0.16043900 |
| O | -5.44423700 | -3.14385600 | 0.76344600 |
| C | -5.01227500 | -2.01379900 | 1.52777800 |
| C | -4.68458200 | -0.82305000 | 0.62656500 |
| C | 4.61449700 | -2.49941700 | -1.96672100 |
| C | 5.11273200 | -1.71843500 | -3.06584000 |
| C | 4.99539400 | -0.41465600 | -2.68834300 |
| O | 4.46368800 | -0.33290000 | -1.43031200 |
| O | 3.44292900 | 0.55066700 | 2.32104700 |
| C | 3.63389000 | 1.40997500 | 3.45554300 |
| C | 2.65773600 | 2.57954000 | 3.24870600 |
| C | 1.57022900 | 1.98990600 | 2.30992600 |
| H | 2.25433300 | 0.64451900 | -0.06998400 |
| H | -1.56934900 | -2.93193900 | -0.30011900 |

| H | 4.21243200 | -1.19059500 | 1.10283800 |
| --- | --- | --- | --- |
| H | 3.73357600 | -2.83543200 | 0.65408700 |
| H | 1.55266500 | -0.14215700 | 2.80343600 |
| H | -3.71169700 | 1.27688400 | -0.73579100 |
| H | -3.27256200 | 1.90782300 | 0.84327500 |
| H | -2.26616200 | 4.04544100 | 1.01120000 |
| H | -1.30248600 | 6.05936700 | -0.06580700 |
| H | -0.74020400 | 6.03083700 | -2.49394400 |
| H | -1.16367900 | 3.96918600 | -3.83021100 |
| H | -2.13024600 | 1.96219400 | -2.74868200 |
| H | -3.25140200 | -2.70703100 | -1.77804800 |
| H | -4.93307200 | -2.16204900 | -1.71438800 |
| H | -3.55256100 | -3.91230500 | 0.40069500 |
| H | -4.84569600 | -4.38804200 | -0.73098700 |
| H | -5.83698400 | -1.76109200 | 2.20199400 |
| H | -4.13398400 | -2.29156900 | 2.13261000 |
| H | -5.59795500 | -0.49864500 | 0.10954400 |
| H | -4.33172800 | 0.00355100 | 1.24515200 |
| H | 4.54869000 | -3.57816900 | -1.90515800 |
| H | 5.50354200 | -2.08277900 | -4.00725200 |
| H | 5.22947300 | 0.52759800 | -3.16353500 |
| H | 4.68801000 | 1.69939300 | 3.47191700 |
| H | 3.40402000 | 0.85319100 | 4.37691000 |
| H | 2.24367000 | 2.92893200 | 4.19989200 |
| H | 3.16321500 | 3.42514700 | 2.77133900 |
| H | 0.57060500 | 2.02302300 | 2.75043000 |
| H | 1.53481900 | 2.53774900 | 1.36314500 |
| **1f** | X | Y | Z |
| N | 2.13430000 | -1.17643400 | 0.39144900 |
| C | 1.57026400 | 0.18609100 | 0.50040700 |
| C | 0.10183800 | -0.08461100 | 0.21628700 |
| C | -0.09752200 | -1.43274800 | -0.03828500 |
| C | 1.20672700 | -2.13452700 | 0.05525000 |
| N | -0.86053500 | 0.82983500 | 0.17102100 |
| C | -2.10564800 | 0.41609400 | -0.11993800 |
| C | -2.43449500 | -0.96882400 | -0.31783600 |
| C | -1.37864900 | -1.89984100 | -0.30378900 |
| O | 1.44110200 | -3.33311500 | -0.12450600 |
| C | 3.56270300 | -1.46392700 | 0.51106700 |
| C | 4.34715600 | -1.30948500 | -0.76959100 |
| C | 1.86441400 | 0.86454500 | 1.81283700 |
| N | -3.76058500 | -1.34295100 | -0.59516300 |
| C | -3.16000900 | 1.50417300 | -0.26705400 |
| C | -2.64225100 | 2.80415000 | -0.85215500 |
| C | -2.39287600 | 3.91754800 | -0.03692100 |

| C | -1.92292400 | 5.11759000 | -0.58292300 |
| --- | --- | --- | --- |
| C | -1.69651500 | 5.22160100 | -1.95925900 |
| C | -1.94765800 | 4.11863500 | -2.78422500 |
| C | -2.41822000 | 2.92272600 | -2.23352800 |
| C | -4.04133700 | -2.57625400 | -1.34321000 |
| C | -4.36990000 | -3.76720300 | -0.43967000 |
| O | -5.43590400 | -3.44384400 | 0.46111200 |
| C | -5.09876000 | -2.30706300 | 1.26253500 |
| C | -4.81899400 | -1.07718800 | 0.39785900 |
| C | 4.78521900 | -2.30475100 | -1.60902700 |
| C | 5.51480300 | -1.82091600 | -2.74081100 |
| C | 5.62848400 | -0.45579600 | -2.75355600 |
| S | 4.84770900 | 0.25756000 | -1.37534600 |
| S | 1.43570600 | 0.15377100 | 3.35676000 |
| C | 2.05502300 | 1.53066800 | 4.21798600 |
| C | 2.55703800 | 2.47754900 | 3.36581900 |
| C | 2.44837600 | 2.09371200 | 1.99153300 |
| H | 1.98446600 | 0.82735900 | -0.29007800 |
| H | -1.55329100 | -2.95622000 | -0.47605100 |
| H | 3.96895500 | -0.82148000 | 1.29865000 |
| H | 3.65711600 | -2.50000700 | 0.84639700 |
| H | -3.96496900 | 1.11440400 | -0.89889300 |
| H | -3.60804300 | 1.70976500 | 0.71448600 |
| H | -2.56748200 | 3.84439700 | 1.03468000 |
| H | -1.73520800 | 5.96928500 | 0.06697600 |
| H | -1.33274400 | 6.15323100 | -2.38606700 |
| H | -1.78135000 | 4.19066300 | -3.85671500 |
| H | -2.61563300 | 2.07142200 | -2.88250900 |
| H | -3.20112800 | -2.81291800 | -2.00149400 |
| H | -4.91525200 | -2.37602500 | -1.97931900 |
| H | -3.48956800 | -4.08470600 | 0.13962900 |
| H | -4.71687600 | -4.61395800 | -1.04003900 |
| H | -5.95790200 | -2.12140800 | 1.91506400 |
| H | -4.22338900 | -2.54407300 | 1.88850600 |
| H | -5.73578200 | -0.79542400 | -0.13790600 |
| H | -4.53396000 | -0.24704100 | 1.04559500 |
| H | 4.59385200 | -3.35643900 | -1.41812900 |
| H | 5.93692300 | -2.46261200 | -3.50804000 |
| H | 6.12261300 | 0.17460000 | -3.48339000 |
| H | 2.01820100 | 1.54894600 | 5.30091200 |
| H | 2.99454500 | 3.41267000 | 3.70097400 |
| H | 2.78970800 | 2.70511300 | 1.16135200 |
| **1g** | X | Y | Z |
| C | -0.10570200 | 0.23329300 | -0.35466500 |
| C | -0.24361500 | -1.13239900 | -0.55266600 |

| C | 0.84927300 | -1.96922000 | -0.35347300 |
| --- | --- | --- | --- |
| C | 2.06321200 | -1.38251700 | 0.04168000 |
| C | 2.11358500 | 0.04375500 | 0.16844800 |
| C | -1.42200500 | 0.93688900 | -0.61941500 |
| C | -1.63602800 | -1.40970700 | -0.98041100 |
| H | 0.75819400 | -3.04182500 | -0.48844400 |
| N | 1.03358200 | 0.82514700 | -0.00750100 |
| N | -2.30881700 | -0.21006100 | -0.91814500 |
| O | -2.12075900 | -2.48246900 | -1.35162300 |
| C | -1.91300200 | 1.81858100 | 0.52296000 |
| C | -1.99016600 | 1.34075800 | 1.83807400 |
| C | -2.44091400 | 2.19736900 | 2.84084900 |
| H | -1.70179600 | 0.31880500 | 2.06786900 |
| C | -2.68972100 | 3.89207800 | 1.16487500 |
| C | -2.80019400 | 3.50402000 | 2.49984600 |
| H | -2.50813500 | 1.85305800 | 3.86952800 |
| H | -2.95857900 | 4.90076900 | 0.85804400 |
| H | -3.15589800 | 4.20715700 | 3.24735600 |
| C | -3.61270700 | -0.02650500 | -1.55536100 |
| H | -3.80385000 | 1.05102200 | -1.57905800 |
| H | -3.55745700 | -0.36667200 | -2.59681100 |
| C | -4.76773700 | -0.72715300 | -0.86337800 |
| C | -4.95609600 | -0.69946300 | 0.52286300 |
| C | -5.73149300 | -1.39600700 | -1.62834200 |
| C | -6.07461000 | -1.32628500 | 1.07233900 |
| H | -4.23682100 | -0.19917700 | 1.16497100 |
| H | -5.61902600 | -1.45119000 | -2.71035400 |
| C | -6.97807800 | -1.95949600 | 0.21619200 |
| H | -6.24765900 | -1.32386800 | 2.14494800 |
| H | -7.86186500 | -2.45596400 | 0.61175700 |
| C | 3.41502100 | 0.76045400 | 0.49140700 |
| H | 3.43824600 | 0.98831900 | 1.56555400 |
| H | 4.24215600 | 0.07069900 | 0.29894300 |
| C | 3.63285600 | 2.04097700 | -0.29348500 |
| C | 3.53427600 | 3.29677200 | 0.32180800 |
| C | 3.96339000 | 1.98842100 | -1.65804200 |
| C | 3.75709100 | 4.47285700 | -0.40452400 |
| H | 3.28246000 | 3.35564000 | 1.37881600 |
| C | 4.18537000 | 3.15952400 | -2.38806700 |
| H | 4.05132200 | 1.02179900 | -2.15084100 |
| C | 4.08239200 | 4.40831100 | -1.76294300 |
| H | 3.67673200 | 5.43694800 | 0.09257700 |
| H | 4.44330000 | 3.09755900 | -3.44293200 |
| H | 4.25806000 | 5.32016000 | -2.32875100 |
| C | 3.65071700 | -2.23269700 | 1.69095300 |
| C | 3.29127900 | -3.46848100 | -0.38042300 |
| C | 5.08520300 | -2.75150200 | 1.80963300 |

| H | 2.96405100 | -2.90752100 | 2.23550500 |
| --- | --- | --- | --- |
| H | 3.56376100 | -1.24605200 | 2.15077800 |
| C | 4.72103800 | -4.01934800 | -0.33760600 |
| H | 2.61133500 | -4.18838600 | 0.11153400 |
| H | 2.96052000 | -3.35493400 | -1.41785800 |
| C | 5.24326700 | -4.10392100 | 1.10285200 |
| H | 5.34339600 | -2.83622700 | 2.87324300 |
| H | 5.77021100 | -2.01573000 | 1.36538300 |
| H | 4.73028700 | -5.00926500 | -0.81196900 |
| H | 5.37407200 | -3.36647800 | -0.93362100 |
| H | 6.29343500 | -4.42281700 | 1.11211500 |
| H | 4.67186500 | -4.86802500 | 1.65099400 |
| N | 3.22963500 | -2.15083900 | 0.27298600 |
| N | -2.25612900 | 3.07416400 | 0.19041400 |
| N | -6.81644300 | -1.99811900 | -1.11604000 |
| H | -1.34443800 | 1.57375700 | -1.51017800 |

3.3. Methanol

| **1a** |  | | |
| --- | --- | --- | --- |
|  | X | Y | Z |
| C | -1.19285100 | -1.94375500 | -0.02799000 |
| H | -1.42096800 | -2.99697100 | -0.14694500 |
| C | -2.19992700 | -0.96019700 | -0.03745100 |
| N | -0.51871500 | 0.77798800 | 0.27593700 |
| C | 0.11931100 | -1.52792000 | 0.16351600 |
| C | 1.38799200 | -2.28714900 | 0.22965900 |
| C | 0.39776200 | -0.18210600 | 0.33822500 |
| C | 1.88563400 | 0.02288700 | 0.54403000 |
| H | 2.31458100 | 0.58640100 | -0.29550400 |
| N | 2.37502400 | -1.37710600 | 0.48387100 |
| O | 1.54856000 | -3.51105400 | 0.08284700 |
| C | 3.79770100 | -1.71145800 | 0.50585600 |
| H | 4.27920300 | -1.17129700 | 1.32633200 |
| H | 3.87024400 | -2.78158500 | 0.72324300 |
| C | 4.51199500 | -1.38102900 | -0.76484900 |
| C | 5.51334100 | -0.50984400 | -1.08136000 |
| H | 5.99927200 | 0.17427000 | -0.39725100 |
| C | 5.78626800 | -0.68133700 | -2.48274400 |
| H | 6.52215400 | -0.15517400 | -3.07728400 |
| C | 4.92818500 | -1.64184900 | -2.92341900 |
| H | 4.74835800 | -2.10748400 | -3.88214900 |
| O | 4.14233000 | -2.08497000 | -1.88980500 |
| C | 2.24979200 | 0.73041900 | 1.81285100 |
| C | 1.77545200 | 0.70190100 | 3.09290900 |
| H | 0.94672300 | 0.10580400 | 3.45318800 |
| C | 2.58288200 | 1.61315100 | 3.85334900 |

| H | 2.49007500 | 1.84895700 | 4.90576500 |
| --- | --- | --- | --- |
| C | 3.49191600 | 2.13686600 | 2.98392500 |
| H | 4.29254000 | 2.85708300 | 3.07596500 |
| O | 3.30685000 | 1.60992600 | 1.73394000 |
| C | -1.79355200 | 0.41530000 | 0.05813100 |
| C | -2.79709500 | 1.54375700 | -0.13740100 |
| H | -3.59316200 | 1.17590500 | -0.79350400 |
| H | -3.27589500 | 1.77925600 | 0.82203300 |
| C | -2.21235300 | 2.81447400 | -0.72266400 |
| C | -1.91435000 | 2.89535500 | -2.09263600 |
| C | -1.97538400 | 3.93938100 | 0.08002500 |
| C | -1.38538400 | 4.06584800 | -2.64393000 |
| H | -2.09961400 | 2.03452600 | -2.73280500 |
| C | -1.44690900 | 5.11438700 | -0.46694700 |
| H | -2.20539100 | 3.89583800 | 1.14290300 |
| C | -1.14793700 | 5.18085800 | -1.83129200 |
| H | -1.16192500 | 4.10878800 | -3.70768600 |
| H | -1.27002300 | 5.97596300 | 0.17311500 |
| H | -0.73830300 | 6.09299100 | -2.25905500 |
| N | -3.55129000 | -1.29590300 | -0.20085200 |
| C | -3.86891900 | -2.64447300 | -0.69239200 |
| H | -3.52402200 | -3.43561100 | -0.00826000 |
| H | -3.37442200 | -2.80270900 | -1.65749000 |
| C | -5.37145400 | -2.77842900 | -0.88421200 |
| H | -5.73598300 | -2.02358000 | -1.59439000 |
| H | -5.60742000 | -3.77048200 | -1.28153000 |
| C | -4.54362500 | -0.78134700 | 0.77898800 |
| H | -4.03182900 | -0.27666200 | 1.60312800 |
| H | -5.21657000 | -0.05722200 | 0.30355000 |
| C | -5.36781300 | -1.91914200 | 1.36856200 |
| H | -4.72602900 | -2.61243000 | 1.92952400 |
| H | -6.11644500 | -1.51367400 | 2.05593900 |
| O | -6.09328300 | -2.64053000 | 0.35827300 |
| **1b** | X | Y | Z |
| C | 1.08669500 | -1.95649300 | 0.08433900 |
| C | 2.21032600 | -1.11038800 | 0.03287900 |
| H | 1.18173900 | -3.03523600 | 0.02844000 |
| N | 0.76272000 | 0.84189000 | 0.22699600 |
| C | -0.16330400 | -1.36787400 | 0.23325700 |
| C | -1.51885200 | -1.95549100 | 0.32876400 |
| C | -0.26868100 | 0.01004200 | 0.32822600 |
| C | -1.72056100 | 0.41111500 | 0.49217000 |
| N | -2.38742900 | -0.91581100 | 0.49218900 |
| O | -1.82232100 | -3.16140600 | 0.27635400 |
| C | -3.83441600 | -1.06617000 | 0.57491300 |

| H | -4.04102600 | -2.11722800 | 0.79372600 |
| --- | --- | --- | --- |
| H | -4.20494200 | -0.46588500 | 1.41509500 |
| C | -4.57329900 | -0.64497500 | -0.69736200 |
| H | -4.32596000 | 0.39640500 | -0.93989500 |
| C | -6.09728600 | -0.82840400 | -0.59474900 |
| H | -6.33744300 | -1.62257600 | 0.12417900 |
| H | -6.59639900 | 0.08892100 | -0.26792700 |
| C | -6.48884600 | -1.26156400 | -2.01571200 |
| H | -7.42795500 | -1.82357600 | -2.05024500 |
| H | -6.58082800 | -0.38986300 | -2.67527000 |
| C | -5.28633800 | -2.10785900 | -2.42249500 |
| H | -5.11231800 | -2.15256900 | -3.50213000 |
| H | -5.36529300 | -3.13311300 | -2.03327000 |
| O | -4.14255700 | -1.45512200 | -1.82265500 |
| C | -2.01461600 | 1.22896200 | 1.71177300 |
| C | -1.56142700 | 1.22539300 | 2.99963900 |
| H | -0.80441200 | 0.56813000 | 3.40811100 |
| C | -2.27890800 | 2.25739900 | 3.69330100 |
| H | -2.17507500 | 2.54213700 | 4.73250100 |
| C | -3.11845300 | 2.82071400 | 2.77994200 |
| H | -3.84082100 | 3.62388100 | 2.81689900 |
| O | -2.97218200 | 2.20818700 | 1.56428600 |
| C | 1.98270300 | 0.30808400 | 0.04947400 |
| C | 3.12381800 | 1.28816800 | -0.18655700 |
| H | 3.88044600 | 0.78611300 | -0.79840700 |
| C | 4.55022100 | -1.18984200 | 0.87300300 |
| C | 3.66118000 | -3.01836900 | -0.52918000 |
| C | 5.25883100 | -2.39114900 | 1.48644800 |
| H | 4.09311400 | -0.61787100 | 1.68602100 |
| H | 5.29215100 | -0.54675100 | 0.38384600 |
| C | 5.13594900 | -3.32906400 | -0.73268700 |
| H | 3.23786700 | -3.73619800 | 0.19123400 |
| H | 3.13679400 | -3.15866100 | -1.48102500 |
| H | 4.55725500 | -2.99804300 | 2.07528900 |
| H | 6.05409300 | -2.04579300 | 2.15392800 |
| H | 5.25422100 | -4.35829900 | -1.08579800 |
| H | 5.56706200 | -2.65150300 | -1.48232100 |
| N | 3.50920800 | -1.62276100 | -0.09553500 |
| O | 5.89287400 | -3.21277700 | 0.49165900 |
| H | 3.60958800 | 1.52203000 | 0.76983300 |
| C | 2.71462700 | 2.58028600 | -0.86645900 |
| C | 2.43264700 | 2.59904200 | -2.24230200 |
| C | 2.63059700 | 3.78283800 | -0.15089800 |
| C | 2.06677900 | 3.78567100 | -2.88356500 |
| H | 2.50173900 | 1.67638900 | -2.81602100 |
| C | 2.26587300 | 4.97453900 | -0.78854800 |
| H | 2.85249000 | 3.78832700 | 0.91454300 |

| C | 1.98046200 | 4.97958000 | -2.15733000 |
| --- | --- | --- | --- |
| H | 1.85339200 | 3.77964900 | -3.95026400 |
| H | 2.20675600 | 5.89722400 | -0.21543600 |
| H | 1.69874100 | 5.90428900 | -2.65567600 |
| H | -2.05731500 | 0.98024800 | -0.38437200 |
| **1c** | X | Y | Z |
| C | 1.03868600 | -1.95309900 | 0.07159500 |
| C | 2.17224200 | -1.12121300 | 0.01573800 |
| H | 1.12033400 | -3.03332500 | 0.02107400 |
| N | 0.74861300 | 0.84961200 | 0.20060400 |
| C | -0.20407800 | -1.34805100 | 0.21770900 |
| C | -1.56609500 | -1.91811000 | 0.31681800 |
| C | -0.29430900 | 0.03170500 | 0.30757600 |
| C | -1.73935900 | 0.45191800 | 0.48403900 |
| N | -2.42094900 | -0.86790400 | 0.48188800 |
| O | -1.88447000 | -3.12119100 | 0.26575200 |
| C | -3.86954000 | -1.00536000 | 0.55041400 |
| H | -4.08805500 | -2.05214900 | 0.77830100 |
| H | -4.24401800 | -0.39261400 | 1.37944400 |
| C | -4.59325800 | -0.59289300 | -0.73379900 |
| H | -4.33466000 | 0.44317400 | -0.98723000 |
| C | -6.11972800 | -0.76208400 | -0.64214300 |
| H | -6.37261600 | -1.54430000 | 0.08547600 |
| H | -6.61395800 | 0.16358900 | -0.33176000 |
| C | -6.50345700 | -1.21127300 | -2.06029400 |
| H | -7.44640700 | -1.76684300 | -2.09468300 |
| H | -6.58388300 | -0.34788100 | -2.73221700 |
| C | -5.30422600 | -2.07181600 | -2.44593100 |
| H | -5.12240800 | -2.13326400 | -3.52347000 |
| H | -5.39363500 | -3.09085100 | -2.04285700 |
| O | -4.16042400 | -1.41866600 | -1.84690800 |
| C | -2.02266000 | 1.27740800 | 1.71590700 |
| C | -1.50606600 | 1.10911200 | 2.96840300 |
| H | -0.80581600 | 0.40922100 | 3.40191900 |
| C | -2.86550100 | 2.84162600 | 3.09201400 |
| H | -3.34353000 | 3.64430300 | 3.63488800 |
| C | 1.96150100 | 0.29951200 | 0.02397600 |
| C | 3.11497200 | 1.26348500 | -0.21826900 |
| H | 3.86780200 | 0.74588600 | -0.82174900 |
| C | 4.50696900 | -1.22546800 | 0.86569900 |
| C | 3.60285000 | -3.04835300 | -0.53344900 |
| C | 5.19632800 | -2.43306100 | 1.48853900 |
| H | 4.05218800 | -0.64396200 | 1.67330200 |
| H | 5.26043900 | -0.59414600 | 0.37873900 |
| C | 5.07452900 | -3.37873800 | -0.72742100 |

| H | 3.16732900 | -3.75736600 | 0.18851600 |
| --- | --- | --- | --- |
| H | 3.08133600 | -3.18709300 | -1.48711600 |
| H | 4.48329500 | -3.02838400 | 2.07542900 |
| H | 5.99188600 | -2.09547500 | 2.15965300 |
| H | 5.18178600 | -4.41089300 | -1.07553900 |
| H | 5.51851800 | -2.70986400 | -1.47735100 |
| N | 3.46647900 | -1.64876100 | -0.10798800 |
| O | 5.82578100 | -3.26679400 | 0.50090200 |
| H | 3.60018600 | 1.50195700 | 0.73728400 |
| C | 2.72400200 | 2.55286200 | -0.91392100 |
| C | 2.43374100 | 2.55734900 | -2.28824400 |
| C | 2.66679400 | 3.76694400 | -0.21537400 |
| C | 2.08631000 | 3.74138400 | -2.94427500 |
| H | 2.48180400 | 1.62552600 | -2.84916800 |
| C | 2.32062100 | 4.95616100 | -0.86792700 |
| H | 2.89510700 | 3.78358400 | 0.84859900 |
| C | 2.02715000 | 4.94711300 | -2.23491400 |
| H | 1.86634000 | 3.72423700 | -4.00952800 |
| H | 2.28236900 | 5.88775500 | -0.30759700 |
| H | 1.76004500 | 5.86976200 | -2.74501900 |
| C | -2.90817300 | 2.41154600 | 1.80230200 |
| H | -3.49038500 | 2.84195300 | 0.99718700 |
| O | -2.01085400 | 2.05229200 | 3.82143800 |
| H | -2.07213500 | 1.02004600 | -0.39413100 |
| **1d** | X | Y | Z |
| N | 2.34694400 | -1.24445800 | 0.37230400 |
| C | 1.81211200 | 0.12776800 | 0.54405900 |
| C | 0.33652500 | -0.10609200 | 0.28199500 |
| C | 0.10773600 | -1.43980000 | -0.02126200 |
| C | 1.39928500 | -2.16001400 | 0.01652200 |
| N | -0.61255300 | 0.82335000 | 0.29088500 |
| C | -1.86902800 | 0.44131400 | 0.00162200 |
| C | -2.22359800 | -0.92807200 | -0.25037500 |
| C | -1.18416300 | -1.87590900 | -0.28687100 |
| O | 1.60865500 | -3.36202900 | -0.22773700 |
| C | 3.77739100 | -1.55090900 | 0.42323400 |
| C | 4.49794600 | -1.31651200 | -0.86570100 |
| C | 2.13195400 | 0.71880500 | 1.88906900 |
| N | -3.55994800 | -1.26917200 | -0.53195600 |
| C | -2.90410400 | 1.55384800 | -0.07578900 |
| C | -2.38011200 | 2.86226800 | -0.63662700 |
| C | -2.16352400 | 3.97095200 | 0.19367300 |
| C | -1.69579700 | 5.18152000 | -0.33090500 |
| C | -1.43795500 | 5.30032400 | -1.70001700 |
| C | -1.65527500 | 4.20135900 | -2.53994100 |

| C | -2.12350800 | 2.99525300 | -2.01109600 |
| --- | --- | --- | --- |
| C | -3.86308200 | -2.48529800 | -1.30044800 |
| C | -4.17613400 | -3.69806600 | -0.42200600 |
| O | -5.22555600 | -3.39128900 | 0.51036100 |
| C | -4.86738800 | -2.27069000 | 1.33260900 |
| C | -4.59984600 | -1.02640900 | 0.48727200 |
| C | 4.94274500 | -2.14112000 | -1.85830300 |
| C | 5.55805800 | -1.30383400 | -2.85148300 |
| C | 5.44461900 | -0.02552300 | -2.39645300 |
| O | 4.80353200 | -0.01039200 | -1.18434800 |
| C | 2.83070400 | 1.86771100 | 2.11261500 |
| O | 2.92718700 | 2.11423000 | 3.45594800 |
| C | 2.27505100 | 1.08880900 | 4.09787500 |
| C | 1.77080300 | 0.21114600 | 3.19106600 |
| H | 2.23077400 | 0.77730300 | -0.23562800 |
| H | -1.37417100 | -2.92175500 | -0.50138300 |
| H | 4.22103100 | -0.94820800 | 1.22226000 |
| H | 3.88301800 | -2.60296200 | 0.69803500 |
| H | -3.74004900 | 1.20468100 | -0.69050900 |
| H | -3.31262000 | 1.73732700 | 0.92716600 |
| H | -2.36247100 | 3.88708600 | 1.26039700 |
| H | -1.53384600 | 6.02948200 | 0.33088400 |
| H | -1.07581700 | 6.24021100 | -2.11009500 |
| H | -1.46346400 | 4.28454900 | -3.60747100 |
| H | -2.29405700 | 2.14731400 | -2.67214700 |
| H | -3.04087400 | -2.71028800 | -1.98518100 |
| H | -4.74961000 | -2.26699600 | -1.91233900 |
| H | -3.29096000 | -4.03499400 | 0.13635000 |
| H | -4.54101400 | -4.52738300 | -1.03552600 |
| H | -5.71420300 | -2.09660100 | 2.00372300 |
| H | -3.98312600 | -2.52976200 | 1.93534700 |
| H | -5.52585200 | -0.73191300 | -0.02478900 |
| H | -4.30516900 | -0.21136100 | 1.14937900 |
| H | 4.84284800 | -3.21882600 | -1.87982400 |
| H | 6.02285100 | -1.61735900 | -3.77753000 |
| H | 5.75040800 | 0.93848700 | -2.77776500 |
| H | 3.29845600 | 2.59147400 | 1.45989800 |
| H | 2.26643100 | 1.14065800 | 5.17727700 |
| H | 1.21136500 | -0.68933000 | 3.41104600 |
| **1e** | X | Y | Z |
| N | 2.29349400 | -1.31008800 | 0.57753900 |
| C | 1.79048000 | 0.08053300 | 0.66531700 |
| C | 0.31163400 | -0.12797000 | 0.39219100 |
| C | 0.04425300 | -1.47860500 | 0.21637500 |
| C | 1.31551800 | -2.22351100 | 0.29893000 |

| N | -0.61827900 | 0.81889100 | 0.29653400 |
| --- | --- | --- | --- |
| C | -1.88086100 | 0.44003600 | 0.03376500 |
| C | -2.27094200 | -0.93717900 | -0.08327200 |
| C | -1.25587100 | -1.90794600 | -0.01903700 |
| O | 1.49546000 | -3.44284600 | 0.12703100 |
| C | 3.71643300 | -1.66257800 | 0.45958600 |
| C | 4.22050600 | -1.66582000 | -0.94811500 |
| C | 2.05409900 | 0.73717700 | 2.03100700 |
| N | -3.61436000 | -1.26689700 | -0.34282900 |
| C | -2.88786200 | 1.56261600 | -0.16693000 |
| C | -2.32835200 | 2.79712000 | -0.84764600 |
| C | -2.13218700 | 3.98949400 | -0.13712200 |
| C | -1.63574300 | 5.13292800 | -0.77427100 |
| C | -1.32722400 | 5.09867800 | -2.13740200 |
| C | -1.52215200 | 3.91426900 | -2.85841200 |
| C | -2.01969200 | 2.77611500 | -2.21788200 |
| C | -3.94702200 | -2.53208300 | -1.01358300 |
| C | -4.30886500 | -3.65884900 | -0.04428000 |
| O | -5.35509400 | -3.24162300 | 0.84741100 |
| C | -4.96312400 | -2.07341700 | 1.58326400 |
| C | -4.65021600 | -0.90882600 | 0.64580600 |
| C | 4.52990500 | -2.65684600 | -1.83439400 |
| C | 4.96406800 | -2.01424600 | -3.04474300 |
| C | 4.88637800 | -0.67455500 | -2.81425100 |
| O | 4.43843200 | -0.43927100 | -1.53963700 |
| O | 3.47288200 | 0.80624700 | 2.26408800 |
| C | 3.65263800 | 1.75980600 | 3.32848100 |
| C | 2.63937500 | 2.88000100 | 3.04676800 |
| C | 1.56772300 | 2.19742700 | 2.15494300 |
| H | 2.25962600 | 0.69016800 | -0.11788400 |
| H | -1.46908600 | -2.96485700 | -0.13669300 |
| H | 4.28484900 | -0.96223400 | 1.07214400 |
| H | 3.84785000 | -2.66374000 | 0.87711100 |
| H | 1.60297200 | 0.11219000 | 2.81717400 |
| H | -3.72453800 | 1.17197000 | -0.75548000 |
| H | -3.30408700 | 1.85188000 | 0.80768200 |
| H | -2.37006800 | 4.02509700 | 0.92430400 |
| H | -1.49140400 | 6.04834800 | -0.20459300 |
| H | -0.94241900 | 5.98581100 | -2.63509100 |
| H | -1.29046100 | 3.87857200 | -3.92069400 |
| H | -2.17222700 | 1.86119900 | -2.78786400 |
| H | -3.12421300 | -2.83612500 | -1.66646500 |
| H | -4.81947500 | -2.33767600 | -1.65326900 |
| H | -3.44065100 | -3.97859300 | 0.54988800 |
| H | -4.69687900 | -4.52243200 | -0.59288500 |
| H | -5.80604400 | -1.82045000 | 2.23409200 |
| H | -4.09026800 | -2.31499100 | 2.20932500 |

| H | -5.56367600 | -0.62403600 | 0.10617300 |
| --- | --- | --- | --- |
| H | -4.32957000 | -0.05384200 | 1.24292500 |
| H | 4.45573700 | -3.72035000 | -1.64666800 |
| H | 5.28977800 | -2.49077700 | -3.96060100 |
| H | 5.10214800 | 0.20395200 | -3.40589100 |
| H | 4.69790000 | 2.07873900 | 3.30479400 |
| H | 3.45276600 | 1.26595600 | 4.29092600 |
| H | 2.21706500 | 3.27682600 | 3.97547600 |
| H | 3.11580100 | 3.70915300 | 2.51385900 |
| H | 0.56615000 | 2.24435000 | 2.58912000 |
| H | 1.52559200 | 2.66987000 | 1.16866700 |
| **1f** | X | Y | Z |
| N | 2.16934500 | -1.10624700 | 0.45622100 |
| C | 1.58097000 | 0.24917700 | 0.56081200 |
| C | 0.11859800 | -0.04762500 | 0.27772500 |
| C | -0.05519400 | -1.39992800 | 0.02592700 |
| C | 1.25972500 | -2.07073100 | 0.12303100 |
| N | -0.86039300 | 0.84789600 | 0.22384000 |
| C | -2.09444300 | 0.40935200 | -0.08135000 |
| C | -2.39525300 | -0.98137300 | -0.28140300 |
| C | -1.32406000 | -1.89317300 | -0.24987300 |
| O | 1.51619100 | -3.27249700 | -0.06215500 |
| C | 3.60960800 | -1.36112000 | 0.53924900 |
| C | 4.32819700 | -1.28414200 | -0.78496100 |
| C | 1.86828000 | 0.93736400 | 1.86828000 |
| N | -3.71084600 | -1.37988200 | -0.58029800 |
| C | -3.16097400 | 1.48265000 | -0.23626500 |
| C | -2.66929600 | 2.76032700 | -0.89065400 |
| C | -2.52499000 | 3.94389800 | -0.15379900 |
| C | -2.08957500 | 5.12420700 | -0.76813000 |
| C | -1.79122100 | 5.13595800 | -2.13393400 |
| C | -1.93533700 | 3.96047000 | -2.88104700 |
| C | -2.37195000 | 2.78539500 | -2.26332000 |
| C | -3.95457000 | -2.63326700 | -1.30938200 |
| C | -4.25860200 | -3.82036400 | -0.39400900 |
| O | -5.34331900 | -3.50824600 | 0.49498200 |
| C | -5.03783400 | -2.34970200 | 1.28554500 |
| C | -4.78340200 | -1.12913700 | 0.40255900 |
| C | 4.66202100 | -2.32385000 | -1.61832300 |
| C | 5.32619500 | -1.90728400 | -2.81460600 |
| C | 5.49345500 | -0.54933400 | -2.88211100 |
| S | 4.84600200 | 0.24045800 | -1.47757200 |
| S | 1.42270500 | 0.24544800 | 3.41575300 |
| C | 2.04235900 | 1.62799000 | 4.26677800 |
| C | 2.55586400 | 2.56331800 | 3.40892100 |

| C | 2.45620100 | 2.16581000 | 2.03830800 |
| --- | --- | --- | --- |
| H | 1.98622900 | 0.88894200 | -0.23509700 |
| H | -1.47371800 | -2.95343300 | -0.42115100 |
| H | 4.02942700 | -0.65367700 | 1.26051700 |
| H | 3.74290400 | -2.36556600 | 0.95029500 |
| H | -3.98581400 | 1.07131200 | -0.82619700 |
| H | -3.57559100 | 1.72373300 | 0.75194600 |
| H | -2.75579500 | 3.94340400 | 0.90972900 |
| H | -1.98470800 | 6.03244800 | -0.17871000 |
| H | -1.45391600 | 6.05181600 | -2.61372300 |
| H | -1.71150100 | 3.96022700 | -3.94560300 |
| H | -2.48574900 | 1.87738600 | -2.85302700 |
| H | -3.10624000 | -2.85842500 | -1.96132500 |
| H | -4.82865500 | -2.46534200 | -1.95406200 |
| H | -3.38110700 | -4.11005200 | 0.20188900 |
| H | -4.58139200 | -4.68272600 | -0.98521400 |
| H | -5.90595300 | -2.17677900 | 1.92914400 |
| H | -4.16196300 | -2.56285900 | 1.91769600 |
| H | -5.70371100 | -0.87941700 | -0.14257500 |
| H | -4.52749100 | -0.28209500 | 1.04064100 |
| H | 4.43883500 | -3.35953600 | -1.38020900 |
| H | 5.66640700 | -2.58979800 | -3.58742300 |
| H | 5.96312400 | 0.03474200 | -3.66492600 |
| H | 1.99728500 | 1.65781800 | 5.34924900 |
| H | 2.99516900 | 3.49994700 | 3.73777500 |
| H | 2.80624400 | 2.76771100 | 1.20470900 |
| **1g** | X | Y | Z |
| C | -0.10875200 | 0.25095500 | -0.37432500 |
| C | -0.24936600 | -1.11404900 | -0.57427100 |
| C | 0.83552500 | -1.95761400 | -0.36193400 |
| C | 2.04681500 | -1.37702300 | 0.04932400 |
| C | 2.10186700 | 0.04907200 | 0.17810300 |
| C | -1.41934500 | 0.95806100 | -0.65625300 |
| C | -1.63412100 | -1.38050100 | -1.01747000 |
| H | 0.73704600 | -3.02915200 | -0.49981200 |
| N | 1.02806300 | 0.83681300 | -0.01173600 |
| N | -2.30081100 | -0.18687400 | -0.98064600 |
| O | -2.12151500 | -2.46206100 | -1.38847200 |
| C | -1.92172500 | 1.82921200 | 0.48817100 |
| C | -2.06046900 | 1.32164000 | 1.78628800 |
| C | -2.50323400 | 2.17162900 | 2.79784900 |
| H | -1.82613200 | 0.28156500 | 1.99612200 |
| C | -2.62556900 | 3.91729500 | 1.16139300 |
| C | -2.79341200 | 3.50133700 | 2.48122700 |
| H | -2.61756900 | 1.80433200 | 3.81449200 |

| H | -2.84097700 | 4.94436300 | 0.87393400 |
| --- | --- | --- | --- |
| H | -3.14066500 | 4.20087700 | 3.23622200 |
| C | -3.61623600 | -0.00951600 | -1.60726600 |
| H | -3.80354200 | 1.06698400 | -1.64887300 |
| H | -3.56481000 | -0.37199300 | -2.64101200 |
| C | -4.75889000 | -0.70133500 | -0.88659600 |
| C | -5.15367000 | -0.34757200 | 0.40924300 |
| C | -5.49079800 | -1.70683000 | -1.52826200 |
| C | -6.23216900 | -1.00708200 | 0.99759700 |
| H | -4.62592000 | 0.43252900 | 0.95151200 |
| H | -5.22096900 | -2.01184700 | -2.53795200 |
| C | -6.89055400 | -1.99834900 | 0.26749400 |
| H | -6.56271400 | -0.75637500 | 2.00183900 |
| H | -7.73657900 | -2.53155400 | 0.69643800 |
| C | 3.39768900 | 0.76438500 | 0.52420600 |
| H | 3.39096000 | 1.00996500 | 1.59471800 |
| H | 4.22963700 | 0.07224000 | 0.36692700 |
| C | 3.63842500 | 2.03157700 | -0.27551500 |
| C | 3.55245200 | 3.29626800 | 0.32275400 |
| C | 3.97507600 | 1.95679400 | -1.63739500 |
| C | 3.79142000 | 4.45973300 | -0.41844300 |
| H | 3.29625600 | 3.37277300 | 1.37766900 |
| C | 4.21320200 | 3.11521200 | -2.38195900 |
| H | 4.05381700 | 0.98284400 | -2.11739300 |
| C | 4.12105300 | 4.37330000 | -1.77431200 |
| H | 3.71949000 | 5.43134100 | 0.06540900 |
| H | 4.47479800 | 3.03638500 | -3.43492200 |
| H | 4.30866000 | 5.27551200 | -2.35185000 |
| C | 3.60796900 | -2.22950200 | 1.71881400 |
| C | 3.25712300 | -3.47825100 | -0.34382000 |
| C | 5.03305000 | -2.76668000 | 1.86122100 |
| H | 2.90463400 | -2.89068100 | 2.25878600 |
| H | 3.52685300 | -1.23952500 | 2.17262700 |
| C | 4.67699000 | -4.05055600 | -0.27861700 |
| H | 2.56083600 | -4.18257400 | 0.14740100 |
| H | 2.94089600 | -3.37244900 | -1.38669200 |
| C | 5.18278400 | -4.12689800 | 1.16797900 |
| H | 5.27465600 | -2.84652800 | 2.92926200 |
| H | 5.73513500 | -2.04363400 | 1.42254900 |
| H | 4.67421200 | -5.04622700 | -0.74122500 |
| H | 5.34702800 | -3.41723400 | -0.87691400 |
| H | 6.22904800 | -4.45812400 | 1.19139900 |
| H | 4.59748400 | -4.87975500 | 1.71711300 |
| N | 3.20659500 | -2.15203700 | 0.29402000 |
| N | -2.19857400 | 3.10651100 | 0.17762800 |
| N | -6.53542700 | -2.34810400 | -0.97924600 |
| H | -1.32715200 | 1.59998900 | -1.54144600 |

4. Time–resolved fluorescence section

This section presents the experimental fluorescence lifetime results measured via the TCSPC technique. The decay curves obtained for the seven pyrrolo[3,4-*b*]pyridin-5-ones across the three solvents and the solid polystyrene matrix reveal distinct dynamics for each molecule are shown in the figure S7 (see Section 4.1). In all plots, data points are represented by circles, and the corresponding fits are depicted as solid lines. To determine the time constants and pre-exponential factors, fitting was performed using the Levenberg-Marquardt algorithm within the SymPhoTime 64 software. Each curve was fitted to a biexponential function, utilizing a 200 ps instrument response function (IRF) measured via fluorescein quenching with I2. The numerical fitting parameters and their associated uncertainties are summarized in Section 4.2

4.1. Decay curves for pyrrolo[3,4-*b*]pyridin-5-ones in different chemical environments

**Figure S7.** Decays for compounds **1a-g** measured by TCSPC. Solvents and polymeric matrices used for each measurement are specified in the respective figures. The instrument response function (IRF) is explicitly shown.

4.2. Lifetimes parameters for pyrrolo[3,4-*b*]pyridin-5-ones.

The tables S9-S12 show the fitting parameters of the experimental data of the pyrrolo[3,4-*b*]pyridin-5-ones using two exponentials. The pre-exponential factors are shown as percentages, and the lifetimes are in ns. The average lifetimes (τ_ave_) were obtained by weighting the lifetimes and pre-exponential factors according to equation S1. This is the intensity-weighted average lifetime and represents the total contribution of each emissive species to the decay curves. The average lifetime τ_ave_ is directly related to the fluorescence quantum yield (Φ_f_) via the radiative rate constant (k_r_), according to the equation S2.

**Equation S1**. $\sum\frac{\mathbf{a}_{\boldsymbol{i}}\mathbf{*}\boldsymbol{\tau}_{\boldsymbol{i}}^{\boldsymbol{2}}}{\mathbf{a}_{\boldsymbol{i}}\boldsymbol{*}\boldsymbol{\tau}_{\boldsymbol{i}}}$

**Equation S2**. (k_r_), Φ_f_ = k_r_ /τ_ave_

**Table S9.** Fit parameters from the TCSPC of the S1 of Pyrrolo[3,4-*b*]pyridin-5-ones in MeOH

| **Molecule** | **%a_1_ (ns)** | **τ_1_** | **%a_2_** | **τ_2_ (ns)** | **τ_ave_ (ns)** | **χ2** |
| --- | --- | --- | --- | --- | --- | --- |
| **1a** | 81.8±2.2 | 0.8±0.2 | 18.2±1.0 | 5.1±0.2 | 3.4±0.2 | 0.995 |
| **1b** | 60.7±1.5 | 1.2±0.2 | 39.3±0.4 | 6.2±0.2 | 5.1±0.2 | 1.213 |
| **1c** | 32.1±1.6 | 1.0±0.2 | 67.9±0.6 | 8.5±0.2 | 8.1±0.2 | 1.060 |
| **1d** | 45.0±1.9 | 1.1±0.2 | 55.0±0.5 | 8.5±0.2 | 7.8±0.2 | 1.201 |
| **1e** | 38.4±3.1 | 0.7±0.2 | 61.6±0.7 | 8.8±0.2 | 8.4±0.2 | 0.998 |
| **1f** | 41.5±2.1 | 0.3±0.2 | 58.5±0.6 | 6.9±0.2 | 6.8±0.2 | 1.192 |
| **1g** | 43.1±1.6 | 1.1±0.2 | 56.9±1.7 | 5.5±0.2 | 4.9±0.2 | 1.290 |

**Table S10.** Fit parameters from the TCSPC of S1 of pyrrolo[3,4-*b*]pyridin-5-ones in MeCN

| **Molecule** | **%a_1_ (ns)** | **τ_1_** | **%a_2_** | **τ_2_ (ns)** | **τ_ave_ (ns)** | **χ2** |
| --- | --- | --- | --- | --- | --- | --- |
| **1a** | 14.8±0.4 | 1.0±0.2 | 85.2±0.2 | 11.1±0.2 | 10.9±0.2 | 1.170 |
| **1b** | 68.2±1.0 | 2.0±0.2 | 31.8±0.4 | 12.0±0.2 | 9.4±0.2 | 0.995 |
| **1c** | 48.4±0.7 | 3.3±0.4 | 51.6±0.7 | 11.2±0.2 | 9.5±0.3 | 1.152 |
| **1d** | 59.7±1.2 | 1.8±0.2 | 40.3±0.5 | 11.0±0.2 | 9.3±0.2 | 1.273 |
| **1e** | 33.7±1.0 | 2.4±0.3 | 66.3±0.8 | 12.5±0.2 | 11.6±0.3 | 0.993 |
| **1f** | 23.1±0.7 | 2.8±0.3 | 76.9±0.5 | 12.8±0.2 | 12.2±0.3 | 1.039 |
| **1g** | 48.5±0.9 | 1.8±0.2 | 51.5±0.5 | 10.7±0.2 | 9.5±0.2 | 1.048 |

**Table S11.** Fit parameters from the TCSPC of the S1 of pyrrolo[3,4-*b*]pyridin-5-ones in toluene

| **Molecule** | **%a_1_** | **τ_1_ (ns)** | **%a_2_** | **τ_2_ (ns)** | **τ_ave_ (ns)** | **χ^2^** |
| --- | --- | --- | --- | --- | --- | --- |
| **1a** | 77.1±3.1 | 1.3±0.2 | 22.9±0.7 | 7.9±0.2 | 5.6±0.2 | 1.049 |
| **1b** | 82.2±2.9 | 1.1±0.2 | 17.8±0.6 | 7.2±0.2 | 4.7±0.2 | 1.228 |
| **1c** | 59.3±1.2 | 1.3±0.2 | 40.7±1.3 | 6.7±0.2 | 5.5±0.2 | 1.159 |
| **1d** | 81.5±3.3 | 1.1±0.2 | 18.5±0.7 | 8.4±0.2 | 5.7±0.2 | 0.998 |
| **1e** | 78.3±2.2 | 1.0±0.2 | 21.7±0.7 | 7.1±0.2 | 5.1±0.2 | 1.129 |
| **1f** | 76.3±3.5 | 0.3±0.2 | 23.7±1.8 | 5.9±0.2 | 5.2±0.2 | 0.992 |
| **1g** | 54.1±0.8 | 1.5±0.2 | 45.9±0.6 | 6.9±0.2 | 5.8±0.2 | 0.997 |

# Table S12. Fit parameters from the TCSPC of the S_1_ of pyrrolo[3,4-*b*]pyridin-5-ones in polystyrene matrix

| **Molecule** | **%a_1_** | **τ_1_ (ns)** | **%a_2_** | **τ_2_ (ns)** | **τ_ave_ (ns)** | **χ^2^** | **%Φ_f_** |
| --- | --- | --- | --- | --- | --- | --- | --- |
| **1a** | 80.1±2.4 | 0.7±0.2 | 19.9±0.3 | 11.6±0.5 | 9.5±0.2 | 1.243 | 29.54±9.70 |
| **1b** | 83.8±2.3 | 1.0±0.3 | 16.2±0.5 | 10.3±0.4 | 7.2±0.2 | 1.133 | 15.57±3.80 |
| **1c** | 86.7±2.1 | 1.5±0.4 | 16.8±0.5 | 13.2±0.5 | 8.9±0.2 | 1.064 | 16.72±7.00 |
| **1d** | 67.5±2.4 | 1.2±0.2 | 32.5±1.4 | 8.9±0.2 | 7.2±0.2 | 1.191 | 13.43±6.50 |
| **1e** | 90.1±2.1 | 0.5±0.2 | 9.7±0.4 | 11.0±0.2 | 7.9±0.2 | 1.166 | 12.87±4.40 |
| **1f** | 91.7±2.5 | 0.2±0.2 | 8.3±0.6 | 10.3±0.2 | 8.5±0.2 | 1.228 | 8.23±2.90 |
| **1g** | 68.9±0.9 | 1.4±0.3 | 31.1±1.2 | 11.0±0.2 | 8.8±0.2 | 1.354 | 22.26±5.90 |

5. Solid-State measurements section.
   5.1. TRPL spectroscopy and PL in Solid-State.

PL measurements were obtained in an Edinburgh Instruments FS5 fluorometer with a 150 W CW Ozone-free xenon arc lamp, and Czerny–Turner monochromators, using an SC-10 solid-state holder. Fluorescence lifetimes were determined from the TRPL spectra. Data obtained from the decay spectra were globally fitted in Fluoracle software from Edinburgh Instruments, using the multi-exponential equation S3 to describe the fluorescence emission decay curve, through a reconvolution analysis with the instrument response function (IRF) measured under the same experimental conditions:

**Equation S3**. $R\left( t \right)=a{}_{1}e^{\left( \frac{-t}{\tau_{1}} \right)}+a{}_{2}e^{\left( \frac{-t}{\tau_{2}} \right)}+a{}_{3}e^{\left( \frac{t}{\tau_{3}} \right)}$

where R(t) represents the fluorescence intensity as a function of time; a_1_, a_2_ and a_3_ are the amplitudes of the respective decay components; and τ_1_, τ_2_ and τ_3_ are the lifetimes of the different components. Additionally, the χ^2^ value is reported.

**Table S13.** Fluorescence lifetime in solid-state experiments

| **Molecule** | **λ_det_ (nm)** | **%a_1_** | **τ_1_ (ns)** | **%a_2_** | **τ_2_ (ns)** | **%a_3_** | **τ_3_ (ns)** | **τ_ave_ (ns)** | **χ2** |
| --- | --- | --- | --- | --- | --- | --- | --- | --- | --- |
| **1a** | 550 | 20.7 | 0.7 | 43.2 | 2.4 | 36.1 | 7.6 | 6.0 | 1.139 |
| **1b** | 520 | 35.4 | 0.6 | 43.9 | 2.3 | 20.7 | 8.1 | 5.5 | 1.053 |
| **1c** | 520 | 33.2 | 0.9 | 36.1 | 2.8 | 30.7 | 8.0 | 6.0 | 1.194 |
| **1d** | 520 | 36.2 | 0.3 | 50.1 | 1.0 | 13.7 | 4.2 | 2.5 | 1.187 |
| **1e** | 500 | 39.3 | 0.1 | 28.7 | 1.3 | 32.0 | 7.2 | 6.3 | 1.384 |
| **1f** | 425 | 11.1 | 0.4 | 14.2 | 1.9 | 74.7 | 9.3 | 9.0 | 1.090 |
| **1g** | 500 | 24.5 | 0.9 | 52.3 | 2.8 | 23.2 | 7.3 | 4.9 | 1.014 |

The λ_det_ values for all compounds correspond to the emission maxima at which the decay profiles were recorded. The average lifetimes (τ_ave_) were obtained by weighting the lifetimes and pre-exponential factors according to the equation S4.

**Equation S4**. $\sum\frac{\mathbf{a}_{\boldsymbol{i}}\mathbf{*}\boldsymbol{\tau}_{\boldsymbol{i}}^{\boldsymbol{2}}}{\mathbf{a}_{\boldsymbol{i}}\boldsymbol{*}\boldsymbol{\tau}_{\boldsymbol{i}}}$.

5.2. Quantum Yields (%Φ_f_).

The %Φ_f_ measurements were carried out using an Edinburgh Instruments FS5 fluorimeter equipped with a continuous-wave 150 W ozone-free xenon arc lamp at room temperature. For solid-state measurements, the system was coupled with an SC-30 solid-state integrated sphere with a PTFA holder. These measurements were performed using the direct method. As an example, Figures S8-S14 show the spectra of compounds**1a-g** obtained directly from the Fluoracle software.

**Figure S8**. Absolute quantum yield of compound **1a** in the solid state, at λ_ex_ = 400 nm.

**Figure S9**. Absolute quantum yield of compound **1b** in the solid state, at λ_ex_ = 400 nm.

**Figure S10**. Absolute quantum yield of compound **1c** in the solid state, at λ_ex_ = 400 nm.

**Figure S11**. Absolute quantum yield of compound **1d** in the solid state, at λ_ex_ = 350 nm.

**Figure S12**. Absolute quantum yield of compound **1e** in the solid state, at λ_ex_ = 350 nm.

**Figure S13**. Absolute quantum yield of compound **1f** in the solid state, at λ_ex_ = 350 nm.

**Figure S14**. Absolute quantum yield of compound **1g** in the solid state, at λ_ex_ = 400 nm.

5.3. Steady-state data for pyrrolo[3,4-*b*]pyridin-5-ones in polystyrene matrix

**Figure S15.** Steady state spectra for 1a–g compounds in polystyrene matrix. A) Normalized absorption spectra at 300 nm. B) Normalized fluorescence emission spectra. Excitation wavelength 355 nm.

**Table S14.** Photophysical properties of the pyrrolo[3,4-*b*]pyridin-5-ones compounds in polystyrene matrix.

| **molecule** | $\boldsymbol{\lambda}$**_onset_**  **(nm)** | $\boldsymbol{\lambda}$**_max_**  **(nm)** | $\boldsymbol{\lambda}$**_fluo_**  **(nm)** | $\bar{\boldsymbol{\nu}}$**_onset_**  **(cm^-1^)** | $\bar{\boldsymbol{\nu}}$**_max_**  **(cm^-1^)** | $\bar{\boldsymbol{\nu}}$**_fluo_**  **(cm^-1^)** | **Stokes Shift** | |
| --- | --- | --- | --- | --- | --- | --- | --- | --- |
|  |  |  |  |  |  |  | $\bar{\boldsymbol{\nu}}$**_onset_ -**$\bar{\boldsymbol{\nu}}$**_fluo_**  **(cm^-1^)** | $\bar{\boldsymbol{\nu}}$**_max_ -**$\bar{\boldsymbol{\nu}}$**_fluo_**  **(cm^-1^)** |
| **1a** | 380 | 318 | 458 | 26320 | 31450 | 21830 | 4490 | 9620 |
| **1b** | 383 | 326 | 491 | 26110 | 30670 | 20370 | 5740 | 10300 |
| **1c** | 388 | 333 | 454 | 25770 | 30030 | 22030 | 3740 | 8000 |
| **1d** | 409 | 341 | 469 | 24450 | 29330 | 21320 | 3130 | 8010 |
| **1e** | 371 | 317 | 457 | 26950 | 31550 | 21880 | 5070 | 9670 |
| **1f** | 410 | 322 | 447 | 24390 | 31060 | 22370 | 2020 | 8690 |
| **1g** | 377 | 330 | 437 | 26530 | 30300 | 22880 | 3650 | 7420 |

6. Supplementary References

The structural characterization of compounds **1a-g** was previously reported in:

[S1] Morales-Salazar, I.; Montes-Enríquez, F.P.; Garduño-Albino, C.E.; García-Sánchez, M.A.; Ibarra, I.A.; Rojas-Aguirre, Y.; García-Hernández, M.E.; Sarmiento-Silva, R.E.; Alcaraz-Estrada, S.L.; Díaz-Cervantes, E.; González-Zamora, E.; Islas-Jácome, A. Synthesis of bis-furyl-pyrrolo[3,4-*b*]pyridin-5-ones *via* Ugi–Zhu reaction and *in vitro* activity assays against human SARS-CoV-2 and *in silico* studies on its main proteins. *RSC Med. Chem.* **2023**, *14*, 154–165. DOI: 10.1039/d2md00350c.

[S2] Morales-Salazar, I.; Rincón-Guevara, M.A.; González-Zamora, E.; Islas-Jácome, A. 2-Benzyl-3-morpholino-7-(thiophen-2-yl)-6-(thiophen-2-ylmethyl)-6,7-dihydro-5*H*-pyrrolo[3,4-*b*]pyridin-5-one. *Molbank* **2022***, 4*, M1503. DOI: 10.3390/M1503.

[S3] Morales-Salazar, I.; Garduño-Albino, C.E.; Montes-Enríquez, F.P.; Nava-Tapia, D.A.; Navarro-Tito, N.; Herrera-Zúñiga, L.D.; González-Zamora, E.; Islas-Jácome, A. Synthesis of pyrrolo[3,4-*b*]pyridin-5-ones *via* Ugi–Zhu reaction and *in vitro*–*in silico* studies against breast carcinoma. *Pharmaceuticals* **2023**, *16*, 1562. DOI: 10.3390/ph16111562.
